# Candidate name order effects in New Hampshire: Evidence from primaries and from general elections with party column ballots

**DOI:** 10.1371/journal.pone.0248049

**Published:** 2021-03-16

**Authors:** Bo MacInnis, Joanne M. Miller, Jon A. Krosnick, Clifton Below, Miriam Lindner

**Affiliations:** 1 Department of Communication, Stanford University, Stanford, California, United States of America; 2 Department of Political Science and International Relations, University of Delaware, Newark, Delaware, United States of America; 3 City Councilor, Lebanon, New Hampshire, United States of America; 4 Department of Psychology, Harvard University, Cambridge, Massachusetts, United States of America; Sogang University (South Korea), REPUBLIC OF KOREA

## Abstract

Research in a few U.S. states has shown that candidates listed first on ballots gain extra votes as a result. This study explored name order effects for the first time in New Hampshire, where such effects might be weak or entirely absent because of high political engagement and the use of party column ballots. In general elections (in 2012 and 2016) for federal offices and the governorship and in primaries (in 2000, 2002, and 2004), evidence of primacy effects appeared in 86% of the 84 tests, including the 2016 presidential race, when Donald Trump gained 1.7 percentage points from first listing, and Hillary Clinton gained 1.5 percentage points. Consistent with theoretical predictions, primacy effects were larger in primaries and for major-party candidates in general elections than for non-major-party candidates in general elections, more pronounced in less publicized contests, and stronger in contests without an incumbent running. All of this constitutes evidence of the reliability and generalizability of evidence on candidate name order effects and their moderators.

## Introduction

In democratic nations, the integrity of the electoral process is essential to the functioning, stability, and legitimacy of government [1]. At various moments in history, the legitimacy of the electoral process has been of concern to Americans, and trust in that process has been a determinant of public acceptance of election winners [2]. Between 2006 and 2016, Americans became increasingly uncertain of election fairness: the percentage of Americans who said they did not have confidence in the honesty of elections rose from 47% to 69% [3]. And in 2018, only 51% of Americans believed that their elections are fair and open [4]. This crisis of confidence in the integrity of U.S. elections has spurred research and media speculation about reforms that might bolster public confidence. This paper offers new evidence encouraging one such reform; candidates listed first gain an electoral advantage, so rotation of candidate names across voters would enhance electoral fairness.

We begin below by outlining the theory of name order effects, moderators of name order effects, and findings of past studies of name order effects. Next, we highlight the opportunity afforded by a 2006 court ruling to test for such effects in a new and interesting context, New Hampshire, which might be viewed as a “limiting case.” We describe the results of analyses of the effect of name order in both primary and general elections in that state—including the 2016 presidential election—and describe tests of moderators in this context. Finally, we discuss both the practical implications of the findings and their implications for theories of voter decision-making and behavior.

## Theory of name order effects

Psychological theory suggests two possible explanations for name order effects in elections. One theory begins by noting the tremendous burden levied on voters in the context of American democracy, where people may feel that being a “good and responsible democratic citizen” requires them not only to go to the polls but also to cast votes in all listed races, even when they know only a little about the candidates or have not made a firm choice among them before entering the voting booth.

In California, for example, citizens have routinely been asked to vote on a dozen ballot issues on topics ranging from insurance reforms to tort claims to school funding to the confidentiality of AIDS tests [5]. And in all states, voters have sometimes been asked to make choices in dozens of races, ranging from high visibility contests to races for offices so obscure that many voters probably could not describe the job responsibilities associated with them. In 1911, for instance, Cleveland, Ohio voters were confronted with 74 candidates for city offices, 12 candidates for Board of Education, 14 candidates for Municipal Court Judges, and 32 candidates for Constitutional Convention [6]. Eighty years later, in 1992, Cleveland voters were asked to cast ballots in over 40 county and statewide races, plus a number of district-wide races.

Because races for highly visible offices (e.g., U.S. President and U.S. Senate) receive a great deal of news media attention, often involve well-known incumbents, and usually involve explicit endorsements of candidates by political parties, voters who wish to make substance-based choices can do so in principle. But in many contests, candidates do not take clear and divergent stands on specific policy issues [7], and media coverage of such contests has often focused on the horserace rather than on the candidates’ records and policy positions [8]. The cognitive demands of sifting through lots of such media coverage and extracting useful, substantive information about candidates’ positions may therefore be so substantial as to outstrip some voters’ incentives to do the work [9]. Much research suggests that under such circumstances, many citizens rely on only a small subset of substantive information to make such vote choices, pursing what Popkin called “low information rationality” [10].

Media coverage of races for less visible offices (e.g., Attorney General, Auditor, Judge, Sheriff, Coroner, and Board of Education) has often been much more limited, making it even more difficult for voters to make choices based upon substance [11]. Voters pursuing low information rationality can sometimes rely on cues, such as party affiliation, which can help them identify candidates with whom they are likely to agree on policy issues [12]. But party affiliations are often not listed on the ballot for the very races that receive the least media coverage.

Alternatively, people can rely upon name recognition: the candidate whose name sparks a stronger sense of familiarity is most likely to be the incumbent, who by virtue of his or her presumed experience may be considered the safer choice [13]. But because holders of low-visibility offices probably get very little media attention during their tenures, voters may only rarely recognize their names.

What do people do when no such cues are present to guide their choices? In some years, large numbers of people have gone to the polls to vote in a few highly visible contests, yet they were asked to vote in less publicized races, too. The higher roll-off rates typical of such races presumably reflect some voters’ choices to abstain because they lack sufficient knowledge [14]. But many voters may feel compelled to vote in races about which they lack information. A person doing so may be especially susceptible to being “nudged” toward the first-listed candidate, because even when people are instructed to select randomly among a set of offered alternatives, they are inclined to select the option they consider first [15].

But even among people who do have some information about a race, the well-known “confirmatory bias” in reasoning may cause a primacy effect. When evaluating a set of objects in order to select one of them, people usually begin a search of memory for information about each object by looking for reasons to select it, rather than reasons not to select it [16]. So when considering a list of political candidates, voters may first search memory primarily for reasons to vote for each contender rather than reasons to vote against him or her. And when working through a list of candidates, people may think less and less about each subsequent alternative, because they become increasingly fatigued, and short-term memory becomes increasingly clogged with thoughts. Therefore, people may be more likely to generate supportive thoughts about candidates listed initially and less likely to do so for later-listed candidates, biasing them toward voting for the former.

In theory, people attempting to retrieve reasons to vote for a candidate may occasionally fail completely, retrieving instead only reasons to vote against him or her. If this happens for all candidates in a given race, cognitive fatigue and short-term memory congestion would presumably bias a citizen toward generating more reasons to vote against the first-listed candidate than reasons to vote against later-listed candidates. This would induce a recency effect, which is a bias toward selecting candidates listed last [17].

Name order might also influence the votes cast by people who have no information at all about the candidates in a race but nonetheless feel compelled to vote in all races in order to be “good citizens.” According to Simon [18], people are inclined to settle for the first acceptable solution to a problem they confront, especially when they perceive that the costs of making a mistake will be minimal [18]. Therefore, if a citizen feels compelled to vote in races for which he or she has no substantive basis for choice at all, he or she may simply settle for the first name listed, because no reason is apparently suggesting that the candidate is unacceptable.

All of the above logic can be thought of as attributing name order effects to “information deficit.” But name order effects might also occur under very different conditions: when voters are very well informed. Ambivalence towards candidates is not uncommon. For example, one study suggested that about 30% of the electorate held ambivalent attitudes toward the major American political parties [19]. As would be expected, more ambivalent citizens take longer to crystalize their preferences [20]. Consider a voter who has devoted great effort to learning about candidates competing for President of the United States and has discovered an array of reasons to vote for and against each one. When he or she finally walks into a voting booth or confronts an absentee ballot, making a choice between the candidates might be very difficult, because their pros and cons nearly balance out. As a result, when under pressure to make a choice and move on with life, name order might again constitute a nudge, yielding a bias toward the first-listed name. Thus, name order effects might occur due to ambivalence, even when voters have access to lots of information about the candidates and even when party affiliations are specifically listed for each candidate.

## Past studies of name order effects

During the last 70 years, a sizeable number of studies have investigated name order effects in a wide array of elections across many countries. As we explain below, this work has consistently identified primacy effects occurring in the vast majority of races examined: candidates received more votes when they were listed first than when they were listed later (for reviews, see [21, 22]). While reviewing the findings of some past studies below, we highlight the fact that the strongest evidence of name order effects in the U.S. comes from the study of elections in a few geographic areas. Therefore, our understanding of these effects can be enhanced by exploring the generalizability of those published findings to new geographic regions, especially ones where we might imagine name order effects would be weaker than usual.

### Studies of candidate name order effects in U.S. general elections

Many studies have documented primacy effects in general elections in the U.S. [23–35]. For example, Miller and Krosnick found that 86% of 196 name order effect tests in the three largest Ohio counties in 1992 manifested differences in the direction of primacy (each two-candidate race yielded one name order test, and each race with more than two candidates yielded one test for each candidate). Statistically significant primacy effects appeared in 48% of races, and the significant effects averaged 2.33 percentage points [31, pp. 308, 315].

In elections in Ohio, California, and North Dakota in 2000, Krosnick, Miller, and Tichy found that 129 of 170 tests of name order effects in two-candidate races (76%) manifested vote count differences in the direction of primacy, and 113 of the 136 candidates (83%) in races with more than two candidates manifested differences in the direction of primacy, yielding 79% of name order effects in the direction of primacy overall (p < .001).

Pasek et al. found that more than 93% of 402 candidates in California general elections held between 1976 and 2006 received more votes when listed first than when listed later (p < .001) [34, p. 425]. Ho and Imai [29] identified fewer primacy effects in their study of some of the same races as Pasek et al. [34] but analyzed a data set that contained a substantial number of data entry errors (which was corrected in [34]) and did not control for party registration of the assembly districts (which was performed in [34]). The latter is critically important, because with only 80 assembly districts in California and large numbers of candidates per race, unintentional confounds of party leanings of districts with name orders occurred and needed to be eliminated via statistical controls.

In statewide elections in North Dakota between 2000 and 2006, Chen et al. [27] reported differences in the direction of primacy in 75% of 36 races (p < .001); the average effect in two candidate races was 1.17 percentage points. Across thousands of candidates in municipal general elections in Peoria, Illinois, between 1983 and 1999, Brockington [25] found a statistically significant meta-analytic primacy effect, evidenced by a decrease of 0.68 percentage points for each shift of position away from being listed first. Older studies of general elections produced similar evidence of primacy effects [26, 32, 33, 35].

Only one study of general elections failed to find evidence suggesting a tendency toward primacy effects, but those elections involved an unusual type of ballot: party column ballots, where all of the candidates from each party are listed in a single column for that party across all races, and the order of the columns is varied across voters. In two Colorado counties in 1984, Darcy [36] found that only about half of candidates (12 out of 22) in two-candidate races manifested name order effects in the direction of primacy, not significantly greater than 50% (p = .25) (This is based on numbers in Table 2 in [36], p. 658–659).

In sum, many studies conducted during the past six decades have documented strong trends toward primacy effects in many general elections in the U.S.

### Studies of name order effects in primaries in the U.S.

A few studies of primary elections have also uncovered evidence of primacy effects [25, 29, 37–41]. For example, Koppell and Steen [40] found that being listed first in the 1998 Democratic primary in New York City yielded more votes for 89% of 180 candidates examined, a percentage significantly greater than 50% (p < .001). Primacy effects were statistically significant or marginally significant in 21 of 79 races, averaging 6.86 percentage points, with a maximum of 11.30 percentage points (calculated based on Tables 1 and 2 in [40], pp. 272–275; a copy of Tables 1 and 2 is reproduced in S1 and S2 Figs in S1 Text, respectively). Grant [39] found that 100% of candidates in Democratic and Republican primaries in Texas in 2014 manifested name order effects in the direction of primacy, and all primacy effects were statistically significant, averaging 5.32 percentage points, with a maximum of 10.48 percentage points (calculated based on Table 4 in [39], pp. 422–423; a copy of Table 4 is reproduced in S3 Fig in S1 Text).

Brockington [25] found statistically significant primacy effects across thousands of candidates in municipal primary elections in Illinois, averaging a decrease of 1.65 percentage points for each shift of position away from being listed first [25, p. 16]. Older studies of primaries also documented statistically significant primacy effects, such as in the 1920 primary election in Pennsylvania [37], in the 1948 Republican primary in the Ohio Senate [41], in the 1948 Ohio and 1952 Michigan primaries [23].

### Theory: When name order effects are expected to occur

Based upon the information deficit explanation and the ambivalence explanation, it is possible to derive a series of predictions about when name order effects may be greatest in elections. These predictions are derived from the fundamental assumption that primacy effects occur because some voters are unable, unwilling, too overwhelmed, too ambivalent, or simply unmotivated to reach an optimal, unbiased candidate choice.

First, the more information voters have with which to make substance-based choices between candidates, the less likely they are to be influenced by name order. Information about candidates is routinely provided to voters by news media coverage of a race, but the amount of such coverage varies considerably across races. Races about which citizens learn a great deal are presumably those in which name order effects will be smallest.

A second type of information is simply the party affiliations of the candidates. Considerable evidence shows that voters use candidate partisanship as a heuristic cue in their decision-making [42]. Because party affiliations of candidates help voters to make information-based choices in partisan general elections without other substantive knowledge about the candidates, indecision may be less common regarding those contests [43]. In contrast, in non-partisan general election contests and primaries, where party cues are absent, voters lacking candidate-specific knowledge are not given easy-to-use devices to select among the listed competitors. Therefore, non-partisan general election contests and primaries may be particularly prone to candidate name order effects [29, 31, 44].

Third, voters sometimes have years to watch an incumbent in office before he or she is running for re-election. In such situations, voters are likely to have substantially more information about that candidate than is gained simply by news coverage of the election itself. Therefore, races involving incumbents may manifest weaker name order effects.

Voters who are more interested in politics and more attentive to political news are presumably more exposed to the flow of political information and are presumably more likely to retain in memory the information to which they are exposed. If information volume reduces susceptibility to name order effects, these individuals may therefore be less likely to be nudgeable in this way.

Because information about politics can often be complex and technical, we might imagine that name order effects will be weaker among citizens with more cognitive skills (i.e., the ensemble of abilities that enable interpreting incoming information, storing it in memory, retrieving the information later, and integrating the retrieved information in order to select between candidates), because they can more easily manage the information processing of making substance-based choices between candidates.

Party affiliations of the candidates might influence the size of name order effects in another way as well. Among independent voters who do not have a standing preference for one major party over the other, party affiliations of candidates may not be helpful for choosing between such competitors. But affiliations with major political parties differentiate some candidates from others on the ballot who lack such affiliations. Therefore, for independent voters, candidate affiliations with major parties may be signals of viability, because these candidates were endorsed and supported by large organizations with considerable resources. Therefore, a nudge in the direction of such a candidate may be more well-received by a voter than a nudge toward a minor party candidate about whom the voter knows little.

Expected margin of victory might be another moderator. When one candidate is likely to beat the others by a large margin, voters may have little incentive for careful consideration when casting their votes. In contrast, for close contests, voters might be motivated to pay careful attention to their candidate choice [45]. Because of this enhanced motivation to gather information and think carefully about it, candidate name order may influence fewer individuals in close contests than would be the case in electoral blowouts.

Lastly, consistent with the ambivalence mechanism, a voter who has a more difficult time choosing between competing candidates because he or she evaluates the two similarly and/or perceives both to have strengths and weaknesses may be especially nudgeable. In contrast, a voter who clearly prefers one candidate over others may be less susceptible to the influence of candidate name order.

## Past studies of the moderators of name order effects

A number of studies have explored when name order effects are greatest in elections and have generated evidence in support of the hypotheses articulated above. Stronger name order effects were found for less publicized races [31, 34, 40]. Weaker name order effects were found for races in which an incumbent was running for re-election, who was presumably familiar to many voters [27, 31]. Name order effects were also stronger in general elections when candidate party affiliations were not listed on the ballot next to their names [27, 34]. Stronger name order effects were found among less educated voters as well [24, 34, 40].

As expected, weaker name order effects were found in races with small margins of victory, when voters might have thought their vote would make a more notable difference in determining the election outcome [34]. And especially strong name order effects were found in in low-visibility races with higher turnout, which presumably attracted more voters lacking knowledge about those more obscure races [34]. Stronger name order effects were also found in years when turnout was higher [27].

Additionally, name order effects were notably larger in the 2004 Presidential race in Ohio among voters who voted on touch screens (four percentage points for George W. Bush and for John Kerry) than when voters used paper ballots or punch cards [24]. One possible reason for this is that perhaps touch screen machines encourage voters to vote in all races, whereas other modes of voting, because they are passive, do not prompt voting in all races.

## The case of New Hampshire

### History of New Hampshire elections

Because most of this past evidence of primacy effects in general elections in the U.S. comes from studies of a handful of states (California, Ohio, and North Dakota), it is valuable to test the generalizability of those findings to other states. And New Hampshire offers a uniquely valuable opportunity to do so, for a variety of reasons. Like California, Ohio, and North Dakota, New Hampshire has rotated candidate name order in general and primary elections in ways that constitute quasi-experiments that afford the opportunity to make strong causal inferences about the impact of name order.

In 1979, New Hampshire began rotating candidate name order in primary elections, pursuant to RSA 656:24:

**656:24 Order of Names**: With the exception of the office of state representative, whenever there are 2 or more candidates for nomination to the same office, the names of such candidates shall be alternated on the state primary election ballots used so that each name shall appear thereon as nearly as may be an equal number of times at the top, at the bottom, and in each intermediate place, if any, of the list in which it belongs.

This rotation requirement applied to presidential primaries until 2004, when it was stopped in order to reduce the cost of printing ballots [46]. Legislation enacted in 2015 re-established name order rotation in presidential primaries [47].

Name order rotation in New Hampshire general elections began after the New Hampshire Supreme Court ruled in 2006 [48] that the lack of rotation was unconstitutional (see also the recent Florida District Court ruling in Jacobson v. Lee, which concluded that a single order based on the party of the candidates was unconstitutional [49]). Party columns were rotated in the 2006 and 2008 general elections across only 24 state Senate districts, meaning there was insufficient statistical power to detect any name order effects [50]. Legislation was enacted in 2010 that specified a party column rotation scheme for the 2010 election and set forth the procedure described below for subsequent elections.

Since 2010, candidate name order has been rotated across New Hampshire’s 323 towns, city wards, and unincorporated places (constituting what are called “voting places” in the state). Candidate names in general elections starting in 2012 were rotated by party column under the statute NH RSA 656:5 (See S1 Text for more detail).

### Other reasons to study New Hampshire elections

Studying the effect of name order on vote counts in New Hampshire is valuable for reasons other than the use of party column ballots there and the opportunity to test generalization outside of Ohio, California, and North Dakota. Specifically, there are reasons to expect that name order effects might be minimal or non-existent in New Hampshire in light of evidence that primacy effects have been stronger in contests that received less media attention [31, 34, 51] and among less educated and less politically engaged voters [24, 25, 31, 52, 53].

Because New Hampshire has routinely hosted the nation’s first primary during each presidential election season, candidates have spent large amounts of time and money to campaign in the state, per capita. And they have been the subject of extensive news media attention during the primary season. Therefore, the residents of the state have been given remarkably large amounts of information about all the competitors during the primaries, which makes those voters especially well-informed at the moment that the general election campaign begins there. Thus, these voters have a considerable head start and are likely to find themselves on election day with tremendous amounts of information about the candidates.

Moreover, New Hampshire voters probably feel a great deal of responsibility for, and take great pride in, setting the stage for later primaries. So, there is reason to believe that voters there are especially aware of, knowledgeable about, and engaged with politics, which might, in theory, minimize name order effects.

Consistent with this logic, turnout in the voting eligible population [48] in the 2016 New Hampshire primaries was 52.5%, the highest of the primary states. In the 2016 general election, turnout in New Hampshire was 72.5 percent, the third highest in all the states [53]. In contrast, the turnout rate for the 2016 general election was 64.2 percent in Ohio, 61.9 percent in North Dakota, and 58.4 percent in California, the states where quasi-experiments on name order were conducted in the past. Therefore, one might expect that name order effects will be muted or non-existent in New Hampshire [54].

In fact, the state’s Attorney General argued during the New Hampshire Supreme Court’s trial in 2006 that “New Hampshire voters are among the best informed, most discerning voters in the country and they are voters who participate in more elections than most other Americans. … [T]he possibility that [some] voters choose candidates based on ballot position is implausible” [55].

Another reason to support such an expectation might be the party column format of general election ballots used in New Hampshire. Such ballots might encourage voters to focus on parties rather than candidates. Among voters who have strong affiliations with one party or another, the party column ballot may thereby focus voters on those affiliations rather than on the specific characteristics of competing candidates. Because party cues may constitute potent heuristics to select candidates with little effort, the party column format may minimize name order effects. This may explain why Darcy [36] found no name order effects in the two Colorado counties he studied, which used such a format, and we might therefore expect weak order effects in New Hampshire.

## The value of studying replication and generalization

Assessing the replicability and generalizability of candidate name order effects dovetails with a general trend across the sciences. In recent years, high-profile failures to replicate wide swathes of published findings in the natural sciences (e.g., [56]) and the social sciences (e.g., [57, 58]), and other fields have documented the so-called “decline effect” [59] and raised questions about the believability of published findings. And other work has illuminated some of the processes that have likely led the published literature to be misleading, including publication bias [60–62] and p-hacking [63]. All this has led researchers to be more guarded before accepting findings as real, generalizable, and meriting practical application until they have been extensively documented. To this end, some journals have changed their practices to explicitly encourage publication of replication and extension studies [64]. The current paper offers the opportunity to do so.

Replication studies in this arena are not easy to do. Obtaining election returns is easy. But determining the order of candidates’ names on ballots is much more challenging, because even in states where rotation is mandated by law, elections officials are not required to maintain publicly available documentation of the name orders that specific voters saw. Therefore, the process of working with officials in multiple County Boards of Elections to determine name orders for use in statistical analyses can be challenging. The present investigation was possible because such reconnaissance was done in a timely fashion.

This investigation also constitutes an important opportunity to enhance the public impact of political science. Public esteem for the discipline is an important potential determinant of the amount of money made available to support the field’s research efforts. And over the years, research on candidate name order effects has spurred a series of efforts to change election procedures in the states to promote fairness by rotating name order across ballots [65]. Some of these lawsuits have been successful in causing states to implement some sort of rotation [66], and others have not. But in all cases of litigation, as noted above, one of the critical objections brought by resisters has been uncertainty about whether evidence of primacy effects in elections in California, Ohio, and North Dakota (where name orders have been rotated and primacy effects have been documented) generalize to other states. Furthermore, there has even been some dissention among academic researchers about the prevalence and nature of primacy effects in elections [29, 36, 67]. Therefore, for this literature to be convincing about the generally practical value of past demonstrations of primacy effects, it must continue to grow with more documentations of the generalizability of the finding to other geographic areas and other ballot designs. The present study offers the opportunity to do just that.

In sum, an examination of the effect of candidate name order on vote counts in New Hampshire has value for both basic and applied research related to electoral processes and electoral reform.

## This investigation

In the investigation described here, we assessed the presence of candidate name order effects in general elections in 2012 and 2016 for federal offices and the governorship and in primaries in 2000, 2002, and 2004. Building on the aforementioned theoretical expectations as to when name order effects may be strongest, we tested whether name order effects were stronger in primary elections than in general elections, in less publicized elections, in elections without incumbents running, in races with large margins of victory, and in the votes for major party candidates than in votes for non-major party candidates.

In the pages that follow, we describe our methodological approach and the results we obtained.

## Data

### Primaries

Among the races examined in 2000, 2002, and 2004 Democratic and Republican primaries were those for U.S. Senate, U.S. House of Representatives, and New Hampshire Governor. We could not analyze the 2000 primary for President because we could not obtain all the necessary data. We could not analyze the 2004 presidential primary because no name rotation was done that year. And we could not analyze the 2010 state-wide primaries because we could not obtain the ballots that were needed to determine which voters saw candidates in which order.

In the races we examined, candidates’ names were rotated across the 323 voting places. The name orders for each party’s primary were assigned to voting places by a computer program. The program first ordered voting places in descending order based on the number of registered voters affiliated with the party. Then, the program rotated candidate names through the list of voting places so that each candidate would appear in each position on ballots administered to approximately the same number of registered voters. Then, if necessary, elections officials made minor swaps of voting places of nearly equal size (based on the number of registered voters in each voting place) so that no candidate would be listed first in a large share of any given city’s wards (see S1 Text for more details about the name order procedure).

The datasets created for this investigation include the order in which each candidate appeared on the ballot in each voting place and the number of votes received by each candidate in each voting place. Data on candidate name order were obtained from Karen Ladd in the New Hampshire Secretary of State’s office in the form of photocopies of town rotation order by party, office, and candidate, as well as by inspection of original official copies of actual ballots. Data were spot-checked between the two sources and revealed no discrepancies.

We examined three primaries held in 2000: two 2-candidate races (the Democratic primary for Governor and the Democratic primary for the 2^nd^ Congressional District) and one 5-candidate race (the Republican primary for Governor). We analyzed seven primaries in 2002: four 2-candidate races (the Democratic primary for Governor, the Democratic primaries for the 1^st^ and 2^nd^ Congressional Districts, and the Republican primary for the 2^nd^ Congressional District), one 3-candidate race (the Republican primary for U.S. Senate), one 6-candidate race (the Republican primary for Governor), and one 8-candidate race (the Republican primary for the 1^st^ Congressional District).

We examined seven primaries held in 2004: five 2-candidate races (the Democratic and Republican primaries for Governor, the Republican primary in the 1^st^ Congressional District, and the Democratic and Republican primaries in the 2^nd^ Congressional District), one 3-candidate race (the Republican primary for U.S. Senate), and one 4-candidate race (the Democratic primary for the 1^st^ Congressional District; see S1 Text for more detailed descriptions of the data for these primaries).

### General elections

We tested for name order effects in statewide general elections in 2012 and 2016 for U.S. President, U.S. Senate, U.S. House of Representatives, and New Hampshire Governor. In those elections, candidate names were rotated by party column under the statute NH RSA 656:5 [68]. The names of all candidates were arranged in three successive party columns, and each column contained the names of the candidates of one party (Democratic, Republican, or Other). The order of these party columns on the ballots was rotated using a computer program to ensure that each party column appeared an approximately equal number of times in the first, second, and third (last) (See S1 Text for more details; see S4 and S5 Figs in S1 Text for a sample copy of the 2012 and 2016 ballots, respectively).

Data were gathered for 13 candidates running in the 2012 general elections (four candidates for President, three for Governor, three for the 1^st^ Congressional District representative, and three for the 2^nd^ Congressional District representative) and for 20 candidates running in the 2016 general elections (five candidates for President, four for the U.S. Senate, three for Governor, five for the 1^st^ Congressional District representative, and three for the 2^nd^ Congressional District for representative).

We constructed general election datasets that included the following variables: (a) the order in which each candidate’s name appeared in each voting place, (b) the number of votes received by each candidate in each voting place, and (c) the number of registered Democrats and the number of registered Republicans in each voting place. The latter two variables were downloaded from the website of the Secretary of State [69].

To determine candidate name order in each voting place, two people independently examined sample ballots and coded the order of the party columns independently. The coders agreed for 99% of the ballots in 2012 (a third coder resolved the disagreements) and for 100% of the ballots in 2016 (see S1 Text for examples of the ballots and more detailed descriptions of the data).

### Moderators of name order effects

To explore moderation of name order effects, a series of variables were constructed. Primary candidates were identified by a dummy variable coded 1 for them; general election candidates were coded 0 (mean = .61, SD = .49, min = 0, max = 1). Another dummy variable was coded 1 for Democratic and Republican party candidates and 0 for other candidates (mean = .21, SD = .41, min = 0, max = 1).

Publicity of a contest was operationalized by the number of articles about each of the candidates in the race published by New Hampshire newspapers during the period starting from six months prior to the day of the election to election day. The count of articles about each candidate was obtained by searching World Access News for newspaper stories (appearing in the following newspapers: *Concord Monitor* of Concord, *The Dartmouth* at Dartmouth College, *Derry News* of Derry, *Eagle Times* of Claremont, *Foster’s Daily Democrat* of Dover, *The Keene Sentinel* of Keene, *New Hampshire Union Leader* of Manchester, *The Telegraph* of Nashua, and *Valley News* of Lebanon) for the candidate’s last name and the office name (i.e. President, Governor, Senator, or Representative). The total number of newspaper articles about all the candidates in a race was then scaled to range from 0 to 1 (mean = .26, SD = .27, min = 0, max = 1), and the square root of which computed to indicate publicity of the contest while minimizing the impact of high outliers (mean = .45, SD = .23, min = 0, max = 1).

Another dummy variable was coded 1 for candidates in contests with an incumbent and 0 for all other contests (mean = .45, SD = .50, min = 0, max = 1). Closeness of the contest was represented by the square root of the margin of victory of the race (mean = .40, SD = .29, min = 0, max = 1), which was the difference between the vote share of the winner and the vote share of the candidate with the next highest vote share (mean = .24, SD = .29, min = 0, max = 1).

## Analysis methods

### Estimating the primacy effects

The effect of name order on vote percentages was gauged separately for each candidate in each race. Because some voters wrote in names of candidates not listed on the ballot, the name order effects for the two candidates in 2-candidate races were not perfect mirror images of one another. Nine analyses were conducted for the 2000 primaries (four analyses for the 2-candidate races and five analyses for the 5-candidate race), 25 analyses were conducted for the 2002 primaries (eight analyses for the 2-candidate races for Democratic primary for Governor, the Democratic primaries for the 1^st^ and 2^nd^ Congressional Districts, and the Republican primary for the 2^nd^ Congressional District; three analyses for the 3-candidate race for the Republican primary for U.S. Senate; six analyses for the 6-candidate race for the Republican primary for Governor; and eight analyses for the 8-candidate race for the Republican primary for the 1^st^ Congressional District), and 17 analyses were conducted for the 2004 primaries (10 for the 2-candidate races for the Democratic and Republican primaries for Governor, the Republican primary in the 1^st^ Congressional District, and the Democratic and Republican primaries in the 2^nd^ Congressional District; three for the 3-candidate race for the Republican primary for U.S. Senate; and four for the Democratic primary for the 1^st^ Congressional District).

Thirteen analyses were conducted for the 2012 general election (four for the 4-candidate Presidential race, three for the 3-candidate Gubernatorial race, three for the 3-candidate 1^st^ Congressional District race, and three for the 3-candidate 2^nd^ Congressional District race), and 20 analyses were conducted for the 2016 general election (five for the 5-candidate race for President, four for the 4-candidate race for U.S. Senate, three each for the 3-candidate races for Governor and the 2^nd^ Congressional District, five for the 5-candidate 1^st^ Congressional District race, and three for the 2^nd^ Congressional District).

Name order was coded 1 when the candidate was listed first on the ballot and 0 when the candidate was listed further down on the ballot.

We used two alternative statistical techniques to assess the robustness of our conclusions. First, we estimated the parameters of ordinary least squares (OLS) regression equations to assess the impact of name order on the percent of votes received by the candidate, controlling for the total number of votes cast in the race in each voting place (divided by 1000), as well as (for general elections) the percent of registered voters registered as Democrats and the percent of registered voters registered as Republicans in each voting place. Although some researchers have raised questions about using OLS to test for name order effects (e.g. [29, 67]), other scholars have demonstrated that OLS, seemingly unrelated regression, and other analytic methods produce comparable results (e.g., [34]).

Next, we examined the effect of name order using non-parametric randomization inference. Randomization inference is based on the seminal work by Sir R.A. Fisher [70], who established the technique as a principled method for statistical inference in randomized experiments (see also [71–73]), and it was used in a recent study of candidate name order effects [74]. An advantage of randomization inference is that it does not require any assumptions about the distributions of variables or large sample sizes or independent random sampling that are required for many other statistical tests. This makes randomization inference a highly credible and robust tool for hypothesis testing in causal inference in many settings [71–73].

The randomization inference analysis entails estimating the parameters of the regression equation 10,000 times and randomly assigning the name order variable for each candidate during each iteration, thus yielding a distribution of expected differences if name order has no effect. The observed effect can then be compared to that distribution to gauge the probability that that observed effect occurred by chance alone, yielding statistical significance tests different from those generated by OLS regression. We report the probabilities obtained from the randomization inference tests that measure the fraction of 10,000 re-randomizations that yielded differences equal to or larger than the observed effect estimates of name order effects.

### Additional robustness checks of the primacy effects

#### Robustness checks for the 2000, 2002, and 2004 primaries

Additional analyses using three alternative specifications of name order yielded evidence of the robustness of the findings about the 2000, 2002, and 2004 primaries. First, we re-ran the analyses for the 29 candidates in the primaries who were competing against more than one candidate using a linear specification of name order that was centered around zero. For example, for a 3-candidate race, order was coded as -1, 0, and 1 when candidates were listed first, second, and third, respectively. And for a 4-candidate race, order was coded as -1.5, -0.5, 0.5, and 1.5 when candidates were listed first, second, third, and fourth, respectively (the name order variable was centered to facilitate a test of whether the effect of name order is non-linear, described next). The primacy hypothesis predicts that the effect of this name order variable on vote percentages will be negative (because the lowest value on the order variable represents the first position, the second lowest represents the second position, and so on).

Second, we tested whether order had a non-linear effect on vote counts (for the 29 candidates running in primaries against more than one candidate) in a manner recommended by Aiken and West [75] and used by Miller and Krosnick [31] in their analysis of name order effects in the 1992 election in Ohio. Specifically, according to Aiken and West [75]: “If the first order variables X and Z are not centered, then product terms of the form XZ and power polynomial terms of the form X^2^ are highly correlated with the variables of which they are comprised (the Pearson product moment correlation between X and X^2^ can approach 1.0). When employed in regression analyses with lower order terms, the highest order term produces large standard errors for the regression coefficients of the lower order terms, though the standard error of the highest order terms is unaffected…The multicolinearity in the context of regression with higher order terms is due to scaling, and can be greatly lessened by centering variables” (pg. 35). Accordingly, we squared the centered linear variable and included it, along with the centered linear term, in a regression equation to assess whether name order effects were curvilinear.

Third, we represented name order for the 29 candidates in primary races with more than two candidates) as a series of dummy variables, with being listed first as the omitted category. One dummy variable was coded 1 when the candidate was listed second and 0 otherwise. Another dummy variable was coded 1 when the candidate was listed third and 0 otherwise, etc.

#### Robustness checks for the 2012 and 2016 general elections

Five robustness checks were conducted with the general election data, using four alternative specifications of name order. The first three robustness checks were the same as those in primaries (the linear, non-linear, and multiple dummy variable analyses were conducted using data for all 33 general election candidates, because all of the general election races involved more than 2 candidates). We also conducted an analysis in which each voting place was weighted by one-half of the base-10 logarithm of the number of votes cast in the voting place (a method employed in [39]). To account for the source of heteroscedasticity in the vote shares, voting places were not weighted equally because candidates’ vote shares should be more variable in smaller voting places.

Finally, in races that had more than one non-major party candidate, the names of the non-major party candidates were not coded identically and instead were coded differently from one another, treating names in a vertical column as in different positions rather than the same position. For example, consider the race in which the Democratic candidate was listed in the first column, three non-major party candidates (referred to as other party candidate A, B, and C) were listed in the second column, with candidate A’s name listed at the top, candidate B’s name in the middle, and candidate C’s name at the bottom, and the Republican candidate was listed in the third column. In the analyses described above, the Democratic candidate was coded as being listed first, all three non-major party candidates (A, B, and C) were coded as being listed second, and the Republican candidate was coded as being listed third. In the alternative coding, the Democratic candidate was coded as being listed first, the three non-major party candidates—A, B, and C—were coded as being listed second, third, and fourth, respectively, and the Republican candidate was coded as being listed fifth.

### Moderators of the primacy effects

To gauge the impact of hypothesized moderators on the size of name order effects, we constructed a dataset in which each candidate provided a row of data and was therefore the unit of analysis. The parameters of OLS regressions were estimated predicting the size of the name order effect for that candidate (*Y*_*i*_) with positive values meaning primacy effects and negative values meaning recency effects, using (1) whether the race was a primary or a general election (*Primary*_i_), (2) whether the general election candidates were from major parties (*Major*_*i*_), (3) the publicity of the contest (*Publicity*_*i*_), (4) whether an incumbent was running for re-election (*Incumbent*_*i*_), and (5) the closeness of the contest (*Closeness*_*i*_). The subscripts *i* indicate the candidates. Five separate regressions were conducted as specified in the following estimation equations:

$$Y_{i}=a_{1}+{b_{1}\;Primay}_{i}+{b_{2}\;Major}_{i}+\varepsilon_{i}$$ (1)

$$Y_{i}=a_{1}+{b_{3}\;Publicity}_{i}+\varepsilon_{i}$$ (2)

$$Y_{i}=a_{1}+{b_{4}\;Incumbent}_{i}+\varepsilon_{i}$$ (3)

$$Y_{i}=a_{1}+{b_{5}\;Closeness}_{i}+\varepsilon_{i}$$ (4)

$$Y_{i}=a_{1}+{{b_{1}\;Primay}_{i}+{b_{2}\;Major}_{i}+{b_{3}\;Publicity}_{i}+b_{4}\;Incumbent}_{i}+b_{5}\;{Closeness}_{i}+\varepsilon_{i}$$ (5)

For all regressions with this dataset, standard errors were clustered by office, and we report robust and White-corrected standard errors due to the presence of heteroskedasticity. The dependent variable, the size of the candidate’s name order effect, is an estimate for each race, with associated uncertainty. One possible approach to taking this uncertainty into account would be to conduct a weighted least squares regression (WLS) using the inverse of the standard error of each name order effect as the weight. However, this approach usually yields inefficient parameter estimates and overconfidence in the estimates with smaller standard errors. It is therefore generally advised that OLS with White’s standard errors is the best approach and superior to WLS estimation [76, 77]. We therefore did the latter.

All analyses were performed using Stata version 15 [78].

## Results

### Equivalence checks of name rotation

Given that name order was assigned sequentially, we first assessed whether the ordering procedure produced equivalent groups of voting places that received different name orders (see S1 Text). The name order rotations in the 2000, 2002, and 2004 primaries yielded equivalent numbers of voters who saw each name order, as well as equivalent numbers of people registered to vote, registered as Democrats, registered as Republicans, or registered as neither a Democrat nor a Republican, and who filed their application to register to vote with the government on Election Day rather than before. The name rotations in the 2012 and 2016 general elections yielded equivalent numbers of voters who saw each name order, as well as equivalent numbers of people registered to vote, registered as Democrats, registered as Republicans, or registered as neither a Democrat nor a Republican (see S1 Text). The numbers of people who filed applications to register to vote with the government on Election Day were not available for the general elections. Furthermore, the name rotations in the 2012 and 2016 general elections yielded equivalent vote shares of Democratic and Republican presidential candidates in the previous general elections in 2008 and 2012, respectively (see S1 Text).

### Overall name order effects

#### Name order effects in the 2000, 2002, and 2004 primaries

**Fig 1:**
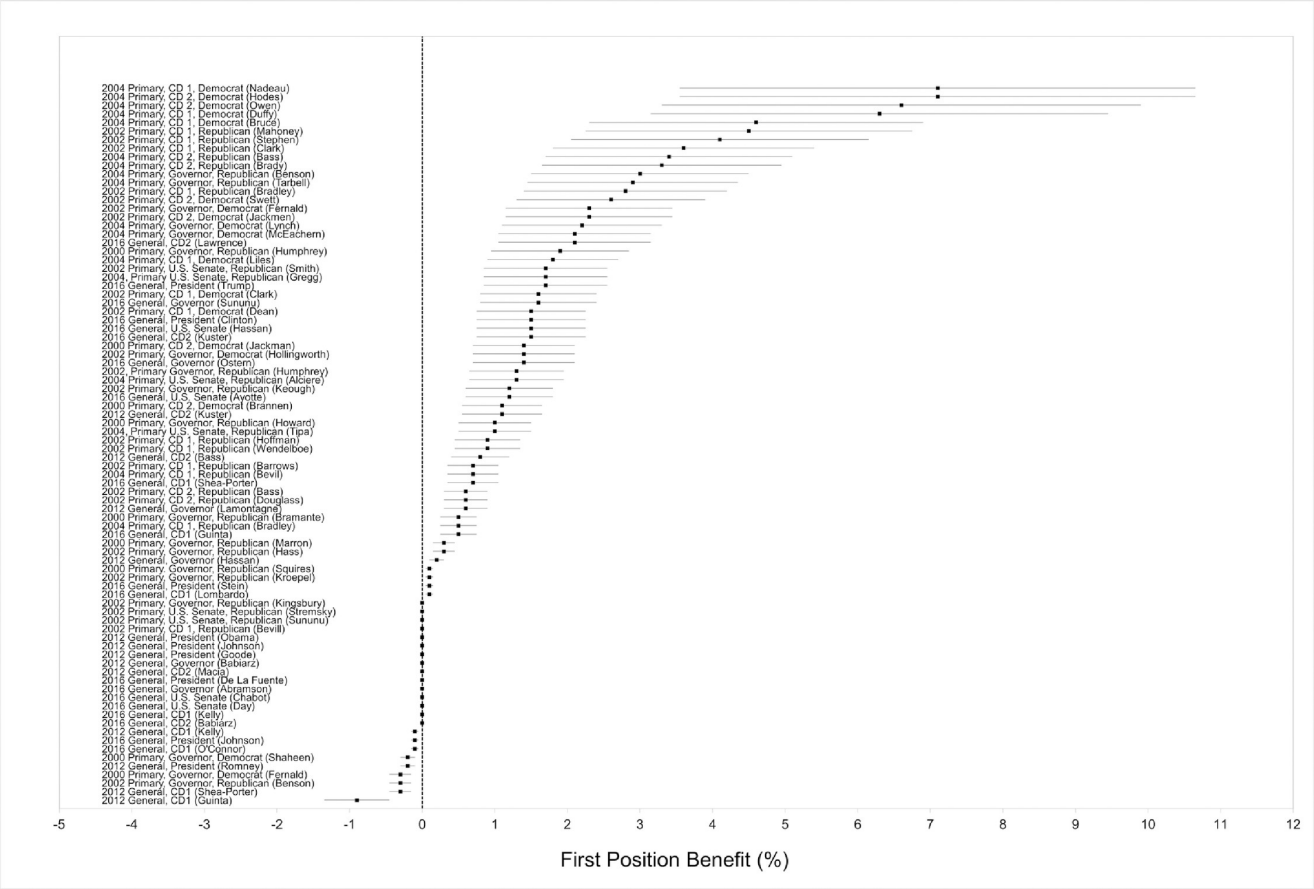
Name order effects in New Hampshire 2000, 2002, and 2004 primaries and 2012 and 2016 general elections.

**Fig 2:**
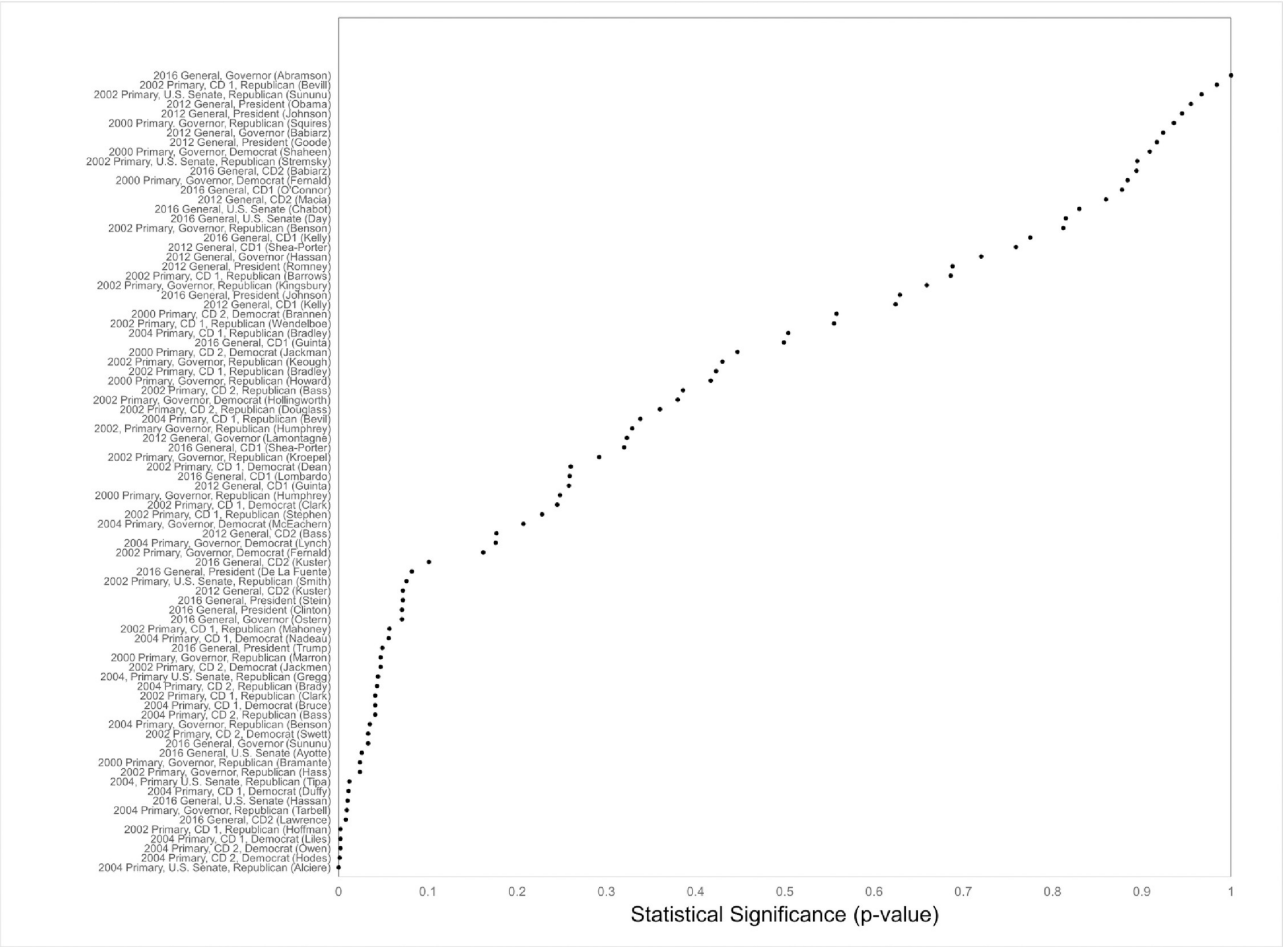
Statistical significance of name order effects in New Hampshire elections.

Of the 51 analyses of primaries in 2000, 2002, and 2004, 90% showed differences in the direction of primacy, which is significantly greater than the 50% that would be expected by chance alone (a sign test yielded p < .001; Fig 1 and column (3) in A1 Table in S1 Text with p-values in Fig 2). Among those differences in the direction of primacy, 41% were statistically significant, ranging from .3 to 7.1 percentage points and averaging 2.8 percentage points, and 7% were marginally statistically significant, ranging from 1.7 to 7.1 percentage points and averaging 4.4 percentage points.

#### Name order effects in the 2012 and 2016 general elections

Of the 33 candidates running in the 2012 and 2016 general elections, 67% manifested name order differences in the direction of primacy, again significantly greater than the 50% that would be expected by chance alone (a sign test yielded p < .04; Fig 1 and column (3) in A1 Table in S1 Text with p-values in Fig 2). Among the differences in the direction of primacy, 23% were statistically significant and 23% were marginally statistically significant, ranging from less than .01 to 2.1 percentage points and averaging 1.1 percentage points.

In 2016, both the Democratic and Republican presidential candidates received more votes when listed first than when listed later. Hillary Clinton manifested a marginally statistically significant primacy effect of 1.5 percentage points (p = .071), whereas Donald Trump manifested a statistically significant primacy effect of 1.7 percentage points (p = .049). These effects were not significantly different from one another (b = .002, p = .83), and the pooled name order effect combining the data from both candidates was 1.6 percentage points (p = .028). Marginally significant primacy effects were also observed for two of the three non-major party presidential candidates: .12 and .04 percentage points for Jill Stein and Roque “Rocky” De La Fuente, respectively (p = .072, p = .082).

#### Randomization inference

The randomization inference tests of statistical significance (column (4) in A1 Table in S1 Text) yielded comparable results to those from the OLS regressions (column (3) in A1 Table in S1 Text). Specifically, with regard to the primary analyses, 41% of the differences in the direction of primacy had p-values less than .05 using OLS, and the same 41% had p-values of less than .05 using randomization inference tests. Seven percent of the differences in the direction of primacy had OLS p-values between .05 and .10, and the same 7% had randomization inference p-values between .05 and .10. With regard to the general election analyses, 23% of the differences in the direction of primacy had p-values less than .05 using OLS, and the same 23% had p-values of less than .05 using randomization inferences tests. Twenty-three percent of the differences in the direction of primacy had OLS p-values between .05 and .10, and 18% had randomization p-values between .05 and .10.

#### Robustness checks for the 2000, 2002, and 2004 primaries

The linear, nonlinear, and multiple dummy variable specifications all yielded results similar to the single dummy variable specification (i.e., with the first position coded as 1 and the rest coded as 0; see S13, S15, S17, S19, S20, S22, S23, S27, S28, S30, S31, and SC1-SC5 Tables in S1 Text). Whereas 90% of the name order effects for candidates in races with more than two candidates were in the direction of primacy in the original name order analyses (using one dummy variable contrasting first with all other positions), 79% of the linear effects were in the direction of primacy. Forty-two percent of the primacy effects in the single dummy variable analysis were statistically significant, and 12% were marginally statistically significant. Thirty percent of the primacy effects in the linear analysis were statistically significant, and 9% were marginally statistically significant. Only 6 of the 29 analyses yielded statistically significant or marginally significant nonlinear effects.

When using multiple dummy variables to represent each position on the ballot separately, primacy effects also predominate. Across the dummy variable coefficient estimates, all of the statistically significant or marginally statistically significant effects are in the direction of primacy (see SC1-SC5 Tables in S1 Text). The appearance of primacy is more apparent when contrasting first position with positions toward the bottom of many-candidate races. Among the six analyses for the candidates in 3-candidate races, two of the coefficients for the first vs. second position dummy variables were statistically significant or marginally statistically significant, and three of the coefficients for the first vs. third position dummies were significant or marginally significant. Among the candidates running in the 4-candidate race, 1 of the coefficients for the first vs. second dummy was marginally significant, 4 of the coefficients for the first vs. third dummy were statistically significant, and 3 of the coefficients for the first vs. fourth dummy were statistically significant. For the 5-candidate race, none of the first vs. second coefficients were significant, 1 of the first vs. third coefficients was significant, 2 of the first vs. fourth coefficients were significant or marginally significant, and 1 of the first vs. fifth coefficients was significant. For the 6-candidate race, the number of significant or marginally significant coefficients for the first vs. second, third, fourth, fifth, and sixth dummy variables were: 0, 2, 2, 1, and 0, respectively. And for the 8-candidate race, the number of significant or marginally significant coefficients for the first vs. second, third, fourth, fifth, and sixth, seventh, and eighth dummy variables were: 1, 2, 2, 2, 2, 2, and 1, respectively.

#### Robustness checks for the 2012 and 2016 general elections

Weighted analyses with the original name order coding (first vs. all other positions) yielded similar findings to the unweighted results summarized above (see SC16, SC20, SC24, SC28, SC32, SC36, SC40, SC44, and SC48 Tables in S1 Text). Specifically, 67% of the name order effects were in the direction of primacy (the same percentage as the unweighted analyses); 18% of those primacy effects were statistically significant (23% of the unweighted primacy effects were statistically significant), and 14% were marginally statistically significant (23% of the unweighted primacy effects were marginally statistically significant).

The linear and nonlinear specifications (unweighted and weighted) also yielded results similar to the single dummy variable specification (see S27, S28, S30, S31, S33, S34, S36, S37, S39, S40, S42, S43, S45, S46, S48, S49, S51, S52, SC18, SC19, SC22, SC23, SC26, SC27, SC30, SC31, SC34, SC35, SC38, SC39, SC42, SC43, SC46, SC47, and SC50, SC51 Tables in S1 Text). Specifically, 91% of the unweighted linear effects were in the direction of primacy. And 88% of the weighted linear effects were in the direction of primacy. Of these primacy effects, 23% were statistically significant and 23% were marginally significant in unweighted analyses, and 28% were statistically significant and 21% were marginally significant in the weighted analyses. Ten of the 33 unweighted analyses yielded statistically significant or marginally significant nonlinear effects, whereas 11 of the 33 weighted analyses yielded statistically significant or marginally significant nonlinear effects.

With regard to the unweighted and weighted multiple dummy variable analyses, primacy effects were again the rule–only two of the unweighted significant effects were in the direction of recency, and none of the weighted significant effects were in the direction of recency; see SC8-SC15, SC17, SC21, SC25, SC29, SC33, SC37, SC41, SC45, and SC49 Tables in S1 Text). The effects were similar as the comparison moves down the ballot. For the unweighted analyses of the 15 candidates in the five 3-candidate races, the number of significant or marginally significant coefficients for the first vs. second as well as for the first vs. third dummy variables were 6 and 6, respectively. These numbers were 6 and 9, respectively, when weights were applied. For the eight analyses for the candidates in the two 4-candidate races, the number of significant or marginally significant unweighted coefficients for the first vs. second and first vs. third dummy variables were 2 and 1, respectively, and these numbers were 1 and 2, respectively, when weights were applied. For the analyses of candidates in the two 5-candidate races, the number of significant or marginally significant unweighted coefficients for the first vs. second and first vs. third dummy variables were 1 and 6, respectively. These numbers were 2 and 3, respectively, when weights were employed.

For the analyses of candidates in races that had more than one non-major party candidate, we re-coded the order variable to take into account the fact that multiple non-major candidates were all listed in the same column (one below the next) on the general election ballots (as described in the methods section; see SC52-SC59 Tables in S1 Text). These results documented predominance of primacy effects. Specifically, of the 18 analyses for candidates in races with more than one non-major party candidate, 16 (89%) were in the direction of primacy effects. Of those primacy effects, 1 was statistically significant (6%) and 6 were marginally statistically significant (38%). With this alternate coding, 4 of the 18 nonlinear effects were statistically significant or marginally statistically significant.

### Moderators of the primacy effects

When considered individually, all proposed moderators manifested statistically significant or marginally significant relations in the expected directions. For example, stronger primacy effects appeared in primaries and in general elections without candidates’ party affiliations on the ballot. Primary candidates and major-party candidates in general elections manifested primacy effects that were 1.89 and .84 percentage points, respectively, larger than did non-major-party candidates in general elections (-0.01 percentage points) (rows 1–2 column 1 in Table 1). Also, as expected, smaller primacy effects appeared for candidates in more publicized contests. The primacy effect for candidates in the most publicized contests was 2.42 percentage points smaller than for candidates in the least publicized contests (row (3) column (2) in Table 1). Likewise, primacy effects were 1.06 percentage points smaller in a contest with an incumbent than in a contest without one (row (4) column (3) in Table 1). Also, primacy effects were smaller in races with smaller margins of victory. The primacy effect for candidates in the contests with the largest margin of victory was 1.75 percentage points larger than that for candidates in the contests with the smallest margins of victory (row (5) column (4) in Table 1).

**Table 1:** Moderators of the primacy effects of 2000–2004 primary candidates and 2012–2016 general election candidates in New Hampshire.

	Primary/Major-party model	Publicity of the contest model	Incumbent model	Margin of victory model	Combined model
Predictor	(1)	(2)	(3)	(4)	(5)
Primary	1.891***				1.168+
	(0.419)				(0.678)
Major-party candidate in general elections	0.840**				0.733*
	(0.263)				(0.272)
Publicity of the contest		-2.423*			-1.416
		(1.013)			(1.131)
Contest with an incumbent			-1.063+		-0.791+
			(0.525)		(0.460)
Margin of victory				1.754*	0.182
				(0.683)	(1.249)
Constant	-0.007	2.411**	1.802***	0.620*	1.378
	(0.007)	(0.650)	(0.462)	(0.290)	(0.974)
R^2^	0.195	0.110	0.098	0.087	0.259
N	84	84	84	84	84

Notes. Cell entries are the unstandardized OLS regression coefficients and standard errors clustered at contests are in parentheses. The dependent variable is the size of the name order effect (shown in Fig 1, or the “Effect” column in A1 Table in S1 Text), calculated by subtracting the vote percentage for the candidate when he/she was listed first on the ballot compared to when listed later on the ballot. *Primary candidate* was coded 1 for candidates in primaries and 0 for candidates in general elections. *Major-party candidate in a general election* was coded 1 for Democratic or Republican candidates in general elections and 0 for all other candidates. *Publicity of the contest* is the square root of the total number of New Hampshire newspaper articles about the candidates in the contest, rescaled to range from 0 to 1. *Contest with an incumbent* was coded 1 for contests that had an incumbent as one of the candidates in the race and 0 for contests that had no incumbent running. *Margin of victory* is the square root of the difference in percentage points between the vote share of the winner (the candidate with the largest vote share) and the vote share of the candidate with the next largest vote share, rescaled to range from 0 to 1.

***p < .001

**p <. 01

*p < .05

+p < .10

When all of these moderators were analyzed simultaneously, the coefficients for most predictors were a bit smaller and generally maintained statistical significance or marginal significance (column (5) in Table 1). The coefficient for publicity, though sizable, became non-significant. And the coefficient for margin of victory shrank to near zero in the multivariate regression, suggesting that the effect is not robust. Variance inflation factors (VIF) indicated no problems with multicollinearity among the predictors: primary (3.24), major party candidate in general elections (1.75), publicity of the contest (1.55), contest with an incumbent (1.29), and closeness of the contest (2.37).

## Discussion

This paper contributes a number of new and important findings to the literature on name order effects that have not appeared in prior publications:

- First, for the first time, this research demonstrates candidate name order effects in the state of New Hampshire. Across the 2000, 2002, and 2004 Democratic and Republican primaries and the 2012 and 2016 general elections, differences in the direction of primacy were overwhelmingly prevalent. This is important because critics of existing evidence have routinely raised questions about the generalizability of findings from one state to another. Evidence such as this provides more assurance that name order effects appear to be a general phenomenon across American (and other nations’) elections, rather than being idiosyncratic to just a few states.
- Not only is this evidence important for the purpose of documenting generalizability of past findings to a new state, but it also documents generalizability to general elections with party column ballots. As noted above, the one prior study of party column ballots puzzlingly found no evidence of name (or column) order effects. The present evidence is therefore important, because it is the first evidence to document the expected primacy effects with such ballots.
- Third, the evidence reported here (and in S1 Text) is different from most past studies, because multiple different methods of analysis have been used, and all of them ended up supporting the same conclusions. Thus, this evidence contributes to debates in the literature about whether evidence of primacy effects comes and goes depending upon which analytic approach is taken. The present findings suggest no such fragility.
- Fourth, the present evidence advances knowledge about the moderators of candidate name order effects because it shows that, consistent with theory-based expectations, primacy effects were larger in primary elections than in partisan general elections. This is important because individual past studies have typically explored either primaries or general elections but not both. So those studies have not permitted comparisons of the effect sizes observed in both types of elections. The present paper reports tests of primaries and general elections happening contiguously in time in the same state, thereby offering a basis for concluding that name order effects were stronger in primaries.
- Fifth, the present evidence shows that primacy effects were greater among major-party candidates in general elections than among non-major-party candidates in general elections. And primacy effects were stronger in races with little publicity and without an incumbent running for re-election. This evidence complements the relatively small number of past studies that tested these studies and therefore reinforces confidence in their conclusions [34].
- Sixth, the evidence here regarding closeness of the race is important. Whereas Pasek et al. [34] found that name order effects in California were larger when the margin of victory in a race was not close, we did not see that relation here. This challenges that hypothesis and suggests that future exploration of it is merited.

These results are especially noteworthy given the electoral significance (for both candidates and voters) of New Hampshire, with regard to the presidential primaries. Although voters there are likely to be especially informed about candidates and engaged in politics, differences in the direction of primacy nonetheless appeared. For example, 90% of the New Hampshire primary races manifested differences in the direction of primacy, consistent with past findings that 89% of the primary races in New York and 100% of the primary races in Texas demonstrating primacy effects [39, 40]. Given New Hampshire’s role in winnowing candidates early on in the presidential primary season, these results are particularly striking.

Likewise, the observed primacy effects in New Hampshire’s 2016 presidential election (1.5 and 1.7 percentage points for Clinton and Trump) are nearly identical to some of the primacy effects documented in the few past studies that documented individual effects for major party presidential candidates. For example, in the 1976 California general election for President, a statistically significant primacy effect of 1.8% occurred for Jimmy Carter, and a marginally statistically significant primacy effect of 1.5% occurred for Gerald Ford. In the 2000 California general election for President, a marginally statistically significant primacy effect of 1.7% appeared for George W. Bush [34].

Lack of information is unlikely to explain the sizable name order effects in the 2016 presidential race in New Hampshire, because the nation was presumably extremely knowledgeable about the major party candidates in that year. A more likely explanation is ambivalence. In fact, 2016 marked the year in which the American public gave the major party candidates for president the most negative ratings in the history of polling (e.g., [79]). Voters loyal to Democratic candidates voiced reasons to hesitate about Secretary Clinton, and voters loyal to Republican candidates voiced reasons to hesitate about President Trump [80]. This ambivalence may have set the stage for unusually strong name order effects involving these candidates among highly informed and engaged voters, as in New Hampshire.

### The 2016 presidential election

The name order effects observed in the 2016 presidential election is especially interesting to think about, for two reasons. First, Secretary Clinton won that election by a fraction of a percent. This highlights the value of New Hampshire’s rotation system, because had either of the candidates been listed first on all ballots in the state, the present finding suggests that that candidate would have won the state.

It is also interesting to think about the possible implications for other states of the evidence of a sizable primacy effect in this highly publicized race in New Hampshire. President Trump won the electoral college vote by a margin of 77 votes, with very slim margins of popular vote victories in four states: Michigan (margin of 0.22%, 16 electoral votes), Wisconsin (margin of 0.76%, 10 electoral votes), Florida (margin of 1.20%, 29 electoral votes), and Pennsylvania (margin of .72%, 20 electoral votes). In this light, it is interesting to consider the procedure for ordering of names on ballots in those states and what might have happened if name order had been rotated.

President Trump was listed first on all ballots in three of the four states that he won by very small margins (Michigan, Wisconsin, and Florida), and in Pennsylvania, Secretary Clinton was always listed first. In Minnesota, where Secretary Clinton won 14 electoral votes by a very tight margin (margin of 1.52%, 10 electoral votes). President Trump was listed first on all ballots. If Michigan, Wisconsin, and Florida had rotated candidate name order across precincts, and if name order effects in those states were at least as large as those observed in the New Hampshire presidential race, President Trump would have lost in these three states, and 55 of his electoral votes would have shifted from him to Secretary Clinton, thereby flipping the nationwide election outcome (see S1 Text for detailed calculation).

Might the name order effect observed in New Hampshire have been as large or larger in Michigan, Wisconsin, and Florida? One reason to suspect this involves the level of education of voters in those states. Past research has shown that name order effects are greatest among the least educated voters. According to the 2016 exit polls, a smaller percentage of New Hampshire’s voters obtained any education beyond completing high school (13%) than in Michigan (20%), Wisconsin (20%) and Florida (18%). Likewise, more voters in New Hampshire had a college degree or more education (55%) than did so in Michigan (43%), Wisconsin (45%), and Florida (54%) [81]. Thus, at least according to educational attainment, candidate name order effects might have been larger in Michigan, Wisconsin, and Florida than in New Hampshire. So this simulation of election outcomes may yield a lower bound estimate of the size of name order effects, thus making reversal of the election outcome even more likely.

The 2016 presidential election is not the only one in recent history where the outcome may have changed if candidate name order had been rotated in certain states. In the 2000 presidential election between George W. Bush and Al Gore, Bush was listed ahead of Gore on every ballot in Florida and New Hampshire, where he won by just 0.009% (537 votes) and 1.27% (7,211 votes) over Gore, respectively. Had the 1.5% to 1.7% primacy effect observed in the 2016 presidential race in New Hampshire occurred in 2000 in Florida and New Hampshire and had candidate names been rotated, Gore would have carried both states. If Gore had won only one of those two states, he would have been elected President instead of Bush.

## Conclusion

The present findings contribute to the existing literature on the electoral advantage of being listed first, generalizing the past research in a limited set of geographic areas to a state where the name order effects might be weak or entirely absent. Primacy effects appeared even in a highly politically engaged and informed state using party column ballots, which increase the salience of party cues at the moment of voting. Because of the importance of races across the nation that have been won by slim margins in states that listed the winners first on all ballots, the findings outlined here encourage election officials and legislators to consider enhancing electoral fairness by rotating name order rather than using a single name order.

## Supporting information

S1 Text (DOCX)
