# Supplementary material for: Candidate name order effects in New Hampshire: Evidence from primaries and from general elections with party column ballots
Source: PLoS One. 2021 Mar 16;16(3):e0248049. doi: 10.1371/journal.pone.0248049 (PMC7963059; doi:10.1371/journal.pone.0248049)
Supplement: S1 Text — (DOCX) [file pone.0248049.s001.docx]

S1 Text

**Candidate Name Order Effects in New Hampshire:**

**Evidence from Primaries and from General Elections with Party Column Ballots**

Bo MacInnis, Joanne Miller, Jon A. Krosnick^*^, Clifton Below, Miriam Lindner

**Overview**

This online supplement provides the following:

1. Rotation Procedures
2. Results of Name Order Effects
3. Robustness Checks of Name Order Effects
4. Discussion on the 2016 Presidential Election
5. Figures

Figure S1. Reproduced Table 1 in Koppell and Steen (2004)

Figure S2. Reproduced Table 2 in Koppell and Steen (2004)

Figure S3. Reproduced Table 4 in Grant (2017)

Figure S4. 2012 General Election Ballot of Alstead, New Hampshire

Figure S5. 2016 General Election Ballot of Acworth, New Hampshire

1. Tables

Table A1. Name Order Effects of 2000-2004 Primary Candidates and 2012-2016 General Election Candidates in New Hampshire

Table S1. Name Order Effects in the Two-Candidate 2000 Democratic Primary for Governor in New Hampshire: First Versus Last

Table S2. Name Order Effects in the Two-Candidate 2000 Democratic Primary for 2nd Congressional District in New Hampshire: First Versus Last

Table S3. Name Order Effects in the Two-Candidate 2002 Democratic Primary for Governor in New Hampshire: First Versus Last

Table S4. Name Order Effects in the Two-Candidate 2002 Democratic Primary for 1st Congressional District in New Hampshire: First Versus Last

Table S5. Name Order Effects in the Two-Candidate 2002 Democratic Primary for 2nd Congressional District in New Hampshire: First Versus Last

Table S6. Name Order Effects in the Two-Candidate 2002 Republican Primary for 2nd Congressional District in New Hampshire: First Versus Last

Table S7. Name Order Effects in the Two-Candidate 2004 Democratic Primary for Governor in New Hampshire: First Versus Last

Table S8. Name Order Effects in the Two-Candidate 2004 Republican Primary for Governor in New Hampshire: First Versus Last

Table S9. Name Order Effects in the Two-Candidate 2004 Republican Primary for 1st Congressional District in New Hampshire: First Versus Last

Table S10. Name Order Effects in the Two-Candidate 2004 Democratic Primary for 2nd Congressional District in New Hampshire: First Versus Last

Table S11. Name Order Effects in the Two-Candidate 2004 Republican Primary for 2nd Congressional District in New Hampshire: First Versus Last

Table S12. Name Order Effects in the Three-Candidate 2002 Republican Primary for US Senate in New Hampshire: First Versus Second/Third

Table S13. Name Order Effects in the Three-Candidate 2002 Republican Primary for US Senate in New Hampshire: Name Order and Name Order Squared

Table S14. Name Order Effects in the Three-Candidate 2004 Republican Primary for US Senate in New Hampshire: First Versus Second/Third

Table S15. Name Order Effects in the Three-Candidate 2004 Republican Primary for US Senate in New Hampshire: Name Order and Name Order Squared

Table S16. Name Order Effects in the Five-Candidate 2000 Republican Primary for Governor in New Hampshire: First Versus Later

Table S17. Name Order Effects in the Five-Candidate 2000 Republican Primary for Governor in New Hampshire: Name Order and Name Order Squared

Table S18. Name Order Effects in the Six-Candidate 2002 Republican Primary for Governor in New Hampshire: First Versus Later

Table S19. Name Order Effects in the Six-Candidate 2002 Republican Primary for Governor in New Hampshire: Name Order

Table S20. Name Order Effects in the Six-Candidate 2002 Republican Primary for Governor in New Hampshire: Name Order and Name Order Squared

Table S21. Name Order Effects in the Eight-Candidate 2002 Republican Primary for 1st Congressional District in New Hampshire: First Versus Later

Table S22. Name Order Effects in the Eight-Candidate 2002 Republican Primary for 1st Congressional District in New Hampshire: Name Order

Table S23. Name Order Effects in the Eight-Candidate 2002 Republican Primary for 1st Congressional District in New Hampshire: Name Order and Name Order Squared

Table S24. Name Order Effects in the Four-Candidate 2004 Democratic Primary for 1st Congressional District in New Hampshire: First Versus Later

Table S25. Name Order Effects in the Four-Candidate 2004 Democratic Primary for 1st Congressional District in New Hampshire: First Versus Later

Table S26. Name Order Effects in the 2012 Presidential Election in New Hampshire: First Versus Later

Table S27. Name Order Effects in the 2012 Presidential Election in New Hampshire: Name Order

Table S28. Name Order Effects in the 2012 Presidential Election in New Hampshire: Name Order and Name Order Squared

Table S29. Name Order Effects in the 2012 Gubernatorial Election in New Hampshire: First Versus Later

Table S30. Name Order Effects in the 2012 Gubernatorial Election in New Hampshire: Name Order

Table S31. Name Order Effects in the 2012 Gubernatorial Election in New Hampshire: Name Order and Name Order Squared

Table S32. Name Order Effects in the 2012 Congressional District 1 Election in New Hampshire: First Versus Later

Table S33. Name Order Effects in the 2012 Congressional District 1 Election in New Hampshire: Name Order

Table S34. Name Order Effects in the 2012 Congressional District 1 Election in New Hampshire: Name Order and Name Order Squared

Table S35. Name Order Effects in the 2012 Congressional District 2 Election in New Hampshire: First Versus Later

Table S36. Name Order Effects in the 2012 Congressional District 2 Election in New Hampshire: Name Order

Table S37. Name Order Effects in the 2012 Congressional District 2 Election in New Hampshire: Name Order and Name Order Squared

Table S38. Name Order Effects in the 2016 Presidential Election in New Hampshire: First Versus Later

Table S39. Name Order Effects in the 2016 Presidential Election in New Hampshire: Name Order

Table S40. Name Order Effects in the 2016 Presidential Election in New Hampshire: Name Order and Name Order Squared

Table S41. Name Order Effects in the 2016 U.S. Senate Election in New Hampshire: First Versus Later

Table S42. Name Order Effects in the 2016 U.S. Senate Election in New Hampshire: Name Order

Table S43. Name Order Effects in the 2016 U.S. Senate Election in New Hampshire: Name Order and Name Order Squared

Table S44. Name Order Effects in the 2016 Gubernatorial Election in New Hampshire: First Versus Later

Table S45. Name Order Effects in the 2016 Gubernatorial Election in New Hampshire: Name Order

Table S46. Name Order Effects in the 2016 Gubernatorial Election in New Hampshire: Name Order and Name Order Squared

Table S47. Name Order Effects in the 2016 Congressional District 1 Election in New Hampshire: First Versus Later

Table S48. Name Order Effects in the 2016 Congressional District 1 Election in New Hampshire: Name Order

Table S49. Name Order Effects in the 2016 Congressional District 1 Election in New Hampshire: Name Order and Name Order Squared

Table S50. Name Order Effects in the 2016 Congressional District 2 Election in New Hampshire: First Versus Later

Table S51. Name Order Effects in the 2016 Congressional District 2 Election in New Hampshire: Name Order

Table S52. Name Order Effects in the 2016 Congressional District 2 Election in New Hampshire: Name Order and Name Order Squared

Appendix A: New Hampshire Laws for Ballot Name Order

Appendix B: Equivalence Checks of Name Rotation Tables

Table SB1. T-Tests Predicting Total Votes in the Race with Candidate Name Order for Two-Candidate Races in 2000, 2002, and 2004 Primaries in New Hampshire

Table SB2. ANOVAs Predicting Total Votes in the Race with Candidate Name Order for 3+Candidate Races in 2000, 2002, and 2004 Primaries in New Hampshire

Table SB3. T-Tests Predicting Registered Votes in the Race with Candidate Name Order for Two-Candidate Races in 2000, 2002, and 2004 Primaries in New Hampshire

Table SB4. ANOVAs Predicting Registered Votes in the Race with Candidate Name Order for 3+Candidate Races in 2000, 2002, and 2004 Primaries in New Hampshire

Table SB5. T-Tests Predicting Registered Democrats or Republicans (Depending on Primary) in the Race with Candidate Name Order for Two-Candidate Races in 2000, 2002, and 2004 Primaries in New Hampshire

Table SB6. ANOVAs Predicting Registered Democrats or Republicans (Depending on Primary) in the Race with Candidate Name Order for 3+Candidate Races in 2000, 2002, and 2004 Primaries in New Hampshire

Table SB7. T-Tests Predicting Undeclared Registered Voters in the Race with Candidate Name Order for Two-Candidate Races in 2000, 2002, and 2004 Primaries in New Hampshire

Table SB8. ANOVAs Predicting Undeclared Registered Voters in the Race with Candidate Name Order for 3+Candidate Races in 2000, 2002, and 2004 Primaries in New Hampshire

Table SB9. T-Tests Predicting Total Number of People who Registered on Election Day in the Race with Candidate Name Order for Two-Candidate Races in 2000, 2002, and 2004 Primaries in New Hampshire

Table SB10. ANOVAs Predicting Total Number of People who Registered on Election Day with Candidate Name Order for 3+Candidate Races in 2000, 2002, and 2004 Primaries in New Hampshire

Table SB11. ANOVAs Predicting Total Votes with Candidate Name Order in 2012 and 2016 General Election in New Hampshire

Table SB12. ANOVAs Predicting Registered Voters with Candidate Name Order in 2012 and 2016 General Election in New Hampshire

Table SB13. ANOVAs Predicting Registered Democratic Voters with Candidate Name Order in 2012 and 2016 General Election in New Hampshire

Table SB14. ANOVAs Predicting Registered Republican Voters with Candidate Name Order in 2012 and 2016 General Election in New Hampshire

Table SB15. ANOVAs Predicting Registered Undeclared Voters with Candidate Name Order in 2012 and 2016 General Election in New Hampshire

Table SB16. ANOVAs Predicting Major Party Presidential Candidates’ Vote Shares in 2008 (and 2012) General Election with Candidate Name Order in 2012 (and 2016) General Election in New Hampshire

Appendix C: Robustness Checks Tables

Table SC1. Name Order Effects in the Three-Candidate 2002 Republican Primary for US Senate in New Hampshire: Second/Third Versus First

Table SC2. Name Order Effects in the Three-Candidate 2004 Republican Primary for US Senate in New Hampshire: Second/Third Versus First

Table SC3. Name Order Effects in the Five-Candidate 2000 Republican Primary for Governor in New Hampshire: Second/Third/Fourth/Fifth Versus First

Table SC4. Name Order Effects in the Six-Candidate 2002 Republican Primary for Governor in New Hampshire: Second/Third/Fourth/Fifth/Sixth Versus First

Table SC5. Name Order Effects in the Eight-Candidate 2002 Republican Primary for 2nd Congressional District in New Hampshire: Second/Third/Fourth/Fifth/Sixth/Seven/Eighth Versus First

Table SC6. Name Order Effects in the Four-Candidate 2004 Democratic Primary for 1st Congressional District in New Hampshire: Second/Third/Fourth Versus First

Table SC7. Name Order Effects in the 2012 Presidential Election in New Hampshire: Second/Third Versus First

Table SC8. Name Order Effects in the 2012 Gubernatorial Election in New Hampshire: Second/Third Versus First

Table SC9. Name Order Effects in the 2012 Congressional District 1Eelection in New Hampshire: Second/Third Versus First

Table SC10. Name Order Effects in the 2012 Congressional District 2 Election in New Hampshire: Second/Third Versus First

Table SC11. Name Order Effects in the 2016 Presidential Election in New Hampshire: Second/Third Versus First

Table SC12. Name Order Effects in the 2016 U.S. Senate Election in New Hampshire: Second/Third Versus First

Table SC13. Name Order Effects in the 2016 Gubernatorial Election in New Hampshire: Second/Third Versus First

Table SC14. Name Order Effects in the 2016 Congressional District 1 Election in New Hampshire: Second/Third Versus First

Table SC15. Name Order Effects in the 2016 Congressional District 2 Election in New Hampshire: Second/Third Versus First

Table SC16. Name Order Effects in the 2012 Presidential Election in New Hampshire: First Versus Later (With Weights)

Table SC17. Name Order Effects in the 2012 Presidential Election in New Hampshire: Second/Third Versus First (With Weights)

Table SC18. Name Order Effects in the 2012 Presidential Election in New Hampshire: Name Order (With Weights)

Table SC19. Name Order Effects in the 2012 Presidential Election in New Hampshire: Name Order and Name Order Squared (With Weights)

Table SC20. Name Order Effects in the 2012 Gubernatorial Election in New Hampshire: First Versus Later (With Weights)

Table SC21. Name Order Effects in the 2012 Gubernatorial Election in New Hampshire: Second/Third Versus First (With Weights)

Table SC22. Name Order Effects in the 2012 Gubernatorial Election in New Hampshire: Name Order (With Weights)

Table SC23. Name Order Effects in the 2012 Gubernatorial Election in New Hampshire: Name Order and Name Order Squared (With Weights)

Table SC24. Name Order Effects in the 2012 Congressional District 1 Election in New Hampshire: First Versus Later (With Weights)

Table SC25. Name Order Effects in the 2012 Congressional District 1 Election in New Hampshire: Second/Third Versus First (With Weights)

Table SC26. Name Order Effects in the 2012 Congressional District 1 Election in New Hampshire: Name Order (With Weights)

Table SC27. Name Order Effects in the 2012 Congressional District 1 Election in New Hampshire: Name Order and Name Order Squared (With Weights)

Table SC28. Name Order Effects in the 2012 Congressional District 2 Election in New Hampshire: First Versus Later (With Weights)

Table SC29. Name Order Effects in the 2012 Congressional District 2 Election in New Hampshire: Second/Third Versus First (With Weights)

Table SC30. Name Order Effects in the 2012 Congressional District 2 Election in New Hampshire: Name Order (With Weights)

Table SC31. Name Order Effects in the 2012 Congressional District 2 Election in New Hampshire: Name Order and Name Order Squared (With Weights)

Table SC32. Name Order Effects in the 2016 Presidential Election in New Hampshire: First Versus Later (With Weights)

Table SC33. Name Order Effects in the 2016 Presidential Election in New Hampshire: Second/Third Versus First (With Weights)

Table SC34. Name Order Effects in the 2016 Presidential Election in New Hampshire: Name Order (With Weights)

Table SC35. Name Order Effects in the 2016 Presidential Election in New Hampshire: Name Order and Name Order Squared (With Weights)

Table SC36. Name Order Effects in the 2016 U.S. Senate Election in New Hampshire: First Versus Later (With Weights)

Table SC37. Name Order Effects in the 2016 U.S. Senate Election in New Hampshire: Second/Third Versus First (With Weights)

Table SC38. Name Order Effects in the 2016 U.S. Senate Election in New Hampshire: Name Order (With Weights)

Table SC39. Name Order Effects in the 2016 U.S. Senate Election in New Hampshire: Name Order and Name Order Squared (With Weights)

Table SC40. Name Order Effects in the 2016 Gubernatorial Election in New Hampshire: First Versus Later (With Weights)

Table SC41. Name Order Effects in the 2016 Gubernatorial Election in New Hampshire: Second/Third Versus First (With Weights)

Table SC42. Name Order Effects in the 2016 Gubernatorial Election in New Hampshire: Name Order (With Weights)

Table SC43. Name Order Effects in the 2016 Gubernatorial Election in New Hampshire: Name Order and Name Order Squared (With Weights)

Table SC44. Name Order Effects in the 2016 Congressional District 1 Election in New Hampshire: First Versus Later (With Weights)

Table SC45. Name Order Effects in the 2016 Congressional District 1 Election in New Hampshire: Second/Third Versus First (With Weights)

Table SC46. Name Order Effects in the 2016 Congressional District 1 Election in New Hampshire: Name Order (With Weights)

Table SC47. Name Order Effects in the 2016 Congressional District 1 Election in New Hampshire: Name Order and Name Order Squared (With Weights)

Table SC48. Name Order Effects in the 2016 Congressional District 2 Election in New Hampshire: First Versus Later (With Weights)

Table SC49. Name Order Effects in the 2016 Congressional District 2 Election in New Hampshire: Second/Third Versus First (With Weights)

Table SC50. Name Order Effects in the 2016 Congressional District 2 Election in New Hampshire: Name Order (With Weights)

Table SC51. Name Order Effects in the 2016 Congressional District 2 Election in New Hampshire: Name Order and Name Order Squared (With Weights)

Table SC52. Name Order Effects in the 2012 Presidential Election in New Hampshire: Name Order with Alternative Name Order Coding

Table SC53. Name Order Effects in the 2012 Presidential Election in New Hampshire: Name Order and Name Order Squared with Alternative Name Order Coding

Table SC54. Name Order Effects in the 2016 Presidential Election in New Hampshire: Name Order with Alternative Name Order Coding

Table SC55. Name Order Effects in the 2016 Presidential Election in New Hampshire: Name Order and Name Order Squared with Alternative Name Order Coding

Table SC56. Name Order Effects in the 2016 U.S. Senate Election in New Hampshire: Name Order with Alternative Name Order Coding

Table SC57. Name Order Effects in the 2016 U.S. Senate Election in New Hampshire: Name Order and Name Order Squared with Alternative Name Order Coding

Table SC58. Name Order Effects in the 2016 Congressional District 1 Election in New Hampshire: Name Order with Alternative Name Order Coding

Table SC59. Name Order Effects in the 2016 Congressional District 1 Election in New Hampshire: Name Order and Name Order Squared with Alternative Name Order Coding

1. **Rotation Procedures**

**Rotation procedures in 2000, 2002, and 2004 primaries.** Rotations for the 2000, 2002, and 2004 primary elections were created by a computer program that sorted lists of voting places by the number of voters registered with each party, from largest to smallest, and then assigned rotations between candidates of the same party from largest to smallest voting places. Adjustments were made so that the total number of registered voters of the same party in all voting places where the candidate's name appeared in each position on the ballot was approximately equal (+/- 5 typically). Karen Ladd at the New Hampshire Secretary of State's office indicated that she manually went through the rotation order and made a few swaps of nearly equal-sized city wards and towns to better balance the rotation of each city’s wards between the candidates and to prevent all or nearly all wards in a city from being assigned to just one candidate for a given rotation. These manual adjustments were usually infrequent in number (e.g., 0 to 5 per race in 2004) and were more likely for multi-candidate races, especially in 2002, since the town of Londonderry was apparently inadvertently excluded from the computer-generated lists in the First Congressional District (CD-1) and had to be added in, triggering a number of swaps. Voting places that had fewer than 10 registered voters of a particular party (primarily unincorporated places) were apparently assigned 10 as an assumed minimum.

**Rotation procedures in general elections.** Legislation was enacted in 2010 that specified a generic party column rotation scheme for the 2010 election based on votes cast in the 2008 general election and set forth the procedure described below, based on census population, for subsequent elections. The rotations take place across the 323 towns, city wards, and unincorporated places, constituting what are called the “voting places” in the state. Candidate names in general elections starting in 2012 were rotated by party column under the statute NH RSA 656:5 ([www.gencourt.state.nh.us/rsa/html/LXIII/656/656-5.htm](http://www.gencourt.state.nh.us/rsa/html/LXIII/656/656-5.htm)):

I. The names of all candidates nominated in accordance with the election laws shall be arranged upon the state general election ballot in successive party columns. Each separate column shall contain the names of the candidates of one party; except that, if only a part of a full list of candidates is nominated by a political party, 2 or more such lists may be arranged whenever practicable in the same column.

II. The position of party columns shall be rotated on the ballots used so that each party column shall appear thereon, to the extent practicable, an approximately equal number of times in the first, last, and each intermediate column position across the state, without requiring more than one unique column order or ballot format for each town, ward, or unincorporated place. Starting with the general election for 2012 and following each new apportionment of representative districts, but before the close of the period during which a person may accept the nomination of a party committee pursuant to RSA 655:32, the secretary of state shall develop as many generic column rotation plans for use in general elections as he or she might reasonably expect to be needed for different possible numbers of party columns on the general election ballot. If the number of party columns expected on the general election ballot changes such that one or more additional generic column rotation plans are needed, the secretary of state shall, from time to time, prepare such additional plans as are needed for any general election.

III. The generic column rotation plans shall be based on a reasonably balanced rotation of party columns within and across all non-floterial state representative districts, those being the smallest representative districts to which each voting place is apportioned pursuant to part I, article 11 of the New Hampshire constitution. Consideration shall also be given to reasonably minimize any obvious, substantial, and avoidable imbalances in column rotation within senate districts. The average deviation from equal rotation for the first party column position, measured across the state as a whole and based on population according to the last decennial federal census, shall be as close to 0 percent as is practicable but in no event greater than 1 percent. Once generic column rotation plans are established the secretary of state shall publish such plans to the department's website.

IV. Immediately following the close of the period during which a person may accept the nomination of a party committee pursuant to RSA 655:32, the secretary of state or designee shall publicly select by lot the actual party columns to be positioned according to the generic column rotation plan established pursuant to paragraphs II and III. No party shall be assigned the same generic party column designation for 2 consecutive general elections.”

The procedure described in RSA 656:5 has been implemented by first associating each of the 323 voting places in the state with the unique non-floterial state representative and Senate district of which they are part. Every town, city ward, and unincorporated place, or voting place, is made part of one and only one non-floterial representative district that each has 1 or more seats based on population. New Hampshire also has floterial state representative districts that “float” over 2 or more non-floterial districts, like at-large districts. The New Hampshire House of Representatives, with 400 seats, is the largest state legislative body in the nation, whereas the Senate, with only 24 seats, is one of the smallest.

The list of voting places is then sorted first into the non-floterial state representative districts that they comprise. These state representative districts are then sorted by the number of voting places in each district, from largest to smallest. Within each such state representative district, voting places are also sorted from largest to smallest. A basic generic rotation scheme is next applied to the largest voting place in each representative district by assigning which generic party gets the first position in sequential rotating order going down the list of districts.

Then in all the districts that have more than one voting place, a reversing rotation scheme is applied starting with the initial rotation applied to largest voting place in each district to reasonably balance rotation within each representative district with more than one voting place. After each generic party is assigned the next largest voting place for first column position in sequential order, the last party assigned a first position, which is to the smallest voting place thus far assigned, is assigned the next voting place or places for first position until its allocation exceeds that of the generic party with next smallest allocation. Each time a generic party moves up from last in allocation of potential ballots where it is placed in first position, the rotation moves to the new generic party in last place for allocation of first position until it moves up from last. This form of manual tweaking is not specified in the law but is effectively required by the law to reasonably balance rotation of party columns within and across all non-floterial state representative districts.

The assignment of additional column positions after the first position also involves systematically reversing the order of additional position assignments. So for 3 party column ballots, where party 1 is in first position, the first time the party order for 2^nd^ and 3^rd^ position is assigned the party order would be 1,2,3; the second time it would be 1,3,2, and then back to 1,2,3, then 1,3,2 and so forth. The first time in the list that party 3 has the first position the party order for each column position would be 3,1,2 and then the second time 3,2,1, then back to 3,1,2, then 3,2,1 and so forth. This method systematically rotates and mixes up the generic party order for 2^nd^ and 3^rd^ column positions.

That list of all voting places with a tentative rotation scheme that optimizes for rotation within representative districts is then sorted by state Senate districts, maintaining a sort from largest to smallest voting places within each Senate district. The equality of rotation within each Senate district is then calculated and reviewed as an absolute and percentage deviation from perfectly balanced rotation for the first position. A manual and discretionary tweaking of rotations is then undertaken to “reasonably minimize any obvious, substantial, and avoidable imbalances in column rotation within Senate districts.” It becomes a challenge of reducing large and avoidable deviations within Senate and House districts while simultaneously reducing the overall statewide deviation from equal rotation with the absolute requirement that it not exceed 1%.

Although there is a discretionary and subjective aspect to this manual tweaking to minimize deviations from equal rotation based on population (from 2012 forward), there is no apparent way for any person involved in such tweaking to advantage one party over another, because the public random drawing in which actual political parties are assigned to each generic position for each general election does not occur until after the generic rotation scheme is established and published. This random draw of which party gets which generic position for the general election also occurs after the primary election, after all independent candidate filings, and after parties can file candidates for open seats where there is no candidate for their party after the primary, so candidates cannot know where they will appear on the ballot until after being fully committed to party or independent affiliation candidacies. A grouping of independent and minor party candidates typically shares a column as if they are a party but could be further split based on the number of such candidates for any one office. A column just for write-in candidates always appears in the last position.

1. **Equivalence Checks of Name Rotation**

We first conducted series of analyses to determine whether name rotation was effectively “random” (meaning that the orders were not significantly different from one another on a variety of town-specific variables). The name rotation in the 2000, 2002, and 2004 primaries yielded equivalent numbers of actual voters who saw each name order (all p’s>.21 in two-candidate races; see Table SB1; p’s>.75 in greater-than two-candidate races; see Table SB2). The fact that none of these p-values is <.05 means that there was not a statistically significant difference in the number of voters who received each name order. The same conclusion was reached with regard to the number of registered voters in the towns assigned to different name orders (all p’s>.42 in two-candidate races; see Table SB3; p’s>.85 in greater than two-candidate races; see Table SB4), registered Democrats or Republicans (depending on the primary; all p’s>.37 in two-candidate races; see Table SB5; all p’s>.91 in the greater than two-candidate races; see Table SB6), undeclared registered voters (all p’s>.23 in two-candidate races; see Table SB7; all p’s>.67 in greater than two-candidate races; see Table SB8), and number of people who registered on Election Day (all p’s>.29 in two-candidate races; see Table SB9; all p’s>.91 in the greater than three-candidate races; see Table SB10).

The name rotation in the 2012 and 2016 general election yielded equivalent numbers of actual voters who saw each party’s candidate in each name order (all p’s>.15 in the 2012 election and all p’s>.20 in the 2016 election; see Table SB11), and the same conclusion was reached with regard to registered voters (all the p’s>.43 in the 2012 election; p’s>.44 in the 2016 election; see Table SB12). Likewise, the same conclusion was reached with regard to registered Democratic voters (all the p’s>.43 in the 2012 election; p’s>.44 in the 2016 election; see Table SB13), registered Republican voters (all the p’s>.58 in the 2012 election; p’s>.26 in the 2016 election; see Table SB14), or registered undeclared (neither Democratic nor Republican) voters (all the p’s>.52 in the 2012 election; p’s>.44 in the 2016 election; see Table SB15). Furthermore, the name rotation in the 2012 and 2016 general election yielded equivalent vote shares of Democratic and Republican Presidential candidates in the 2008 and 2012 general elections, respectively (all the p’s>.16 in the 2012 election; p’s>.27 in the 2016 election; see Table SB16), indicating that the ballot order in towns is not correlated with the electoral strengths of certain political party or candidate.

1. **Detailed Results for the Model Specification Described in the Manuscript**

**Name order effects in the two-candidate races in the 2000-2004 primaries.** Statistically significant primacy effects were evident in four of the 11 two-candidate races as follows:

- 2002 Democratic primary for 2nd Congressional District (Table S5)
- 2004 Republican primary for Governor (Table S8)
- 2004 Democratic primary for 2nd Congressional District (Table S10)
- 2004 Republican primary for 2nd Congressional District (Table S11)

In the 2002 Democratic primary for the 2nd Congressional District, both candidates received more votes when listed first than when listed second; two percentage points more and three percentage points more for Jackman and Swett, respectively (p’s<.05; row 1 and columns 1-2 in Table S5). In the 2004 Republican primary for Governor, both candidates, Benson and Tarbell, received three percentage points more votes when listed first than when listed second (p<.05, p<.01; row 1 and columns 1-2 in Table S8). In the 2004 Democratic primary for the 2nd Congressional District, both candidates, Hodes and Owen, received seven percentage points more votes when listed first than when listed second (p<.001, p<.01; row 1 and columns 1-2 in Table S10).

In the 2004 Republican primary for the 2nd Congressional District, both candidates, Bass and Brady, received three percentage points more votes when listed first than when listed later (p’s<.05; row 1 and columns 1-2 in Table S11). There was no evidence of name order effects in the rest of the two-candidate races, including the 2000 Democratic primary for Governor (Table S1), the 2000 Democratic primary for 2nd Congressional District (Table S2), the 2002 Democratic primary for Governor (Table S3), the 2002 Democratic primary for 1st Congressional District (Table S4), the 2002 Republican primary for 2nd Congressional District (Table S6), the 2004 Democratic primary for Governor (Table S7), and the 2004 Republican primary for 1st Congressional District (Table S9).

**Name order effects in the three-candidate races in the 2000-2004 primaries.** In the 2002 Republican primary for the U.S. Senate, one candidate, Smith, received two percentage points more votes when listed first than when listed later (p<.10; row 1 and column 1 in Table S12), and the other two candidates, Stremsky and Sununu, received the same percentage of votes, whether listed first or listed later (row 1 and columns 2-3 in Table S12).

In the 2004 Republican primary for the U.S. Senate, all three candidates received more votes when listed first than when listed later: one percentage point more for Aciere, two percentage points more for Gregg, and one percentage point more for Tipa (p<.001, p<.05, p<.05; row 1 in Table S14).

**Name order effects in the greater than three-candidate races in the 2000-2004 primaries.** Statistically significant primacy effects were evident in all of the greater than two-candidate races we analyzed: the 2004 Democratic primary for Congressional District 1 (four candidates), the 2000 Republican primary for Governor (five candidates), the 2002 Republican primary for Governor (six candidates), and the 2002 Republican primary for Congressional District 1 (eight candidates).

In the five-candidate 2000 Republican primary for Governor, primacy effects were evident for two candidates: Bramante and Marron received one and three-tenths percentage points more votes respectively when listed first than when listed later (p’s<.05; row 1 and columns 1 and 4 in Table S16). Primacy effects were not evident for the other three candidates (row 1 and columns 2, 3, and 6 in Table S16).

In the six-candidate 2002 Republican primary for Governor, a statistically significant primacy effect was evident for one candidate: Haas, but not for any of the other candidates (row 1 and columns 1 and 3-6 in Table S18). Haas received three-tenths percentage point more votes when listed first than when listed later (p<.05; row and column 2 in Table S18).

In the eight-candidate 2002 Republican primary for the 2nd Congressional District, primacy effect were manifest for three candidates: Clark, Hoffman, and Mahoney received four, one, and five percentage points more votes respectively when listed first (p<.05, p<.01, p<.10; row 1 and columns 4-6 in Table S21).

In the four-candidate 2004 Democratic primary for the 1st Congressional District, primacy effect occurred for all the candidates: Bruce, Duffey, Liles, and Nadeau received five, six, two, and seven percentage points more votes respectively when listed first (p<.05, p<.05,p<.01,p<.10; row 1 in Table S24).

**Name order effects in the 2012 general election.**

***The 2012 U.S. presidential election***. In the 2012 election, presidential candidates Barack Obama, Mitt Romney, Gary Johnson, and Virgil Goode received essentially identical shares of votes when listed first than when listed later (row 1 in Table S26).

***The 2012 gubernatorial election***. The vote shares for the Democratic (Maggie Hassan) and the Republican (Ovide Lamontagne) gubernatorial candidates in the 2012 election did not differ when the candidates were listed first and when they were listed later (row 1 and columns 1-2 in Table S29).

***The 2012 U.S. House of Representatives elections in Congressional District 1***. The vote shares that Democratic (Carol Shea-Porter), Republican (Frank Guinta), and third party (Brendan Kelly) congressional candidates in the Congressional District 1 received when listed first and when listed later were not statistically significantly different from one another (row 1 in Table S32).

***The 2012 U.S. House of Representatives elections in Congressional District 2***. The percentage of vote the Democratic candidate (Ann McLane Kuster) in the Congressional District 2 election received was statistically marginally significantly more when listed first than when listed in other positions on the ballot (b=.011, p<.10; row 1 and column 1 in Table S35). The Republican candidate (Charles Bass) and the other party candidate (Hardy Macia) received same votes when listed first as when listed in other positions on the ballot (row 1 and columns 2-3 in Tables S35).

**Name order effects in the 2016 general election.**

***The 2016 U.S. presidential election***. Both the Democratic and Republican presidential candidates received statistically significantly more votes when listed first than when listed later: 1.5 percentage points and 1.7 percentage points for Hillary Clinton and Donald Trump respectively (p=.071, p=.049; row 1 and columns 1-2 in Table S38). This first-on-the ballot effect was also present for two of the three non-major party presidential candidates, though the magnitude of the effect was much smaller: 0.12 percentage points for Jill Stein and 0.04 percentage points for Roque “Rocky” De La Fuente (p=.072, p=.082; row 1 and columns 4-5 in Table S38). There was no name order effect for candidate Gary Johnson (b=-.001, p=.629; row 1 and column 3 in Table S38).

***The 2016 U.S. Senate election***. Both the Democratic and Republican Senate candidates received more votes when listed first than when listed later: 1.5 percentage points and 1.2 percentage for Maggie Hassan and Kelly Ayotte respectively (p=.010, p=.026; row 1 and columns 1-2 in Table S41). Vote shares for the two other Senate candidates, Brian Chabot and Aaron Day did not differ depending on name order (row 1 and columns 3-4 in Table S41).

***The 2016 gubernatorial election***. Similar findings were obtained for the gubernatorial election. Both the Democratic and Republican gubernatorial candidates received more votes when listed first than when listed later: 1.4 percentage points and 1.6 percentage points for Colin Van Ostern and Chris Sununu, respectively (p=.071, p=.033; row 1 and columns 1-2 in Table S44). The vote share of the third-party gubernatorial candidate, Max Abramson, did not vary by name order (row 1 and column 3 in Table S44).

***The 2016 U.S. House of Representatives elections in Congressional District 1.*** The vote shares for the Democratic and Republican Congressional candidates (Carol Shea-Porter and Frank Guinta) as well as the three third=party candidates in the Congressional District 1 election did not differ by name order (columns 1-5 in Tables S47).

***The 2016 U.S. House of Representatives elections in Congressional District 2***. Vote shares for the Democratic Congressional candidate (Ann McLane Kuster) in the Congressional District 2 election did not differ depending on being listed first or later. In contrast, the Republican Congressional candidate (Jim Lawrence) received 2.1 percentage points more votes when listed first than when listed later (b=.015, p=.101; b=.021, p<.01; row 1 and columns 1-2 in Table S50). The third-party Congressional candidate (John J. Babiarz, Independent) received no additional votes when he was listed first compared to when he was listed later (row 1 and column 3 in Table S50).

1. **Robustness Checks of Name Order Effects**

**Robustness check 1: linear specification**.

***Linear specification in the three-candidate races in the 2000-2004 primaries*.** In the 2002 Republican primary for the U.S. Senate, one candidate, Smith, received one percentage point fewer votes for every position moving away from the first (p<.05; column 1 in Table S13). The other two candidates, Stremsky and Sununu, received the same percentage of votes regardless of the position, i.e., there was no linear name order effect for them (columns 3 and 5 in Table S13). These results were consistent with those reported in the manuscript.

In the 2004 Republican primary for the U.S. Senate, each of the three candidates received one percentage point fewer votes for every position moving away from the first (p<.05 and p<.001 for Aciere; p’s<.10 for Gregg; p’s<.001 for Tipa; row 1 in Table S15). These results were consistent with those reported in the manuscript.

***Linear specification in the greater than three-candidate races in the 2000-2004 primaries*.** In the five-candidate 2000 Republican primary for Governor, a primacy effect that was based on the specification reported in the manuscript was confirmed using the linear name order coding for Bramante (b=-.001, p<.10; row 1 and column 1 in Table S17), but not for Marron (row 1 and column 7 in Table S17). The absence of primacy effects for the other three candidates was also verified in the linear model (row 1 and columns 3, 5, and 9 in Table S17).

In the six-candidate 2002 Republican primary for Governor, a primacy effect that was based on the specification reported in the manuscript for one candidate, Haas, was not confirmed in the linear specification, whereas the absence of a primacy effect for other candidates were confirmed in the linear specification (row 1 in Table S19).

In the eight-candidate 2002 Republican primary for the 2nd Congressional District, whereas a primacy effect manifest for three candidates: Clark, Hoffman, and Mahoney, using the specification reported in the manuscript, a linear name order specification revealed a primacy effect for one of those three candidates (Hoffman; row 1 and column 5 in Table S22), but not for the other two (row 1 and columns 4 and 6 in Table S22). The absence of primacy effects for the other five candidates reported in the manuscript was also confirmed in the linear model (row 1 and columns 3, 5, and 9 in Table S22).

In the four-candidate 2004 Democratic primary for the 1st Congressional District, primacy effect occurred for all the candidates: Bruce, Duffey, Liles, and Nadeau, using the specification reported in the manuscript, and these primacy effects were confirmed with the linear name order specification for all but Bruce (row 1 and column 1 in Table S25): Duffy, Liles, and Nadeau received two, one, and three percentage points fewer votes respectively for every position they moved down the ballot from the first (p<.01,p<.001,p<.01; row 1 and columns 3, 5, and 7 in Table S25).

***Linear specification in the 2012 general election*.** In the 2012 presidential election, the linear specification confirmed the results reported in the manuscript of no primacy effect for presidential candidates Barack Obama, Mitt Romney, Gary Johnson, and Virgil Goode (row 1 in Table S27).

In the 2012 gubernatorial election, the absence of a primacy effect reported in the manuscript for the Democratic (Maggie Hassan) and the Republican (Ovide Lamontagne) gubernatorial candidates in the 2012 election was confirmed in the linear specification (row 1 and columns 1-2 in Table S30). However, there was a weak but negative effect of the linear specification on the vote share of the third party gubernatorial candidate (John J. Babiarz ) (b=-.002, p<.10; row 1 and column 3 in Table S30) though there was no primacy effect reported in the manuscript.

In the 2012 U.S. House of Representatives elections in Congressional District 1, the linear specification confirmed the absence of a primacy effect that was reported in the manuscript for Democratic (Carol Shea-Porter), Republican (Frank Guinta), and third party (Brendan Kelly) congressional candidates in the Congressional District 1 (row 1 in Table S33).

In the 2012 U.S. House of Representatives elections in Congressional District 2, the lack of a primacy effect documented in the manuscript for the Republican candidate (Charles Bass) was confirmed in the linear specification (row 1 and column 2 in Table S36). However, the primacy effect reported in the manuscript for the Democratic candidate (Ann McLane Kuster) was not confirmed in the linear specification (row 1 and column 1 in Table S36). Furthermore, there was a negative effect of name order on the third-party candidate’s vote share in the linear specification (b=-.005, p<.01; row 1 and column 3 in Table S36), though there was no primacy effect for this candidate in the analysis reported in the manuscript.

***Linear specification in the 2016 general election*.** In the 2016 U.S. presidential election, the primacy effects for both the Democratic and Republican presidential candidates reported in the manuscript were confirmed in the linear specification: there was a negative effect of name order on vote shares (b=-.010, p=.049; b=-.009, p=.057; for the Democratic and Republican presidential candidates, respectively; row 1 and columns 1-2 in Table S39). Primacy effects reported in the manuscript for two of the three non-major party presidential candidates, Jill Stein and Roque “Rocky” De La Fuente was also confirmed in the linear specification: there was a negative effect of name order (b=-.001; b=-.000, p’s<.10; for Jill Stein and Roque “Rocky” De La Fuente, respectively; row 1 and columns 4-5 in Table S39). Likewise, the lack of primacy effect reported in the manuscript for Gary Johnson was also confirmed in the linear specification (row 1 and column 3 in Table S39).

In the 2016 U.S. Senate election, the primacy effect reported in the manuscript for the Democratic candidate (Maggie Hassan) was confirmed in the linear specification ((b=-.010, p<.01; row 1 and column 1 in Table S42), however, the primacy effect reported in the manuscript for the Republican candidate (Kelly Ayotte) was not (row 1 and column 2 in Table S42). The absence of a primacy effect reported in the manuscript for the two other Senate candidates, Brian Chabot and Aaron Day, was confirmed in the linear specification (row 1 and columns 3-4 in Table S42).

In the 2016 gubernatorial election, a linear specification yielded a statistically significant negative effect of name order on the vote shares of the Democratic and Republican candidates (the Democratic candidate: b=-.099, p=.054; row 1 and column 1 in Table S45; the Republican candidate: b=-.010, p=.022), confirming the primacy effect reported in the manuscript for these candidates. However, whereas there was no primacy effect reported in the manuscript for the third-party candidate, the linear specification yielded a primacy effect (b=-.003, p=.015; row 1 and column 3 in Table S45).

In the 2016 U.S. House of Representatives elections in Congressional District 1, the absence of a primacy effect reported in the manuscript was confirmed for the Democratic (Carol Shea-Porter) and Republican (Frank Guinta) candidates as well as one third-party candidate (Brendan Kelly) in the linear specification (row 1 and columns 1-2 and 4 in Table S48). However, the linear specification yielded a primacy effect for the other two third-party candidates (Robert Lombardo and Shawn P. O’Connor) (b=-.001; b=-.004; respectively; p’s<.10, row 1 and columns 3 and 5 in Table S48) whereas the manuscript reported no primacy effect for them.

In the 2016 U.S. House of Representatives elections in Congressional District 2, the primacy effect reported in the manuscript for the Republican candidate (Jim Lawrence) was confirmed in the linear specification (b=.012, p<.01; row 1 and column 2 in Table S51), and so was the absence of a primacy effect reported in the manuscript for the third-party candidate (John J. Babiarz) (row 1 and column 3 in Table S51). However, the linear specification revealed a primacy effect for the Democratic candidate (Ann McLane Kuster) (b=-.011, p=.032; row 1 and column 1 in Table S51), whereas the manuscript reported no primacy effect for her.

**Robustness check 2: quadratic** **specification**.

***Quadratic specification in the three-candidate races in the 2000-2004 primaries*.** In the 2002 Republican primary for the U.S. Senate, there was no quadratic nonlinear effect of name order (row 2 in Table S13). Likewise, no quadratic nonlinear effect of name order was observed in the 2004 Republican primary for the U.S. Senate (row 2 in Table S15).

***Quadratic specification in the greater than three-candidate races in the 2000-2004 primaries*.** In the five-candidate 2000 Republican primary for Governor, there were no nonlinear effects of name order for any of the candidates in the race (row 2 in Table S17). In the six-candidate 2002 Republican primary for Governor, there were quadratic nonlinear name order effects for two of the six candidates (coefficient of name order squared term: .00, p<.05; .00, p<.001; for Haas and Kroepel, respectively; row 2 and columns 2 and 6 in Table S20) and not for others (row 2 and columns 1, 3-5 in Table S20). In the eight-candidate 2002 Republican primary for the 2nd Congressional District, there was a quadratic nonlinear name order effect for one out of the eight candidates (Mahoney; coefficient of name order squared term: .003, p<.05; row 2 and column 6 in Table S23), but not for any of the other candidates (row 2 and columns 1-5 and 7-8 in Table S23). In the four-candidate 2004 Democratic primary for the 1st Congressional District, there was a quadratic nonlinear name order effect for one out of the four candidates (Bruce; coefficient of name order squared term: .01, p<.05; row 2 and column 2 in Table S25), and not for any of the other three candidates (row 2 and columns 4, 6, and 8 in Table S25).

***Quadratic specification in the 2012 general election*.** In the 2012 U.S. presidential election, there was no quadratic nonlinear effect for any of the candidates (row 2 in Table S28), consistent with the no primacy effect finding reported in the manuscript. In the 2012 gubernatorial election, there was no quadratic nonlinear effect for the Democratic and Republican candidates (row 2 in Table S31), confirming there was no primacy effect for them as reported in the manuscript. However, the manuscript reported that the third party gubernatorial candidate (John J. Babiarz ) received same votes when listed first than when listed later but there was a quadratic nonlinear effect for him (coefficient of name order squared term: -.005, p<.01; row 2 and column 3 in Table S31). In the 2012 U.S. House of Representatives elections in Congressional District 1, there was a quadratic nonlinear effect of name order on the vote shares of the Republican and third-party Congressional candidates (coefficient of name order squared term: -.012, p<.10; -.011, p<.001 for Frank C. Guinta and Brendan Kelly, respectively; row 2 and columns 2-3 in Table S34), but not for the Democratic candidate (Carol Shea-Porter) (row 2 and column 1 in Table S34). In the 2012 U.S. House of Representatives elections in Congressional District 2, there was a quadratic nonlinear effect of name order on the vote shares of the third-party Congressional candidate (Hardy Macia) (coefficient of name order squared term: -.013, p<.001; row 1 and column 3 in Table S37), and not for the Democratic and Republican candidates (row 2 and columns 1-2 in Table S37).

***Quadratic specification in the 2016 general election*.** In the 2016 U.S. presidential election, there was no quadratic nonlinear effect for any of the five candidates (row 2 in Table S40). In the 2016 U.S. Senate election, name order influenced the vote shares for the Republican and one third-party candidates nonlinearly (coefficient of name order squared term: .009, p<.10; -.002, p<.10; for Kelly Ayotte and Aaron Day, respectively; row 2 and columns 2 and 4 in Table S43) and did not for the Democratic and the other third-party candidates (row 2 and columns 1 and 3 in Table S43). In the 2016 gubernatorial election, there was no nonlinear effect of name order on the vote shares of the Democratic (Colin Van Ostern) or the Republican (Chris Sununu) gubernatorial candidates (row 2 and columns 1-2 in Table S46), whereas the effect was nonlinear for the third party candidate (Max Abramson) (coefficient of name order squared term: -.009, p<.001; row 2 and column 3 in Table S46). In the 2016 U.S. House of Representatives elections in Congressional District 1**,** there was a quadratic nonlinear effect for two of the three third-party candidates (coefficient of name order squared term: -.003, p<.05; -.014, p<.001; for Brendan Kelly Shawn P. O’Connor, respectively; row 2 and columns 4-5 in Table S49), and not for the Democratic (Carol Shea-Porter), the Republican (Frank Guinta), or the other third-party candidate (Robert Lombardo) (row 2 and columns 1-3 in Table S49). In the 2016 U.S. House of Representatives elections in Congressional District 2, there was a quadratic nonlinear effect for the third-party candidate (John J. Babiarz) (coefficient of name order squared term: -.010, p<.01; row 2 and column 3 in Table S52), but for the Democratic (Ann McLane Kuster) or the Republican (Jim Lawrence) candidates (row 2 and columns 1-2 in Table S52).

**Robustness check 3: dummy variables**. In addition to the quadratic specification to check for nonlinear name order effects, regressions with an alternative dummy variables specification in which two dummy variables were created (*second versus first* and *third versus first*) were coded 1 for being listed second and third, respectively, and 0 otherwise, were also conducted. This specification is less restrictive than the quadratic specification.

Results with this dummy variables specification (4) are consistent with the results obtained using the quadratic (nonlinear) specification (Tables SC1-SC6 in Appendix C for the 2000, 2002, and 2004 primaries; Tables SC7-SC10 in Appendix C for the 2012 general election; Tables SC11-SC15 in in Appendix C for the 2016 general election).

**Robustness check 4: weights applied in the general election analyses**. Past research has argued that small towns introduce greater variance in candidates’ vote shares, and that such sources of heteroscedasticity should be accounted for when a considerable portion of towns are small (defined as having no more than 100 votes;e.g. Grant 2017). To account for the heteroscedasticity, a weight based on town size may be employed. In the 2016 election, only four out of 300 towns had fewer than 100 votes, so heteroscedasticity is not of concern; nevertheless, as a robustness check, we constructed weights to be one-half of the base-10 logarithm of the number of voters cast and reran the analysis. Results with weights applied were nearly identical to results without weights (Tables SC16-SC31 in Appendix C for the 2012 general election; Tables SC32-SC51 in Appendix C for the 2016 general election).

**Robustness check 5: alternative coding for party column rotation in the general election analyses**. Rather than coding the multiple (when applicable) third party candidates in the general election analyses to have the identical name order, an alternative was to code these candidates as having sequential name order, in ascending order of their vertical position (as described previously). This approach was applied to the following races: the 2016 presidential race (three non-major party candidates), the 2016 U.S. Senate race (two non-major party candidates), the 2016 Congressional District 1 race (three non-major party candidates), and the 2012 presidential race (two non-major party candidates). The name order results were robust to this alternative name order coding for the 2016 presidential race (Table SC54 with the alternative approach in the linear specification; Table SC55 with the alternative approach in the quadratic specification); the 2016 U.S. Senate race (Table SC56 with the alternative approach in the linear specification; Table SC57 with the alternative approach in the quadratic specification); the 2016 Congressional District 1 race (Table SC58 with the alternative approach in the linear specification; Table SC59 with the alternative approach in the quadratic specification), and the 2012 presidential race (Table SC52 with the alternative approach in the linear specification; Table SC53 with the alternative approach in the quadratic specification).

**4. Discussion on the 2016 Presidential Election**

President Trump won the electoral college vote by a margin of 77 votes, with very slim margins of popular vote victories in four states: Michigan (margin of 0.22%, 16 electoral votes), Wisconsin (margin of 0.76%, 10 electoral votes), Florida (margin of 1.20%, 29 electoral votes), and Pennsylvania (margin of .72%, 20 electoral votes). In this light, it is interesting to consider the procedure for ordering of names on ballots in those states and what might have happened if name order had been rotated.

President Trump was listed first on all ballots in three of the four states that he won by very small margins (Michigan, Wisconsin, and Florida), and in Pennsylvania, Secretary Clinton was always listed first. In Minnesota, where Secretary Clinton won 14 electoral votes by a very tight margin (margin of 1.52%, 10 electoral votes). President Trump was listed first on all ballots. If Michigan, Wisconsin, and Florida had rotated candidate name order across precincts, and if name order effects in those states were at least as large as those observed in the New Hampshire presidential race, the election outcome would have flipped.

Such a flip requires only 39 electoral votes to shift from President Trump to Secretary Clinton, and 55 of President Trump’s electoral votes were from Michigan (16), Wisconsin (10), and Florida (29). had name order been rotated in Michigan, President Trump would not have been first in five-sixths of the precincts (because 6 candidates competed there), so he would have lost 1.4 percentage points as a result (assuming name order effects similar in size to that of New Hampshire). Secretary Clinton would have been listed first in one-sixth of the precincts, yielding a net gain of 0.3% for her. The net loss of 1.4% for President Trump and the net gain of 0.3% for Secretary Clinton would have changed the margin of victory by 1.7%, much more than President Trump’s 0.22% margin of victory, thereby flipping 16 electoral votes to Secretary Clinton.

Likewise, had name order been rotated in Wisconsin, President Trump would not have been first in six-sevenths of the precincts (because 7 candidates competed there), so he would have lost 1.3 percentage points as a result. Secretary Clinton would have been listed first in one-seventh of the precincts, yielding a net gain of 0.4% for her. The net loss of 1.5% for President Trump and the net gain of 0.2% for Secretary Clinton would have changed the margin of victory by 1.7%, much more than Trump’s 0.76% margin of victory, thereby flipping ten electoral votes to Secretary Clinton.

And had name order been rotated in Florida, President Trump would not have been first in three-fourths of the precincts (because 4 candidates competed there), so he would have lost 1.3 percentage points as a result. Secretary Clinton would have been listed first in one-fourth of the precincts, yielding a net gain of 0.4% for her. The net loss of 1.3% for President Trump and the net gain of 0.4% for Secretary Clinton would have changed the margin of victory by 1.7%, more than President Trump’s 1.20% margin of victory, thereby flipping 29 electoral votes to Clinton.

In sum, had name order been rotated in Michigan, Wisconsin, and Florida, and had the name order effect been as large or nearly so in those states as in New Hampshire, President Trump would have lost in these three states, and 55 of his electoral votes would have shifted from him to Secretary Clinton, thereby flipping the nationwide election outcome.

**5. Figures**

Figure S1. Reproduced Table 1 in Koppell and Steen (2004)

Figure S2. Reproduced Table 2 in Koppell and Steen (2004)

Figure S3. Reproduced Table 4 in Grant (2017)


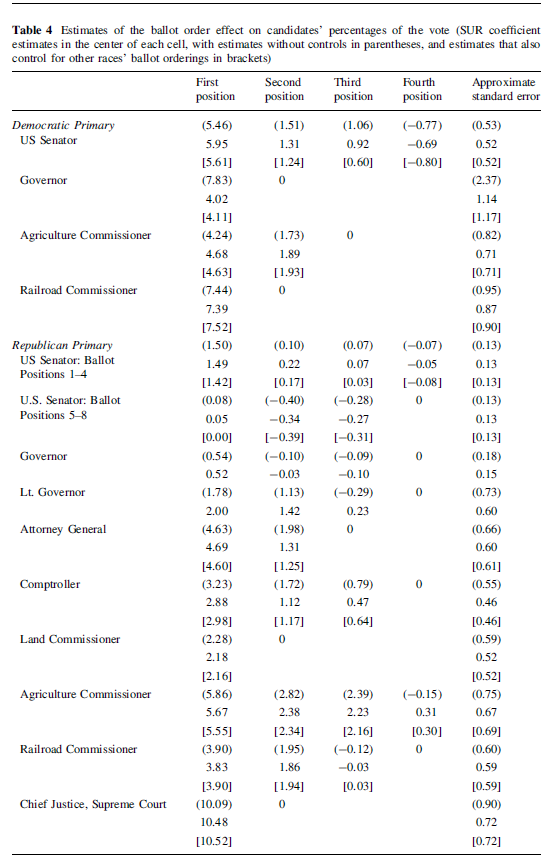

Figure S4. 2012 General Election Ballot of Alstead, New Hampshire

Figure S5. 2016 General Election Ballot of Acworth, New Hampshire

**6. Tables**

Table A1

Name Order Effects of 2000-2004 Primary Candidates and 2012-2016 General Election Candidates in New Hampshire

| Race | Candidate | Direction of Effect (1) | Effect (2) | p-value  (3) | p-value from Randomization Inference (4) |
| --- | --- | --- | --- | --- | --- |
| 2000 Primary, Governor, Democrat | Fernald | Recency | -.003 | .884 | .887 |
| 2000 Primary, Governor, Democrat | Shaheen | Recency | -.002 | .909 | .908 |
|  | |  |  |  |  |
| 2000 Primary, Governor, Republican | Bramante | Primacy | .005* | .024 | .026 |
| 2000 Primary, Governor, Republican | Howard | Primacy | .010 | .417 | .427 |
| 2000 Primary, Governor, Republican | Humphrey | Primacy | .019 | .248 | .256 |
| 2000 Primary, Governor, Republican | Marron | Primacy | .003* | .047 | .047 |
| 2000 Primary. Governor, Republican | Squires | Primacy | .001 | .936 | .933 |
|  |  |  |  |  |  |
| 2000 Primary, CD 2, Democrat | Brannen | Primacy | .011 | .558 | .566 |
| 2000 Primary, CD 2, Democrat | Jackman | Primacy | .014 | .447 | .451 |
|  | |  |  |  |  |
| 2002 Primary, Governor, Democrat | Fernald | Primacy | .023 | .162 | .157 |
| 2002 Primary, Governor, Democrat | Hollingworth | Primacy | .014 | .380 | .381 |
|  | |  |  |  |  |
| 2002 Primary, Governor, Republican | Benson | Recency | -.003 | .812 | .822 |
| 2002 Primary, Governor, Republican | Hass | Primacy | .003* | .024 | .043 |
| 2002, Primary Governor, Republican | Humphrey | Primacy | .013 | .329 | .329 |
| 2002 Primary, Governor, Republican | Keough | Primacy | .012 | .430 | .442 |
| 2002 Primary, Governor, Republican | Kingsbury | Primacy | .000 | .659 | .657 |
| 2002 Primary, Governor, Republican | Kroepel | Primacy | .001 | .292 | .277 |
|  |  |  |  |  |  |
| 2002 Primary, U.S. Senate, Republican | Smith | Primacy | .017+ | .076 | .076 |
| 2002 Primary, U.S. Senate, Republican | Stremsky | Recency | -.000 | .895 | .891 |
| 2002 Primary, U.S. Senate, Republican | Sununu | Recency | -.000 | .967 | .976 |
|  |  |  |  |  |  |
| 2002 Primary, CD 1, Democrat | Clark | Primacy | .016 | .245 | .257 |
| 2002 Primary, CD 1, Democrat | Dean | Primacy | .015 | .260 | .271 |
|  | |  |  |  |  |
| 2002 Primary, CD 1, Republican | Barrows | Primacy | .007 | .686 | .689 |
| 2002 Primary, CD 1, Republican | Bevill | Primacy | .000 | .984 | .987 |
| 2002 Primary, CD 1, Republican | Bradley | Primacy | .028 | .423 | .423 |
| 2002 Primary, CD 1, Republican | Clark | Primacy | .036* | .041 | .048 |
| 2002 Primary, CD 1, Republican | Hoffman | Primacy | .009** | .002 | .002 |
| 2002 Primary, CD 1, Republican | Mahoney | Primacy | .045+ | .057 | .057 |
| 2002 Primary, CD 1, Republican | Stephen | Primacy | .041 | .228 | .232 |
| 2002 Primary, CD 1, Republican | Wendelboe | Primacy | .009 | .555 | .478 |
|  |  |  |  |  |  |
| 2002 Primary, CD 2, Democrat | Jackman | Primacy | .023* | .047 | .044 |
| 2002 Primary, CD 2, Democrat | Swett | Primacy | .026* | .033 | .033 |
|  | |  |  |  |  |
| 2002 Primary, CD 2, Republican | Bass | Primacy | .006 | .386 | .405 |
| 2002 Primary, CD 2, Republican | Douglass | Primacy | .006 | .360 | .359 |
|  | |  |  |  |  |
| 2004 Primary, Governor, Democrat | Lynch | Primacy | .022 | .176 | .178 |
| 2004 Primary, Governor, Democrat | McEachern | Primacy | .021 | .207 | .212 |
|  | |  |  |  |  |
| 2004 Primary, Governor, Republican | Benson | Primacy | .030* | .035 | .036 |
| 2004 Primary, Governor, Republican | Tarbell | Primacy | .029** | .009 | .010 |
|  | |  |  |  |  |
| 2004 Primary, U.S. Senate, Republican | Alciere | Primacy | .013*** | .000 | .000 |
| 2004, Primary U.S. Senate, Republican | Gregg | Primacy | .017* | .044 | .034 |
| 2004, Primary U.S. Senate, Republican | Tipa | Primacy | .010* | .012 | .010 |
|  |  |  |  |  |  |
| 2004 Primary, CD 1, Democrat | Bruce | Primacy | .046* | .041 | .042 |
| 2004 Primary, CD 1, Democrat | Duffy | Primacy | .063* | .011 | .010 |
| 2004 Primary, CD 1, Democrat | Liles | Primacy | .018** | .002 | .002 |
| 2004 Primary, CD 1, Democrat | Nadeau | Primacy | .071+ | .056 | .056 |
|  |  |  |  |  |  |
| 2004 Primary, CD 1, Republican | Bevill | Primacy | .007 | .338 | .333 |
| 2004 Primary, CD 1, Republican | Bradley | Primacy | .005 | .504 | .507 |
|  | |  |  |  |  |
| 2004 Primary, CD 2, Democrat | Hodes | Primacy | .071** | .001 | .002 |
| 2004 Primary, CD 2, Democrat | Owen | Primacy | .066** | .002 | .001 |
|  | |  |  |  |  |
| 2004 Primary, CD 2, Republican | Bass | Primacy | .034* | .041 | .043 |
| 2004 Primary, CD 2, Republican | Brady | Primacy | .033* | .043 | .045 |
|  |  |  |  |  |  |
| 2012 General, President | Obama (Democrat) | Primacy | .000 | .955 | .958 |
| 2012 General, President | Romney (Republican) | Recency | -.002 | .688 | .723 |
| 2012 General, President | Johnson (Other) | Primacy | .000 | .945 | .981 |
| 2012 General, President | Goode (Other) | Primacy | .000 | .917 | .921 |
|  |  |  |  |  |  |
| 2012 General, Governor | Hassan (Democrat) | Primacy | .002 | .720 | .769 |
| 2012 General, Governor | Lamontagne (Republican) | Primacy | .006 | .323 | .394 |
| 2012 General, Governor | Babiarz (Other) | Recency | -.000 | .924 | .931 |
|  |  |  |  |  |  |
| 2012 General, CD1 | Shea-Porter (Democrat) | Recency | -.003 | .759 | .760 |
| 2012 General, CD1 | Guinta (Republican) | Recency | -.009 | .258 | .268 |
| 2012 General, CD1 | Kelly (Other) | Recency | -.001 | .624 | .640 |
|  |  |  |  |  |  |
| 2012 General, CD2 | Kuster (Democrat) | Primacy | .011^+^ | .072 | .186 |
| 2012 General, CD2 | Bass (Republican) | Primacy | .008 | .177 | .320 |
| 2012 General, CD2 | Macia (Other) | Primacy | .000 | .860 | .915 |
|  |  |  |  |  |  |
| 2016 General, President | Clinton (Democrat) | Primacy | .015^+^ | .071 | .075 |
| 2016 General, President | Trump (Republican) | Primacy | .017* | .049 | .043 |
| 2016 General, President | Johnson (Other) | Recency | -.001 | .629 | .629 |
| 2016 General, President | Stein (Other) | Primacy | .001^+^ | .072 | .072 |
| 2016 General, President | De La Fuente (Other) | Primacy | .000^+^ | .082 | .079 |
|  |  |  |  |  |  |
| 2016 General, Governor | Ostern (Democrat) | Primacy | .014^+^ | .071 | .070 |
| 2016 General, Governor | Sununu (Republican) | Primacy | .016* | .033 | .027 |
| 2016 General, Governor | Abramson (Other) | Recency | .000 | 1.000 | 1.000 |
|  |  |  |  |  |  |
| 2016 General, U.S. Senate | Hassan (Democrat) | Primacy | .015* | .010 | .010 |
| 2016 General, U.S. Senate | Ayotte (Republican) | Primacy | .012* | .026 | .025 |
| 2016 General, U.S. Senate | Chabot (Other) | Recency | -.000 | .830 | .832 |
| 2016 General, U.S. Senate | Day (Other) | Primacy | .000 | .815 | .813 |
|  |  |  |  |  |  |
| 2016 General, CD1 | Shea-Porter (Democrat) | Primacy | .007 | .320 | .319 |
| 2016 General, CD1 | Guinta (Republican) | Primacy | .005 | .499 | .504 |
| 2016 General, CD1 | Lombardo (Other) | Primacy | .001 | .259 | .253 |
| 2016 General, CD1 | Kelly (Other) | Recency | -.000 | .775 | .772 |
| 2016 General, CD1 | O’Connor (Other) | Recency | -.001 | .878 | .880 |
|  |  |  |  |  |  |
| 2016 General, CD2 | Kuster (Democrat) | Primacy | .015 | .101 | .103 |
| 2016 General, CD2 | Lawrence (Republican) | Primacy | .021** | .008 | .008 |
| 2016 General, CD2 | Babiarz (Other) | Recency | -.000 | .894 | .893 |

Note: Cell entries in column (2) are OLS regression coefficients (p-values in column (3)) predicting each candidate’s vote share using whether the candidate was listed first and other predictors, including total votes cast (in all elections) and the percent of registered voters who were registered as Democrats and the percent of registered voters who were registered as Republicans in the general elections. Coefficients for other predictors are not shown.

***p < .001 **p < .01 *p < .05 +p < .10

Table S1

*Name Order Effects in the Two-Candidate 2000 Democratic Primary for Governor in New Hampshire: First Versus Last*

|  | Dependent Variable | |
| --- | --- | --- |
| Predictor | Vote share of Fernald | Vote share of Shaheen |
| Listed first (vs. later) | -.003 | -.002 |
|  | (.02) | (.02) |
| Total number of votes (in 000s) | -.13** | .14*** |
|  | (.04) | (.04) |
| Constant | .44*** | .52*** |
|  | (.02) | (.02) |
|  |  |  |
| R^2^ | .03 | .04 |
| N | 301 | 301 |

Notes: Cell entries are regression coefficients predicting each candidate’s vote share using the predictors listed in the first column with standard errors in parentheses.

***p<.001 **p<.01 *p<.05 +p<.10

Table S2

*Name Order Effects in the Two-Candidate 2000 Democratic Primary for 2nd Congressional District in New Hampshire: First Versus Last*

|  | Dependent Variable | |
| --- | --- | --- |
| Predictor | Vote share of Brannen | Vote share of Jackman |
| Listed first (vs. later) | .01 | .01 |
|  | (.02) | (.02) |
| Total number of votes (in 000s) | .26*** | -.21 |
|  | (.06) | (.06) |
| Constant | .60*** | .37*** |
|  | (.02) | (.02) |
|  |  |  |
| R^2^ | .09 | .07 |
| N | 186 | 186 |

Notes: Cell entries are regression coefficients predicting each candidate’s vote share using the predictors listed in the first column with standard errors in parentheses.

***p<.001 **p<.01 *p<.05 +p<.10

Table S3

*Name Order Effects in the Two-Candidate 2002 Democratic Primary for Governor in New Hampshire: First Versus Last*

|  | Dependent Variable | |
| --- | --- | --- |
| Predictor | Vote share of Fernald | Vote share of Hollingsworth |
| Listed first (vs. later) | .02 | .01 |
|  | (.02) | (.02) |
| Total number of votes (in 000s) | -.12** | .12** |
|  | (.04) | (.04) |
| Constant | .57*** | .37*** |
|  | (.02) | (.01) |
|  |  |  |
| R^2^ | .03 | .03 |
| N | 302 | 302 |

Notes: Cell entries are regression coefficients predicting each candidate’s vote share using the predictors listed in the first column with standard errors in parentheses.

***p<.001 **p<.01 *p<.05 +p<.10

Table S4

*Name Order Effects in the Two-Candidate 2002 Democratic Primary for 1st Congressional District in New Hampshire: First Versus Last*

|  | Dependent Variable | |
| --- | --- | --- |
| Predictor | Vote share of Clark | Vote share of Dean |
| Listed first (vs. later) | .02 | .02 |
|  | (.01) | (.01) |
| Total number of votes (in 000s) | -.05+ | .05 |
|  | (.03) | (.03) |
| Constant | .86*** | .12*** |
|  | (.01) | (.01) |
|  |  |  |
| R^2^ | .04 | .03 |
| N | 114 | 114 |

Notes: Cell entries are regression coefficients predicting each candidate’s vote share using the predictors listed in the first column with standard errors in parentheses.

***p<.001 **p<.01 *p<.05 +p<.10

Table S5

*Name Order Effects in the Two-Candidate 2002 Democratic Primary for 2nd Congressional District in New Hampshire: First Versus Last*

|  | Dependent Variable | |
| --- | --- | --- |
| Predictor | Vote share of Jackman | Vote share of Swett |
| Listed first (vs. later) | .02* | .03* |
|  | (.01) | (.01) |
| Total number of votes (in 000s) | -.01 | .004 |
|  | (.04) | (.04) |
| Constant | .20*** | .77*** |
|  | (.01) | (.01) |
|  |  |  |
| R^2^ | .02 | .03 |
| N | 186 | 186 |

Notes: Cell entries are regression coefficients predicting each candidate’s vote share using the predictors listed in the first column with standard errors in parentheses.

***p<.001 **p<.01 *p<.05 +p<.10

Table S6

*Name Order Effects in the Two-Candidate 2002 Republican Primary for 2nd Congressional District in New Hampshire: First Versus Last*

|  | Dependent Variable | |
| --- | --- | --- |
| Predictor | Vote share of Bass | Vote share of Douglass |
| Listed first (vs. later) | .01 | .01 |
|  | (.01) | (.01) |
| Total number of votes (in 000s) | .01 | -.01 |
|  | (.01) | (.01) |
| Constant | .86*** | .13*** |
|  | (.01) | (.01) |
|  |  |  |
| R^2^ | .01 | .01 |
| N | 188 | 188 |

Notes: Cell entries are regression coefficients predicting each candidate’s vote share using the predictors listed in the first column with standard errors in parentheses.

***p<.001 **p<.01 *p<.05 +p<.10

Table S7

*Name Order Effects in the Two-Candidate 2004 Democratic Primary for Governor in New Hampshire: First Versus Last*

|  | Dependent Variable | |
| --- | --- | --- |
| Predictor | Vote share of Lynch | Vote share of McEachern |
| Listed first (vs. later) | .02 | .02 |
|  | (.02) | (.02) |
| Total number of votes (in 000s) | .11* | -.10* |
|  | (.04) | (.04) |
| Constant | .69*** | .27*** |
|  | (.01) | (.02) |
|  |  |  |
| R^2^ | .03 | .02 |
| N | 300 | 300 |

Notes: Cell entries are regression coefficients predicting each candidate’s vote share using the predictors listed in the first column with standard errors in parentheses.

***p<.001 **p<.01 *p<.05 +p<.10

Table S8

*Name Order Effects in the Two-Candidate 2004 Republican Primary for Governor in New Hampshire: First Versus Last*

|  | Dependent Variable | |
| --- | --- | --- |
| Predictor | Vote share of Benson | Vote share of Tarbell |
| Listed first (vs. later) | .03* | .03** |
|  | (.01) | (.01) |
| Total number of votes (in 000s) | .13*** | -.07*** |
|  | (.03) | (.02) |
| Constant | .66*** | .23*** |
|  | (.01) | (.01) |
|  |  |  |
| R^2^ | .08 | .06 |
| N | 302 | 302 |

Notes: Cell entries are regression coefficients predicting each candidate’s vote share using the predictors listed in the first column with standard errors in parentheses.

***p<.001 **p<.01 *p<.05 +p<.10

Table S9

*Name Order Effects in the Two-Candidate 2004 Republican Primary for 1st Congressional District in New Hampshire: First Versus Last*

|  | Dependent Variable | |
| --- | --- | --- |
| Predictor | Vote share of Bevill | Vote share of Bradley |
| Listed first (vs. later) | .01 | .01 |
|  | (.01) | (.01) |
| Total number of votes (in 000s) | .02 | -.02 |
|  | (.02) | (.01) |
| Constant | .09*** | .90*** |
|  | (.01) | (.01) |
|  |  |  |
| R^2^ | .02 | .02 |
| N | 114 | 114 |

Notes: Cell entries are regression coefficients predicting each candidate’s vote share using the predictors listed in the first column with standard errors in parentheses.

***p<.001 **p<.01 *p<.05 +p<.10

Table S10

*Name Order Effects in the Two-Candidate 2004 Democratic Primary for 2nd Congressional District in New Hampshire: First Versus Last*

|  | Dependent Variable | |
| --- | --- | --- |
| Predictor | Vote share of Hodes | Vote share of Owen |
| Listed first (vs. later) | .07*** | .07** |
|  | (.02) | (.02) |
| Total number of votes (in 000s) | .36*** | -.31*** |
|  | (.08) | (.07) |
| Constant | .46*** | .44*** |
|  | (.02) | (.02) |
|  |  |  |
| R^2^ | .16 | .14 |
| N | 186 | 186 |

Notes: Cell entries are regression coefficients predicting each candidate’s vote share using the predictors listed in the first column with standard errors in parentheses.

***p<.001 **p<.01 *p<.05 +p<.10

Table S11

*Name Order Effects in the Two-Candidate 2004 Republican Primary for 2nd Congressional District in New Hampshire: First Versus Last*

|  | Dependent Variable | |
| --- | --- | --- |
| Predictor | Vote share of Bass | Vote share of Brady |
| Listed first (vs. later) | .03* | .03* |
|  | (.02) | (.02) |
| Total number of votes (in 000s) | .01 | -.01 |
|  | (.04) | (.04) |
| Constant | .69*** | .27*** |
|  | (.01) | (.01) |
|  |  |  |
| R^2^ | .02 | .02 |
| N | 188 | 188 |

Notes: Cell entries are regression coefficients predicting each candidate’s vote share using the predictors listed in the first column with standard errors in parentheses.

***p<.001 **p<.01 *p<.05 +p<.10

Table S12

*Name Order Effects in the Three-Candidate 2002 Republican Primary for US Senate in New Hampshire: First Versus Second/Third*

|  | Dependent Variable | | |
| --- | --- | --- | --- |
| Predictor | Vote share of Smith | Vote share of Stremsky | Vote share of Sununu |
| Listed first (vs. later) | .02+ | .00 | .00 |
|  | (.01) | (.002) | (.01) |
| Total number of votes (in 000s) | -.04*** | -.002 | .04*** |
|  | (.01) | (.001) | (.01) |
| Constant | .49*** | .02*** | .49*** |
|  | (.01) | (.001) | (.01) |
|  |  |  |  |
| R^2^ | .08 | .01 | .08 |
| N | 302 | 302 | 302 |

Notes: Cell entries are regression coefficients predicting each candidate’s vote share using the predictors listed in the first column with standard errors in parentheses.

***p<.001 **p<.01 *p<.05 +p<.10

Table S13

*Name Order Effects in the Three-Candidate 2002 Republican Primary for US Senate in New Hampshire: Name Order and Name Order Squared*

|  | Dependent Variable | | | | | |
| --- | --- | --- | --- | --- | --- | --- |
| Predictor | Model 1 Vote share of Smith | Model 2 Vote share of Smith | Model 1 Vote share of Stremsky | Model 2 Vote share of Stremsky | Model 1 Vote share of Sununu | Model 2 Vote share of Sununu |
| Name order | -.01* | -.01* | -.001 | -.001 | -.01 | -.01 |
|  | (.01) | (.01) | (.001) | (.001) | (.01) | (.01) |
| Name order squared |  | -.002 |  | -.003 |  | -.02+ |
|  |  | (.01) |  | (.002) |  | (.01) |
| Total number of votes (in 000s) | -.04*** | -.04*** | -.002 | -.002 | .04*** | .04*** |
|  | (.01) | (.01) | (.001) | (.001) | (.01) | (.01) |
| Constant | .49*** | .49*** | .02*** | .02*** | .49*** | .50*** |
|  | (.01) | (.01) | (.001) | (.002) | (.01) | (.01) |
|  |  |  |  |  |  |  |
| R^2^ | .09 | .09 | .01 | .02 | .08 | .09 |
| N | 302 | 302 | 302 | 302 | 302 | 302 |

Notes: Cell entries are regression coefficients predicting each candidate’s vote share using the predictors listed in the first column with standard errors in parentheses.

***p<.001 **p<.01 *p<.05 +p<.10

Table S14

*Name Order Effects in the Three-Candidate* *2004 Republican Primary for US Senate in New Hampshire: First Versus Second/Third*

|  | Dependent Variable | | |
| --- | --- | --- | --- |
| Predictor | Vote share of Aciere | Vote share of Gregg | Vote share of Tipa |
| Listed first (vs. later) | .01*** | .02* | .01* |
|  | (.001) | (.01) | (.004) |
| Total number of votes (in 000s) | .002 | .02 | -.003 |
|  | (.01) | (.02) | (.01) |
| Constant | .04*** | .90*** | .04*** |
|  | (.002) | (.01) | (.003) |
|  |  |  |  |
| R^2^ | .05 | .02 | .02 |
| N | 302 | 302 | 302 |

Notes: Cell entries are regression coefficients predicting each candidate’s vote share using the predictors listed in the first column with standard errors in parentheses.

***p<.001 **p<.01 *p<.05 +p<.10

Table S15

*Name Order Effects in the Three-Candidate 2004 Republican Primary for US Senate in New Hampshire: Name Order and Name Order Squared*

|  | Dependent Variable | | | | | |
| --- | --- | --- | --- | --- | --- | --- |
| Predictor | Model 1 Vote share of Aciere | Model 2 Vote share of Aciere | Model 1 Vote share of Gregg | Model 2 Vote share of Gregg | Model 1 Vote share of Tipa | Model 2 Vote share of Tipa |
| Name order | -.01* | -.01*** | -.01+ | -.01+ | -.01*** | -.01*** |
|  | (.002) | (.002) | (.01) | (.01) | (.002) | (.002) |
| Name order squared |  | -.001 |  | .01 |  | -.003 |
|  |  | (.003) |  | (.01) |  | (.004) |
| Total number of votes (in 000s) | .002 | .002 | .02 | .02 | -.002 | -.002 |
|  | (.01) | (.01) | (.02) | (.02) | (.01) | (.01) |
| Constant | .04*** | .04*** | .91*** | .90*** | .04*** | .04*** |
|  | (.002) | (.003) | (.01) | (.01) | (.002) | (.003) |
|  |  |  |  |  |  |  |
| R^2^ | .08 | .08 | .01 | .02 | .04 | .04 |
| N | 302 | 302 | 302 | 302 | 302 | 302 |

Notes: Cell entries are regression coefficients predicting each candidate’s vote share using the predictors listed in the first column with standard errors in parentheses.

***p<.001 **p<.01 *p<.05 +p<.10

Table S16

*Name Order Effects in* *the Five-Candidate 2000 Republican Primary for Governor in New Hampshire: First Versus Later*

|  | Dependent Variable | | | | |
| --- | --- | --- | --- | --- | --- |
| Predictor | Vote share of Bramante | Vote share of Howard | Vote share of Humphrey | Vote share of Marron | Vote share of Squires |
| Listed first (vs. later) | .01* | .01 | .02 | .003* | .001 |
|  | (.002) | (.01) | (.02) | (.001) | (.02) |
| Total number of votes (in 000s) | .01* | -.01 | -.05* | -.003+ | .06*** |
|  | (.003) | (.02) | (.02) | (.002) | (.02) |
| Constant | .02*** | .21*** | .55*** | .01*** | .19*** |
|  | (.001) | (.01) | (.01) | (.001) | (.01) |
|  |  |  |  |  |  |
| R^2^ | .03 | .003 | .02 | .02 | .04 |
| N | 300 | 300 | 300 | 300 | 300 |

Notes: Cell entries are regression coefficients predicting each candidate’s vote share using the predictors listed in the first column with standard errors in parentheses.

***p<.001 **p<.01 *p<.05 +p<.10

Table S17

*Name Order Effects in the Five-Candidate 2000 Republican Primary for Governor in New Hampshire: Name Order and Name Order Squared*

|  | Dependent Variable | | | | | | | | | |
| --- | --- | --- | --- | --- | --- | --- | --- | --- | --- | --- |
| Predictor | Model 1 Vote share of Bramante | Model 2 Vote share of Bramante | Model 1 Vote share of Howard | Model 2 Vote share of Howard | Model 1 Vote share of Humphrey | Model 2 Vote share of Humphrey | Model 1 Vote share of Marron | Model 2 Vote share of Marron | Model 1 Vote share of Squires | Model 2 Vote share of Squires |
| Name order | -.001+ | -.001+ | -.003 | -.003 | -.004 | -.004 | .00 | .00 | -.001 | -.001 |
|  | (.001) | (.001) | (.004) | (.004) | (.01) | (.01) | (.00) | (.00) | (.004) | (.004) |
| Name order squared |  | .001 |  | -.002 |  | .003 |  | .001* |  | .001 |
|  |  | (.001) |  | (.003) |  | (.004) |  | (.00) |  | (.004) |
| Total number of votes (in 000s) | .01* | .01* | -.01 | -.01 | -.05* | -.05* | -.003 | -.003+ | .06*** | .06*** |
|  | (.003) | (.003) | (.02) | (.02) | (.02) | (.02) | (.002) | (.002) | (.02) | (.02) |
| Constant | .02*** | .02*** | .21*** | .22*** | .55*** | .54*** | .01*** | .01*** | .19*** | .19*** |
|  | (.001) | (.002) | (.01) | (.01) | (.01) | (.01) | (.001) | (.001) | (.01) | (.01) |
|  |  |  |  |  |  |  |  |  |  |  |
| R^2^ | .03 | .03 | .003 | .01 | .02 | .02 | .01 | .03 | .04 | .04 |
| N | 300 | 300 | 300 | 300 | 300 | 300 | 300 | 300 | 300 | 300 |

Notes: Cell entries are regression coefficients predicting each candidate’s vote share using the predictors listed in the first column with standard errors in parentheses.

***p<.001 **p<.01 *p<.05 +p<.10

Table S18

*Name Order Effects in* *the Six-Candidate 2002 Republican Primary for Governor in New Hampshire: First Versus Later*

|  | Dependent Variable | | | | | |
| --- | --- | --- | --- | --- | --- | --- |
| Predictor | Vote share of Benson | Vote share of Haas | Vote share of Humphrey | Vote share of Keough | Vote share of Kingsbury | Vote share of Kroepel |
| Listed first (vs. later) | .003 | .003* | .01 | .01 | .00 | .001 |
|  | (.01) | (.001) | (.01) | (.02) | (.001) | (.001) |
| Total number of votes (in 000s) | .01 | .00 | .00 | -.01 | -.001* | -.001 |
|  | (.01) | (.001) | (.01) | (.01) | (.001) | (.001) |
| Constant | .36*** | .01*** | .28*** | .34*** | .01*** | .01*** |
|  | (.01) | (.01) | (.01) | (.01) | (.001) | (.001) |
|  |  |  |  |  |  |  |
| R^2^ | .01 | .02 | .003 | .003 | .01 | .01 |
| N | 302 | 302 | 302 | 302 | 302 | 302 |

Notes: Cell entries are regression coefficients predicting each candidate’s vote share using the predictors listed in the first column with standard errors in parentheses.

***p<.001 **p<.01 *p<.05 +p<.10

Table S19

*Name Order Effects in the Six-Candidate 2002 Republican Primary for Governor in New Hampshire: Name Order*

|  | Dependent Variable | | | | | |
| --- | --- | --- | --- | --- | --- | --- |
| Predictor | Vote share of Benson | Vote share of Haas | Vote share of Humphrey | Vote share of Keough | Vote share of Kingsbury | Vote share of Kroepel |
| Name order | -.00 | -.00 | -.002 | .00 | .00 | .00 |
|  | (.002) | (.00) | (.002) | (.003) | (.00) | (.00) |
| Total number of votes (in 000s) | .01 | -.00 | .00 | -.01 | -.001* | -.001 |
|  | (.01) | (.001) | (.01) | (.01) | (.001) | (.001) |
| Constant | .36*** | .01*** | .28*** | .34*** | .01*** | .01*** |
|  | (.01) | (.001) | (.01) | (.01) | (.000) | (.000) |
|  |  |  |  |  |  |  |
| R^2^ | .004 | .00 | .002 | .001 | .01 | .01 |
| N | 302 | 302 | 302 | 302 | 302 | 302 |

Notes: Cell entries are regression coefficients predicting each candidate’s vote share using the predictors listed in the first column with standard errors in parentheses.

***p<.001 **p<.01 *p<.05 +p<.10

Table S20

*Name Order Effects in the Six-Candidate 2002 Republican Primary for Governor in New Hampshire: Name Order and Name Order Squared*

|  | Dependent Variable | | | | | |
| --- | --- | --- | --- | --- | --- | --- |
| Predictor | Vote share of Benson | Vote share of Haas | Vote share of Humphrey | Vote share of Keough | Vote share of Kingsbury | Vote share of Kroepel |
| Name order | -.00 | -.00 | -.002 | .00 | .00 | .00 |
|  | (.002) | (.00) | (.002) | (.003) | (.00) | (.00) |
| Name order squared | -.001 | .00** | .00 | .00 | .00 | .00*** |
|  | (.001) | (.00) | (.002) | (.002) | (.00) | (.00) |
| Total number of votes (in 000s) | .01 | .00 | .00 | -.01 | -.001* | -.001 |
|  | (.01) | (.001) | (.01) | (.01) | (.001) | (.001) |
| Constant | .36*** | .003** | .28*** | .34*** | .01*** | .003*** |
|  | (.01) | (.001) | (.01) | (.01) | (.001) | (.001) |
|  |  |  |  |  |  |  |
| R^2^ | .01 | .03 | .002 | .001 | .02 | .04 |
| N | 302 | 302 | 302 | 302 | 302 | 302 |

Notes: Cell entries are regression coefficients predicting each candidate’s vote share using the predictors listed in the first column with standard errors in parentheses.

***p<.001 **p<.01 *p<.05 +p<.10

Table S21

*Name Order Effects in the Eight-Candidate 2002 Republican Primary for 1st Congressional District in New Hampshire: First Versus Later*

|  | Dependent Variable | | | | | | | |
| --- | --- | --- | --- | --- | --- | --- | --- | --- |
| Predictor | Vote share of Barrow | Vote share of Bevill | Vote share of Bradley | Vote share of Clark | Vote share of Hoffman | Vote share of Mahoney | Vote share of Stephen | Vote share of Wendelboe |
| Listed first (vs. later) | .01 | .00 | .03 | .04* | .01** | .05+ | .04 | .01 |
|  | (.02) | (.002) | (.03) | (.02) | (.003) | (.02) | (.03) | (.02) |
| Total number of votes (in 000s) | -.01 | .00 | -.04+ | -.002 | .00 | -.02+ | .07*** | -.004 |
|  | (.01) | (.001) | (.02) | (.01) | (.003) | (.01) | (.02) | (.01) |
| Constant | .09*** | .01*** | .35*** | .09*** | .01*** | .21*** | .14*** | .07*** |
|  | (.01) | (.001) | (.02) | (.01) | (.001) | (.01) | (.02) | (.01) |
|  |  |  |  |  |  |  |  |  |
| R^2^ | .01 | .001 | .03 | .04 | .09 | .06 | .12 | .01 |
| N | 114 | 114 | 114 | 114 | 114 | 114 | 114 | 114 |

Notes: Cell entries are regression coefficients predicting each candidate’s vote share using the predictors listed in the first column with standard errors in parentheses.

***p<.001 **p<.01 *p<.05 +p<.10

Table S22

*Name Order Effects in the Eight-Candidate 2002 Republican Primary for 1st Congressional District in New Hampshire: Name Order*

|  | Dependent Variable | | | | | | | |
| --- | --- | --- | --- | --- | --- | --- | --- | --- |
| Predictor | Vote share of Barrow | Vote share of Bevill | Vote share of Bradley | Vote share of Clark | Vote share of Hoffman | Vote share of Mahoney | Vote share of Stephen | Vote share of Wendelboe |
| Name order | -.001 | .00 | -.01 | -.002 | -.001*** | -.004 | -.001 | .00 |
|  | (.002) | (.00) | (.01) | (.002) | (.00) | (.003) | (.004) | (.002) |
| Total number of votes (in 000s) | -.01 | .00 | -.04+ | -.002 | .001 | -.02+ | .07*** | -.004 |
|  | (.01) | (.001) | (.02) | (.01) | (.002) | (.01) | (.02) | (.01) |
| Constant | .09*** | .01*** | .36*** | .10*** | .01*** | .22*** | .14*** | .07*** |
|  | (.01) | (.001) | (.02) | (.01) | (.001) | (.01) | (.02) | (.01) |
|  |  |  |  |  |  |  |  |  |
| R^2^ | .01 | .01 | .04 | .01 | .10 | .05 | .11 | .002 |
| N | 114 | 114 | 114 | 114 | 114 | 114 | 114 | 114 |

Notes: Cell entries are regression coefficients predicting each candidate’s vote share using the predictors listed in the first column with standard errors in parentheses.

***p<.001 **p<.01 *p<.05 +p<.10

Table S23

*Name Order Effects in the Eight-Candidate 2002 Republican Primary for 1st Congressional District in New Hampshire: Name Order and Name Order Squared*

|  | Dependent Variable | | | | | | | |
| --- | --- | --- | --- | --- | --- | --- | --- | --- |
| Predictor | Vote share of Barrow | Vote share of Bevill | Vote share of Bradley | Vote share of Clark | Vote share of Hoffman | Vote share of Mahoney | Vote share of Stephen | Vote share of Wendelboe |
| Name order | -.001 | .00 | -.01 | -.002 | -.001*** | -.004 | -.001 | .00 |
|  | (.002) | (.00) | (.01) | (.002) | (.00) | (.003) | (.004) | (.002) |
| Name order squared | -.001 | -.00 | .001 | .001 | .00 | .003* | .001 | -.00 |
|  | (.001) | (.00) | (.002) | (.001) | (.00) | (.001) | (.002) | (.001) |
| Total number of votes (in 000s) | -.01 | .00 | -.04+ | -.002 | .001 | -.02+ | .07*** | -.004 |
|  | (.01) | (.001) | (.02) | (.01) | (.002) | (.01) | (.02) | (.01) |
| Constant | .10*** | .01*** | .35*** | .09*** | .02*** | .20*** | .14*** | .07*** |
|  | (.01) | (.001) | (.03) | (.01) | (.001) | (.01) | (.02) | (.01) |
|  |  |  |  |  |  |  |  |  |
| R^2^ | .01 | .01 | .04 | .01 | .12 | .10 | .11 | .002 |
| N | 114 | 114 | 114 | 114 | 114 | 114 | 114 | 114 |

Notes: Cell entries are regression coefficients predicting each candidate’s vote share using the predictors listed in the first column with standard errors in parentheses.

***p<.001 **p<.01 *p<.05 +p<.10

Table S24

*Name Order Effects in* *the Four-Candidate 2004 Democratic Primary for 1st Congressional District in New Hampshire: First Versus Later*

|  | Dependent Variable | | | |
| --- | --- | --- | --- | --- |
| Predictor | Vote share of Bruce | Vote share of Duffey | Vote share of Liles | Vote share of Nadeau |
| Listed first (vs. later) | .05* | .06* | .02** | .07+ |
|  | (.02) | (.02) | (.01) | (.04) |
| Total number of votes (in 000s) | -.09 | .12+ | -.04** | .03 |
|  | (.06) | (.06) | (.01) | (.10) |
| Constant | .20*** | .20*** | .04*** | .50*** |
|  | (.02) | (.02) | (.004) | (.03) |
|  |  |  |  |  |
| R^2^ | .06 | .09 | .14 | .03 |
| N | 114 | 114 | 114 | 114 |

Notes: Cell entries are regression coefficients predicting each candidate’s vote share using the predictors listed in the first column with standard errors in parentheses.

***p<.001 **p<.01 *p<.05 +p<.10

Table S25

*Name Order Effects in the Four-Candidate 2004 Democratic Primary for 1st Congressional District in New Hampshire: First Versus Later*

|  | Dependent Variable | | | | | | | |
| --- | --- | --- | --- | --- | --- | --- | --- | --- |
| Predictor | Model 1 Vote share of Bruce | Model 2 Vote share of Bruce | Model 1 Vote share of Duffy | Model 2 Vote share of Duffy | Model 1 Vote share of Liles | Model 2 Vote share of Liles | Model 1 Vote share of Nadeau | Model 2 Vote share of Nadeau |
| Name order | -.01 | -.01 | -.02** | -.02** | -.01*** | -.01*** | -.03** | -.03** |
|  | (.01) | (.01) | (.01) | (.01) | (.001) | (.001) | (.01) | (.01) |
| Name order squared |  | .01* |  | .01 |  | .002 |  | .01 |
|  |  | (.01) |  | (.01) |  | (.002) |  | (.01) |
| Total number of votes (in 000s) | -.09 | -.09 | .12+ | .12+ | -.04** | -.04** | .03 | .03 |
|  | (.06) | (.06) | (.06) | (.06) | (.02) | (.02) | (.10) | (.10) |
| Constant | .21*** | .17*** | .21*** | .19*** | .05*** | .04*** | .52*** | .51*** |
|  | (.02) | (.02) | (.02) | (.02) | (.004) | (.005) | (.03) | (.04) |
|  |  |  |  |  |  |  |  |  |
| R^2^ | .03 | .07 | .10 | .11 | .15 | .17 | .07 | .07 |
| N | 114 | 114 | 114 | 114 | 114 | 114 | 114 | 114 |

Notes: Cell entries are regression coefficients predicting each candidate’s vote share using the predictors listed in the first column with standard errors in parentheses.

***p<.001 **p<.01 *p<.05 +p<.10

Table S26

*Name Order Effects in the 2012 Presidential Election in New Hampshire: First Versus Later*

|  | Dependent Variable | | | |
| --- | --- | --- | --- | --- |
| Predictor | Vote share of the Democratic candidate (Barack Obama)  (1) | Vote share of the Republican candidate (Mitt Romney)  (2) | Vote share of the other candidate (Gary Johnson, Libertarian)  (3) | Vote share of the other candidate (Virgil Goode, Constitution)  (4) |
| Listed first (vs. later) | 0.000 | -0.002 | 0.000 | 0.000 |
|  | (0.006) | (0.006) | (0.001) | (0.000) |
| % Democratic registered voters | 0.608*** | -0.576*** | -0.023*** | -0.010*** |
|  | (0.048) | (0.047) | (0.006) | (0.003) |
| % Republican registered voters | -0.572*** | 0.611*** | -0.031*** | -0.009** |
|  | (0.051) | (0.049) | (0.007) | (0.003) |
| Total number of votes (in 000s) | -0.007*** | 0.007*** | -0.000 | -0.000 |
|  | (0.001) | (0.001) | (0.000) | (0.000) |
| Constant | 0.561*** | 0.406*** | 0.028*** | 0.007*** |
|  | (0.024) | (0.024) | (0.003) | (0.001) |
|  |  |  |  |  |
| R^2^ | 0.725 | 0.736 | 0.083 | 0.072 |
| N | 300 | 300 | 300 | 300 |

Notes: Cell entries are regression coefficients predicting each candidate’s vote share using the predictors listed in the first column with standard errors in parentheses.

***p<.001 **p<.01 *p<.05 +p<.10

Table S27

*Name Order Effects in the 2012 Presidential Election in New Hampshire: Name Order*

|  | Dependent Variable | | | |
| --- | --- | --- | --- | --- |
| Predictor | Vote share of the Democratic candidate (Barack Obama)  (1) | Vote share of the Republican candidate (Mitt Romney)  (2) | Vote share of the other candidate (Gary Johnson, Libertarian)  (3) | Vote share of the other candidate (Virgil Goode, Constitution)  (4) |
| Name order | -0.001 | -0.000 | -0.000 | 0.000 |
|  | (0.003) | (0.003) | (0.000) | (0.000) |
| % Democratic registered voters | 0.608*** | -0.575*** | -0.023*** | -0.010*** |
|  | (0.048) | (0.047) | (0.006) | (0.003) |
| % Republican registered voters | -0.575*** | 0.612*** | -0.030*** | -0.009** |
|  | (0.051) | (0.049) | (0.007) | (0.003) |
| Total number of votes (in 000s) | -0.007*** | 0.007*** | -0.000 | -0.000 |
|  | (0.001) | (0.001) | (0.000) | (0.000) |
| Constant | 0.562*** | 0.404*** | 0.028*** | 0.007*** |
|  | (0.024) | (0.023) | (0.003) | (0.001) |
|  |  |  |  |  |
| R^2^ | 0.725 | 0.736 | 0.084 | 0.072 |
| N | 300 | 300 | 300 | 300 |

Notes: Cell entries are regression coefficients predicting each candidate’s vote share using the predictors listed in the first column with standard errors in parentheses.

***p<.001 **p<.01 *p<.05 +p<.10

Table S28

*Name Order Effects in the 2012 Presidential Election in New Hampshire: Name Order and Name Order Squared*

|  | Dependent Variable | | | |
| --- | --- | --- | --- | --- |
| Predictor | Vote share of the Democratic candidate (Barack Obama)  (1) | Vote share of the Republican candidate (Mitt Romney)  (2) | Vote share of the other candidate (Gary Johnson, Libertarian)  (3) | Vote share of the other candidate (Virgil Goode, Constitution)  (4) |
| Name order | -0.001 | -0.000 | -0.000 | -0.000 |
|  | (0.003) | (0.003) | (0.000) | (0.000) |
| Name order squared | -0.004 | -0.005 | -0.001 | -0.001 |
|  | (0.006) | (0.006) | (0.001) | (0.001) |
| % Democratic registered voters | 0.609*** | -0.576*** | -0.023*** | -0.023*** |
|  | (0.048) | (0.047) | (0.006) | (0.006) |
| % Republican registered voters | -0.575*** | 0.610*** | -0.030*** | -0.030*** |
|  | (0.051) | (0.049) | (0.007) | (0.007) |
| Total number of votes (in 000s) | -0.007*** | 0.007*** | -0.000 | -0.000 |
|  | (0.001) | (0.001) | (0.000) | (0.000) |
| Constant | 0.564*** | 0.408*** | 0.028*** | 0.028*** |
|  | (0.024) | (0.024) | (0.003) | (0.003) |
|  |  |  |  |  |
| R^2^ | 0.725 | 0.736 | 0.087 | 0.087 |
| N | 300 | 300 | 300 | 300 |

Notes: Cell entries are regression coefficients predicting each candidate’s vote share using the predictors listed in the first column with standard errors in parentheses.

***p<.001 **p<.01 *p<.05 +p<.10

Table S29

*Name Order Effects in the 2012 Gubernatorial Election in New Hampshire: First Versus Later*

|  | Dependent Variable | | |
| --- | --- | --- | --- |
| Predictor | Vote share of the Democratic candidate (Maggie Hassan)  (1) | Vote share of the Republican candidate (Ovide Lamontagne)  (2) | Vote share of the other candidate (John J. Babiarz, Libertarian)  (3) |
| Listed first (vs. later) | 0.002 | 0.006 | -0.000 |
|  | (0.006) | (0.006) | (0.002) |
| % Democratic registered voters | 0.701*** | -0.675*** | -0.026* |
|  | (0.046) | (0.047) | (0.013) |
| % Republican registered voters | -0.478*** | 0.528*** | -0.050*** |
|  | (0.049) | (0.049) | (0.014) |
| Total number of votes (in 000s) | -0.005*** | 0.005*** | -0.000 |
|  | (0.001) | (0.001) | (0.000) |
| Constant | 0.521*** | 0.425*** | 0.051*** |
|  | (0.023) | (0.024) | (0.007) |
|  |  |  |  |
| R^2^ | 0.738 | 0.740 | 0.048 |
| N | 300 | 300 | 300 |

Notes: Cell entries are regression coefficients predicting each candidate’s vote share using the predictors listed in the first column with standard errors in parentheses.

***p<.001 **p<.01 *p<.05 +p<.10

Table S30

*Name Order Effects in the 2012 Gubernatorial Election in New Hampshire: Name Order*

|  | Dependent Variable | | |
| --- | --- | --- | --- |
| Predictor | Vote share of the Democratic candidate (Maggie Hassan)  (1) | Vote share of the Republican candidate (Ovide Lamontagne)  (2) | Vote share of the other candidate (John J. Babiarz, Libertarian)  (3) |
| Name order | -0.002 | -0.004 | -0.002+ |
|  | (0.003) | (0.003) | (0.001) |
| % Democratic registered voters | 0.701*** | -0.675*** | -0.026* |
|  | (0.046) | (0.047) | (0.013) |
| % Republican registered voters | -0.480*** | 0.528*** | -0.047** |
|  | (0.049) | (0.049) | (0.014) |
| Total number of votes (in 000s) | -0.005*** | 0.005*** | -0.000 |
|  | (0.001) | (0.001) | (0.000) |
| Constant | 0.522*** | 0.427*** | 0.050*** |
|  | (0.023) | (0.023) | (0.007) |
|  |  |  |  |
| R^2^ | 0.738 | 0.740 | 0.057 |
| N | 300 | 300 | 300 |

Notes: Cell entries are regression coefficients predicting each candidate’s vote share using the predictors listed in the first column with standard errors in parentheses.

***p<.001 **p<.01 *p<.05 +p<.10

Table S31

*Name Order Effects in the 2012 Gubernatorial Election in New Hampshire: Name Order and Name Order Squared*

|  | Dependent Variable | | |
| --- | --- | --- | --- |
| Predictor | Vote share of the Democratic candidate (Maggie Hassan)  (1) | Vote share of the Republican candidate (Ovide Lamontagne)  (2) | Vote share of the other candidate (John J. Babiarz, Libertarian)  (3) |
| Name order | -0.002 | -0.004 | -0.001 |
|  | (0.003) | (0.003) | (0.001) |
| Name order squared | -0.002 | 0.001 | -0.005** |
|  | (0.006) | (0.006) | (0.002) |
| % Democratic registered voters | 0.701*** | -0.675*** | -0.026* |
|  | (0.046) | (0.047) | (0.013) |
| % Republican registered voters | -0.480*** | 0.528*** | -0.046** |
|  | (0.049) | (0.049) | (0.014) |
| Total number of votes (in 000s) | -0.005*** | 0.005*** | -0.000 |
|  | (0.001) | (0.001) | (0.000) |
| Constant | 0.521*** | 0.437*** | 0.036*** |
|  | (0.031) | (0.030) | (0.009) |
|  |  |  |  |
| R^2^ | 0.738 | 0.740 | 0.086 |
| N | 300 | 300 | 300 |

Notes: Cell entries are regression coefficients predicting each candidate’s vote share using the predictors listed in the first column with standard errors in parentheses.

***p<.001 **p<.01 *p<.05 +p<.10

Table S32

*Name Order Effects in the 2012 Congressional District 1 Election in New Hampshire: First Versus Later*

|  | Dependent Variable | | |
| --- | --- | --- | --- |
| Predictor | Vote share of the Democratic candidate (Carol Shea-Porter)  (1) | Vote share of the Republican candidate (Frank C. Guinta)  (2) | Vote share of the other candidate (Brendan Kelly, Libertarian)  (3) |
| Listed first (vs. later) | -0.003 | -0.009 | -0.001 |
|  | (0.008) | (0.008) | (0.003) |
| % Democratic registered voters | 0.457*** | -0.409*** | -0.041 |
|  | (0.075) | (0.069) | (0.027) |
| % Republican registered voters | -0.643*** | 0.696*** | -0.053* |
|  | (0.075) | (0.068) | (0.027) |
| Total number of votes (in 000s) | -0.002 | 0.002 | 0.000 |
|  | (0.001) | (0.001) | (0.001) |
| Constant | 0.590*** | 0.344*** | 0.069*** |
|  | (0.040) | (0.036) | (0.014) |
|  |  |  |  |
| R^2^ | 0.780 | 0.817 | 0.037 |
| N | 114 | 114 | 114 |

Notes: Cell entries are regression coefficients predicting each candidate’s vote share using the predictors listed in the first column with standard errors in parentheses.

***p<.001 **p<.01 *p<.05 +p<.10

Table S33

*Name Order Effects in the 2012 Congressional District 1 Election in New Hampshire: Name Order*

|  | Dependent Variable | | |
| --- | --- | --- | --- |
| Predictor | Vote share of the Democratic candidate (Carol Shea-Porter)  (1) | Vote share of the Republican candidate (Frank C. Guinta)  (2) | Vote share of the other candidate (Brendan Kelly, Libertarian)  (3) |
| Name order | -0.002 | 0.001 | -0.003 |
|  | (0.005) | (0.005) | (0.002) |
| % Democratic registered voters | 0.460*** | -0.416*** | -0.039 |
|  | (0.075) | (0.069) | (0.026) |
| % Republican registered voters | -0.649*** | 0.697*** | -0.049+ |
|  | (0.075) | (0.068) | (0.026) |
| Total number of votes (in 000s) | -0.002 | 0.002 | 0.000 |
|  | (0.001) | (0.001) | (0.001) |
| Constant | 0.590*** | 0.342*** | 0.067*** |
|  | (0.040) | (0.036) | (0.014) |
|  |  |  |  |
| R^2^ | 0.780 | 0.815 | 0.057 |
| N | 114 | 114 | 114 |

Notes: Cell entries are regression coefficients predicting each candidate’s vote share using the predictors listed in the first column with standard errors in parentheses.

***p<.001 **p<.01 *p<.05 +p<.10

Table S34

*Name Order Effects in the 2012 Congressional District 1 Election in New Hampshire: Name Order and Name Order Squared*

|  | Dependent Variable | | |
| --- | --- | --- | --- |
| Predictor | Vote share of the Democratic candidate (Carol Shea-Porter)  (1) | Vote share of the Republican candidate (Frank C. Guinta)  (2) | Vote share of the other candidate (Brendan Kelly, Libertarian)  (3) |
| Name order | -0.002 | 0.001 | -0.002 |
|  | (0.005) | (0.005) | (0.002) |
| Name order squared | -0.012 | -0.012+ | -0.011*** |
|  | (0.008) | (0.007) | (0.003) |
| % Democratic registered voters | 0.453*** | -0.409*** | -0.039 |
|  | (0.075) | (0.068) | (0.025) |
| % Republican registered voters | -0.650*** | 0.697*** | -0.048+ |
|  | (0.075) | (0.068) | (0.025) |
| Total number of votes (in 000s) | -0.003+ | 0.002 | 0.000 |
|  | (0.001) | (0.001) | (0.000) |
| Constant | 0.602*** | 0.348*** | 0.074*** |
|  | (0.040) | (0.036) | (0.013) |
|  |  |  |  |
| R^2^ | 0.784 | 0.819 | 0.180 |
| N | 114 | 114 | 114 |

Notes: Cell entries are regression coefficients predicting each candidate’s vote share using the predictors listed in the first column with standard errors in parentheses.

***p<.001 **p<.01 *p<.05 +p<.10

Table S35

*Name Order Effects in the 2012 Congressional District 2 Election in New Hampshire: First Versus Later*

|  | Dependent Variable | | |
| --- | --- | --- | --- |
| Predictor | Vote share of the Democratic candidate (Ann McLane Kuster)  (1) | Vote share of the Republican candidate (Charles Bass)  (2) | Vote share of the other candidate (Hardy Macia, Libertarian)  (3) |
| Listed first (vs. later) | 0.011+ | 0.008 | 0.000 |
|  | (0.006) | (0.006) | (0.003) |
| % Democratic registered voters | 0.803*** | -0.721*** | -0.083*** |
|  | (0.049) | (0.047) | (0.021) |
| % Republican registered voters | -0.517*** | 0.567*** | -0.058* |
|  | (0.053) | (0.050) | (0.023) |
| Total number of votes (in 000s) | -0.006*** | 0.006*** | 0.001 |
|  | (0.002) | (0.002) | (0.001) |
| Constant | 0.454*** | 0.460*** | 0.081*** |
|  | (0.024) | (0.023) | (0.011) |
|  |  |  |  |
| R^2^ | 0.826 | 0.831 | 0.078 |
| N | 186 | 186 | 186 |

Notes: Cell entries are regression coefficients predicting each candidate’s vote share using the predictors listed in the first column with standard errors in parentheses.

***p<.001 **p<.01 *p<.05 +p<.10

Table S36

*Name Order Effects in the 2012 Congressional District 2 Election in New Hampshire: Name Order*

|  | Dependent Variable | | |
| --- | --- | --- | --- |
| Predictor | Vote share of the Democratic candidate (Ann McLane Kuster)  (1) | Vote share of the Republican candidate (Charles Bass)  (2) | Vote share of the other candidate (Hardy Macia, Libertarian)  (3) |
| Name order | -0.005 | -0.003 | -0.005** |
|  | (0.004) | (0.003) | (0.002) |
| % Democratic registered voters | 0.805*** | -0.724*** | -0.083*** |
|  | (0.049) | (0.047) | (0.021) |
| % Republican registered voters | -0.518*** | 0.566*** | -0.049* |
|  | (0.053) | (0.050) | (0.023) |
| Total number of votes (in 000s) | -0.006*** | 0.006*** | 0.001 |
|  | (0.002) | (0.002) | (0.001) |
| Constant | 0.458*** | 0.464*** | 0.079*** |
|  | (0.024) | (0.023) | (0.010) |
|  |  |  |  |
| R^2^ | 0.825 | 0.830 | 0.127 |
| N | 186 | 186 | 186 |

Notes: Cell entries are regression coefficients predicting each candidate’s vote share using the predictors listed in the first column with standard errors in parentheses.

***p<.001 **p<.01 *p<.05 +p<.10

Table S37

*Name Order Effects in the 2012 Congressional District 2 Election in New Hampshire: Name Order and Name Order Squared*

|  | Dependent Variable | | |
| --- | --- | --- | --- |
| Predictor | Vote share of the Democratic candidate (Ann McLane Kuster)  (1) | Vote share of the Republican candidate (Charles Bass)  (2) | Vote share of the other candidate (Hardy Macia, Libertarian)  (3) |
| Name order | -0.005 | -0.002 | -0.005** |
|  | (0.004) | (0.003) | (0.001) |
| Name order squared | 0.006 | 0.008 | -0.013*** |
|  | (0.006) | (0.006) | (0.002) |
| % Democratic registered voters | 0.803*** | -0.721*** | -0.081*** |
|  | (0.049) | (0.047) | (0.019) |
| % Republican registered voters | -0.517*** | 0.568*** | -0.043* |
|  | (0.053) | (0.050) | (0.021) |
| Total number of votes (in 000s) | -0.006*** | 0.006*** | 0.000 |
|  | (0.002) | (0.002) | (0.001) |
| Constant | 0.454*** | 0.458*** | 0.086*** |
|  | (0.024) | (0.023) | (0.010) |
|  |  |  |  |
| R^2^ | 0.826 | 0.831 | 0.247 |
| N | 186 | 186 | 186 |

Notes: Cell entries are regression coefficients predicting each candidate’s vote share using the predictors listed in the first column with standard errors in parentheses.

***p<.001 **p<.01 *p<.05 +p<.10

Table S38

*Name Order Effects in the 2016 Presidential Election in New Hampshire: First Versus Later*

| Predictor | Vote share of the Democratic candidate (Hillary Clinton)  (1) | Vote share of the Republican candidate (Donald J. Trump)  (2) | Vote share of the other candidate (Gary Johnson, Libertarian)  (3) | Vote share of the other candidate (Jill Stein, Green)  (4) | Vote share of the other candidate (Roque “Rocky” De La Fuente, American Delta)  (5) | Vote share of the Democratic (Hillary Clinton) or Republican (Donald J. Trump) candidate  (6) | Vote share of the Democratic (Hillary Clinton) or Republican (Donald J. Trump) candidate  (7) |
| --- | --- | --- | --- | --- | --- | --- | --- |
| Listed first (vs. later) | 0.015+ | 0.017* | -0.001 | 0.001+ | 0.000+ | 0.015+ | 0.016* |
|  | (0.008) | (0.008) | (0.002) | (0.001) | (0.000) | (0.008) | (0.007) |
| % Democratic registered voters | 0.441*** | -0.470*** | 0.016 | 0.010* | -0.001 | 0.441** | 0.441** |
|  | (0.059) | (0.058) | (0.011) | (0.004) | (0.002) | (0.142) | (0.142) |
| % Republican registered voters | -0.654*** | 0.681*** | 0.002 | -0.017** | -0.003 | -0.654*** | -0.654*** |
|  | (0.069) | (0.068) | (0.013) | (0.005) | (0.002) | (0.094) | (0.093) |
| Total number of votes (in 000s) | 0.004* | -0.003* | 0.000 | -0.000** | -0.000 | 0.004+ | 0.004+ |
|  | (0.002) | (0.002) | (0.000) | (0.000) | (0.000) | (0.002) | (0.002) |
| Is Republican |  |  |  |  |  | -0.126 | -0.125 |
|  |  |  |  |  |  | (0.119) | (0.119) |
| Is Republican × Listed first |  |  |  |  |  | 0.002 |  |
|  |  |  |  |  |  | (0.008) |  |
| Is Republican × % Democratic registered voters |  |  |  |  |  | -0.911*** | -0.911*** |
|  |  |  |  |  |  | (0.274) | (0.274) |
| Is Republican × % Republican registered voters |  |  |  |  |  | 1.335*** | 1.335*** |
|  |  |  |  |  |  | (0.187) | (0.187) |
| Is Republican × Total number of votes (in 000s) |  |  |  |  |  | -0.007+ | -0.007+ |
|  |  |  |  |  |  | (0.004) | (0.004) |
| Constant | 0.531*** | 0.405*** | 0.037*** | 0.013*** | 0.002* | 0.531*** | 0.530*** |
|  | (0.034) | (0.033) | (0.006) | (0.003) | (0.001) | (0.059) | (0.059) |
|  |  |  |  |  |  |  |  |
| R^2^ | 0.581 | 0.607 | 0.013 | 0.156 | 0.022 | 0.595 | 0.595 |
| N | 300 | 300 | 300 | 300 | 300 | 600 | 600 |

Notes: Cell entries are regression coefficients predicting each candidate’s vote share using the predictors listed in the first column with standard errors in parentheses. Standard errors in (6)-(7) were clustered by voting places.

***p<.001 **p<.01 *p<.05 +p<.10

Table S39

*Name Order Effects in the 2016 Presidential Election in New Hampshire: Name Order*

|  | Dependent Variable | | | | |
| --- | --- | --- | --- | --- | --- |
| Predictor | Vote share of the Democratic candidate (Hillary Clinton)  (1) | Vote share of the Republican candidate (Donald J. Trump)  (2) | Vote share of the other candidate (Gary Johnson, Libertarian)  (3) | Vote share of the other candidate (Jill Stein, Green)  (4) | Vote share of the other candidate (Roque “Rocky” De La Fuente, American Delta)  (5) |
| Name order | -0.010* | -0.009+ | 0.000 | -0.001+ | -0.000+ |
|  | (0.005) | (0.005) | (0.001) | (0.000) | (0.000) |
| % Democratic registered voters | 0.444*** | -0.466*** | 0.017 | 0.009* | -0.001 |
|  | (0.059) | (0.058) | (0.011) | (0.004) | (0.002) |
| % Republican registered voters | -0.652*** | 0.686*** | 0.002 | -0.017** | -0.003 |
|  | (0.069) | (0.068) | (0.013) | (0.005) | (0.002) |
| Total number of votes (in 000s) | 0.004* | -0.003* | 0.000 | -0.000** | -0.000 |
|  | (0.002) | (0.002) | (0.000) | (0.000) | (0.000) |
| Constant | 0.533*** | 0.408*** | 0.037*** | 0.014*** | 0.003* |
|  | (0.034) | (0.033) | (0.006) | (0.003) | (0.001) |
|  |  |  |  |  |  |
| R^2^ | 0.582 | 0.607 | 0.013 | 0.154 | 0.021 |
| N | 300 | 300 | 300 | 300 | 300 |

Notes: Cell entries are regression coefficients predicting each candidate’s vote share using the predictors listed in the first column with standard errors in parentheses.

***p<.001 **p<.01 *p<.05 +p<.10

Table S40

*Name Order Effects in the 2016 Presidential Election in New Hampshire: Name Order and Name Order Squared*

|  | Dependent Variable | | | | |
| --- | --- | --- | --- | --- | --- |
| Predictor | Vote share of the Democratic candidate (Hillary Clinton)  (1) | Vote share of the Republican candidate (Donald J. Trump)  (2) | Vote share of the other candidate (Gary Johnson, Libertarian)  (3) | Vote share of the other candidate (Jill Stein, Green)  (4) | Vote share of the other candidate (Roque “Rocky” De La Fuente, American Delta)  (5) |
| Name order | -0.010+ | -0.009+ | 0.000 | -0.001+ | -0.000+ |
|  | (0.005) | (0.005) | (0.001) | (0.000) | (0.000) |
| Name order squared | 0.002 | 0.005 | -0.001 | 0.000 | 0.000 |
|  | (0.008) | (0.008) | (0.002) | (0.001) | (0.000) |
| % Democratic registered voters | 0.444*** | -0.469*** | 0.016 | 0.010* | -0.001 |
|  | (0.059) | (0.058) | (0.011) | (0.005) | (0.002) |
| % Republican registered voters | -0.652*** | 0.684*** | 0.001 | -0.017** | -0.003 |
|  | (0.069) | (0.068) | (0.013) | (0.005) | (0.002) |
| Total number of votes (in 000s) | 0.004* | -0.003* | 0.000 | -0.000** | -0.000 |
|  | (0.002) | (0.002) | (0.000) | (0.000) | (0.000) |
| Constant | 0.533*** | 0.406*** | 0.037*** | 0.013*** | 0.002* |
|  | (0.034) | (0.033) | (0.006) | (0.003) | (0.001) |
|  |  |  |  |  |  |
| R^2^ | 0.582 | 0.607 | 0.013 | 0.156 | 0.022 |
| N | 300 | 300 | 300 | 300 | 300 |

Notes: Cell entries are regression coefficients predicting each candidate’s vote share using the predictors listed in the first column with standard errors in parentheses.

***p<.001 **p<.01 *p<.05 +p<.10

Table S41

*Name Order Effects in the 2016 U.S. Senate Election in New Hampshire: First Versus Later*

|  | Dependent Variable | | | |
| --- | --- | --- | --- | --- |
| Predictor | Vote share of the Democratic candidate (Maggie Hassan)  (1) | Vote share of the Republican candidate (Kelly Ayotte)  (2) | Vote share of the other candidate (Brian Chabot, Libertarian)  (3) | Vote share of the other candidate (Aaron Day, Independent)  (4) |
| Listed first (vs. later) | 0.015* | 0.012* | -0.000 | 0.000 |
|  | (0.006) | (0.005) | (0.001) | (0.001) |
| % Democratic registered voters | 0.286*** | -0.280*** | 0.003 | -0.012 |
|  | (0.042) | (0.037) | (0.006) | (0.010) |
| % Republican registered voters | -0.781*** | 0.795*** | -0.013+ | 0.006 |
|  | (0.049) | (0.043) | (0.007) | (0.012) |
| Total number of votes (in 000s) | 0.001 | -0.001 | 0.000 | -0.000 |
|  | (0.001) | (0.001) | (0.000) | (0.000) |
| Constant | 0.630*** | 0.312*** | 0.020*** | 0.027*** |
|  | (0.024) | (0.021) | (0.004) | (0.006) |
|  |  |  |  |  |
| R^2^ | 0.717 | 0.764 | 0.027 | 0.017 |
| N | 300 | 300 | 300 | 300 |

Notes: Cell entries are regression coefficients predicting each candidate’s vote share using the predictors listed in the first column with standard errors in parentheses.

***p<.001 **p<.01 *p<.05 +p<.10

Table S42

*Name Order Effects in the 2016 U.S. Senate Election in New Hampshire: Name Order*

|  | Dependent Variable | | | |
| --- | --- | --- | --- | --- |
| Predictor | Vote share of the Democratic candidate (Maggie Hassan)  (1) | Vote share of the Republican candidate (Kelly Ayotte)  (2) | Vote share of the other candidate (Brian Chabot, Libertarian)  (3) | Vote share of the other candidate (Aaron Day, Independent)  (4) |
| Name order | -0.010** | -0.005 | -0.000 | -0.001 |
|  | (0.004) | (0.003) | (0.001) | (0.001) |
| % Democratic registered voters | 0.289*** | -0.277*** | 0.003 | -0.012 |
|  | (0.042) | (0.037) | (0.006) | (0.010) |
| % Republican registered voters | -0.779*** | 0.796*** | -0.013+ | 0.004 |
|  | (0.049) | (0.044) | (0.007) | (0.012) |
| Total number of votes (in 000s) | 0.001 | -0.001 | 0.000 | -0.000 |
|  | (0.001) | (0.001) | (0.000) | (0.000) |
| Constant | 0.633*** | 0.315*** | 0.020*** | 0.027*** |
|  | (0.024) | (0.021) | (0.004) | (0.006) |
|  |  |  |  |  |
| R^2^ | 0.718 | 0.762 | 0.028 | 0.022 |
| N | 300 | 300 | 300 | 300 |

Notes: Cell entries are regression coefficients predicting each candidate’s vote share using the predictors listed in the first column with standard errors in parentheses.

***p<.001 **p<.01 *p<.05 +p<.10

Table S43

*Name Order Effects in the 2016 U.S. Senate Election in New Hampshire: Name Order and Name Order Squared*

|  | Dependent Variable | | | |
| --- | --- | --- | --- | --- |
| Predictor | Vote share of the Democratic candidate (Maggie Hassan)  (1) | Vote share of the Republican candidate (Kelly Ayotte)  (2) | Vote share of the other candidate (Brian Chabot, Libertarian)  (3) | Vote share of the other candidate (Aaron Day, Independent)  (4) |
| Name order | -0.010** | -0.005 | -0.000 | -0.001 |
|  | (0.004) | (0.003) | (0.001) | (0.001) |
| Name order squared | 0.002 | 0.009+ | -0.001 | -0.002+ |
|  | (0.006) | (0.005) | (0.001) | (0.001) |
| % Democratic registered voters | 0.289*** | -0.282*** | 0.002 | -0.014 |
|  | (0.042) | (0.037) | (0.006) | (0.010) |
| % Republican registered voters | -0.779*** | 0.793*** | -0.014* | 0.003 |
|  | (0.049) | (0.044) | (0.007) | (0.012) |
| Total number of votes (in 000s) | 0.001 | -0.001 | 0.000 | -0.000 |
|  | (0.001) | (0.001) | (0.000) | (0.000) |
| Constant | 0.633*** | 0.311*** | 0.022*** | 0.030*** |
|  | (0.024) | (0.021) | (0.004) | (0.006) |
|  |  |  |  |  |
| R^2^ | 0.718 | 0.765 | 0.036 | 0.031 |
| N | 300 | 300 | 300 | 300 |

Notes: Cell entries are regression coefficients predicting each candidate’s vote share using the predictors listed in the first column with standard errors in parentheses.

***p<.001 **p<.01 *p<.05 +p<.10

Table S44

*Name Order Effects in the 2016 Gubernatorial Election in New Hampshire: First Versus Later*

|  | Dependent Variable | | |
| --- | --- | --- | --- |
| Predictor | Vote share of the Democratic candidate (Colin Van Ostern)  (1) | Vote share of the Republican candidate (Chris Sununu)  (2) | Vote share of the other candidate (Max Abramson, Libertarian)  (3) |
| Listed first (vs. later) | 0.014+ | 0.016* | -0.000 |
|  | (0.008) | (0.008) | (0.002) |
| % Democratic registered voters | 0.337*** | -0.315*** | -0.026+ |
|  | (0.056) | (0.053) | (0.014) |
| % Republican registered voters | -0.649*** | 0.710*** | -0.051*** |
|  | (0.066) | (0.061) | (0.016) |
| Total number of votes (in 000s) | -0.001 | 0.001 | -0.000 |
|  | (0.002) | (0.001) | (0.000) |
| Constant | 0.574*** | 0.348*** | 0.067*** |
|  | (0.032) | (0.030) | (0.008) |
|  |  |  |  |
| R^2^ | 0.549 | 0.595 | 0.036 |
| N | 300 | 300 | 300 |

Notes: Cell entries are regression coefficients predicting each candidate’s vote share using the predictors listed in the first column with standard errors in parentheses.

***p<.01 **p<.05 *p<.10

Table S45

*Name Order Effects in the 2016 Gubernatorial Election in New Hampshire: Name Order*

|  | Dependent Variable | | |
| --- | --- | --- | --- |
| Predictor | Vote share of the Democratic candidate (Colin Van Ostern)  (1) | Vote share of the Republican candidate (Chris Sununu)  (2) | Vote share of the other candidate (Max Abramson, Libertarian)  (3) |
| Name order | -0.009+ | -0.010* | -0.003* |
|  | (0.005) | (0.004) | (0.001) |
| % Democratic registered voters | 0.339*** | -0.311*** | -0.026+ |
|  | (0.056) | (0.053) | (0.013) |
| % Republican registered voters | -0.647*** | 0.716*** | -0.056*** |
|  | (0.066) | (0.062) | (0.016) |
| Total number of votes (in 000s) | -0.001 | 0.001 | -0.000 |
|  | (0.002) | (0.001) | (0.000) |
| Constant | 0.577*** | 0.350*** | 0.068*** |
|  | (0.032) | (0.030) | (0.008) |
|  |  |  |  |
| R^2^ | 0.549 | 0.596 | 0.055 |
| N | 300 | 300 | 300 |

Notes: Cell entries are regression coefficients predicting each candidate’s vote share using the predictors listed in the first column with standard errors in parentheses.

***p<.001 **p<.01 *p<.05 +p<.10

Table S46

*Name Order Effects in the 2016 Gubernatorial Election in New Hampshire: Name Order and Name Order Squared*

|  | Dependent Variable | | |
| --- | --- | --- | --- |
| Predictor | Vote share of the Democratic candidate (Colin Van Ostern)  (1) | Vote share of the Republican candidate (Chris Sununu)  (2) | Vote share of the other candidate (Max Abramson, Libertarian)  (3) |
| Name order | -0.009+ | -0.010* | -0.003* |
|  | (0.005) | (0.004) | (0.001) |
| Name order squared | 0.002 | 0.002 | -0.009*** |
|  | (0.008) | (0.007) | (0.002) |
| % Democratic registered voters | 0.339*** | -0.312*** | -0.032* |
|  | (0.057) | (0.053) | (0.013) |
| % Republican registered voters | -0.647*** | 0.715*** | -0.062*** |
|  | (0.066) | (0.062) | (0.015) |
| Total number of votes (in 000s) | -0.001 | 0.001 | -0.000 |
|  | (0.002) | (0.001) | (0.000) |
| Constant | 0.575*** | 0.349*** | 0.078*** |
|  | (0.032) | (0.030) | (0.008) |
|  |  |  |  |
| R^2^ | 0.549 | 0.596 | 0.116 |
| N | 300 | 300 | 300 |

Notes: Cell entries are regression coefficients predicting each candidate’s vote share using the predictors listed in the first column with standard errors in parentheses.

***p<.001 **p<.01 *p<.05 +p<.10

Table S47

*Name Order Effects in the 2016 Congressional District 1 Election in New Hampshire: First Versus Later*

|  | Dependent Variable | | | | |
| --- | --- | --- | --- | --- | --- |
| Predictor | Vote share of the Democratic candidate (Carol Shea-Porter)  (1) | Vote share of the Republican candidate (Frank Guinta)  (2) | Vote share of the other candidate (Robert Lombardo, Libertarian)  (3) | Vote share of the other candidate (Brendan Kelly, Independent)  (4) | Vote share of the other candidate (Shawn P. O’Connor, Independent)  (5) |
| Listed first (vs. later) | 0.007 | 0.005 | 0.001 | -0.000 | -0.001 |
|  | (0.007) | (0.007) | (0.001) | (0.001) | (0.004) |
| % Democratic registered voters | 0.557*** | -0.495*** | -0.002 | -0.020+ | -0.043 |
|  | (0.072) | (0.068) | (0.010) | (0.011) | (0.040) |
| % Republican registered voters | -0.557*** | 0.630*** | -0.015 | -0.013 | -0.038 |
|  | (0.069) | (0.066) | (0.010) | (0.011) | (0.039) |
| Total number of votes (in 000s) | 0.000 | 0.000 | 0.000 | 0.000 | -0.001 |
|  | (0.001) | (0.001) | (0.000) | (0.000) | (0.001) |
| Constant | 0.466*** | 0.363*** | 0.018** | 0.025*** | 0.123*** |
|  | (0.038) | (0.037) | (0.006) | (0.006) | (0.021) |
|  |  |  |  |  |  |
| R^2^ | 0.816 | 0.833 | 0.048 | 0.034 | 0.021 |
| N | 114 | 114 | 114 | 114 | 114 |

Notes: Cell entries are regression coefficients predicting each candidate’s vote share using the predictors listed in the first column with standard errors in parentheses.

***p<.001 **p<.01 *p<.05 +p<.10

Table S48

*Name Order Effects in the 2016 Congressional District 1 Election in New Hampshire: Name Order*

|  | Dependent Variable | | | | |
| --- | --- | --- | --- | --- | --- |
| Predictor | Vote share of the Democratic candidate (Carol Shea-Porter)  (1) | Vote share of the Republican candidate (Frank Guinta)  (2) | Vote share of the other candidate (Robert Lombardo, Libertarian)  (3) | Vote share of the other candidate (Brendan Kelly, Independent)  (4) | Vote share of the other candidate (Shawn P. O’Connor, Independent)  (5) |
| Name order | -0.004 | -0.002 | -0.001+ | -0.001 | -0.004+ |
|  | (0.004) | (0.004) | (0.001) | (0.001) | (0.002) |
| % Democratic registered voters | 0.558*** | -0.495*** | -0.002 | -0.020+ | -0.043 |
|  | (0.072) | (0.068) | (0.010) | (0.011) | (0.039) |
| % Republican registered voters | -0.558*** | 0.630*** | -0.016 | -0.015 | -0.050 |
|  | (0.069) | (0.066) | (0.010) | (0.011) | (0.038) |
| Total number of votes (in 000s) | 0.000 | 0.000 | 0.000 | 0.000 | -0.001 |
|  | (0.001) | (0.001) | (0.000) | (0.000) | (0.001) |
| Constant | 0.468*** | 0.364*** | 0.019*** | 0.026*** | 0.127*** |
|  | (0.038) | (0.037) | (0.006) | (0.006) | (0.021) |
|  |  |  |  |  |  |
| R^2^ | 0.816 | 0.833 | 0.061 | 0.043 | 0.049 |
| N | 114 | 114 | 114 | 114 | 114 |

Notes: Cell entries are regression coefficients predicting each candidate’s vote share using the predictors listed in the first column with standard errors in parentheses.

***p<.001 **p<.01 *p<.05 +p<.10

Table S49

*Name Order Effects in the 2016 Congressional District 1 Election in New Hampshire: Name Order and Name Order Squared*

|  | Dependent Variable | | | | |
| --- | --- | --- | --- | --- | --- |
| Predictor | Vote share of the Democratic candidate (Carol Shea-Porter)  (1) | Vote share of the Republican candidate (Frank Guinta)  (2) | Vote share of the other candidate (Robert Lombardo, Libertarian)  (3) | Vote share of the other candidate (Brendan Kelly, Independent)  (4) | Vote share of the other candidate (Shawn P. O’Connor, Independent)  (5) |
| Name order | -0.004 | -0.002 | -0.001+ | -0.001 | -0.004+ |
|  | (0.004) | (0.004) | (0.001) | (0.001) | (0.002) |
| Name order squared | 0.003 | 0.002 | -0.001 | -0.003* | -0.014*** |
|  | (0.007) | (0.007) | (0.001) | (0.001) | (0.004) |
| % Democratic registered voters | 0.557*** | -0.495*** | -0.002 | -0.020+ | -0.046 |
|  | (0.072) | (0.068) | (0.010) | (0.011) | (0.037) |
| % Republican registered voters | -0.558*** | 0.630*** | -0.015 | -0.014 | -0.047 |
|  | (0.069) | (0.066) | (0.010) | (0.011) | (0.036) |
| Total number of votes (in 000s) | 0.000 | 0.000 | 0.000 | 0.000 | -0.001 |
|  | (0.001) | (0.001) | (0.000) | (0.000) | (0.001) |
| Constant | 0.466*** | 0.363*** | 0.020*** | 0.028*** | 0.137*** |
|  | (0.039) | (0.037) | (0.006) | (0.006) | (0.020) |
|  |  |  |  |  |  |
| R^2^ | 0.816 | 0.833 | 0.065 | 0.094 | 0.154 |
| N | 114 | 114 | 114 | 114 | 114 |

Notes: Cell entries are regression coefficients predicting each candidate’s vote share using the predictors listed in the first column with standard errors in parentheses.

***p<.001 **p<.01 *p<.05 +p<.10

Table S50

*Name Order Effects in the 2016 Congressional District 2 Election in New Hampshire: First Versus Later*

|  | Dependent Variable | | |
| --- | --- | --- | --- |
| Predictor | Vote share of the Democratic candidate (Ann McLane Kuster)  (1) | Vote share of the Republican candidate (Jim Lawrence)  (2) | Vote share of the other candidate (John J. Babiarz, Independent)  (3) |
| Listed first (vs. later) | 0.015 | 0.021** | -0.000 |
|  | (0.009) | (0.008) | (0.003) |
| % Democratic registered voters | 0.346*** | -0.337*** | -0.016 |
|  | (0.056) | (0.046) | (0.019) |
| % Republican registered voters | -0.665*** | 0.717*** | -0.047+ |
|  | (0.071) | (0.059) | (0.024) |
| Total number of votes (in 000s) | 0.001 | -0.001 | 0.000 |
|  | (0.002) | (0.002) | (0.001) |
| Constant | 0.588*** | 0.334*** | 0.068*** |
|  | (0.033) | (0.027) | (0.011) |
|  |  |  |  |
| R^2^ | 0.634 | 0.730 | 0.022 |
| N | 186 | 186 | 186 |

Notes: Cell entries are regression coefficients predicting each candidate’s vote share using the predictors listed in the first column with standard errors in parentheses.

***p<.001 **p<.01 *p<.05 +p<.10

Table S51

*Name Order Effects in the 2016 Congressional District 2 Election in New Hampshire: Name Order*

|  | Dependent Variable | | |
| --- | --- | --- | --- |
| Predictor | Vote share of the Democratic candidate (Ann McLane Kuster)  (1) | Vote share of the Republican candidate (Jim Lawrence)  (2) | Vote share of the other candidate (John J. Babiarz, Independent)  (3) |
| Name order | -0.011* | -0.012** | -0.003 |
|  | (0.005) | (0.004) | (0.002) |
| % Democratic registered voters | 0.352*** | -0.332*** | -0.017 |
|  | (0.056) | (0.046) | (0.019) |
| % Republican registered voters | -0.661*** | 0.726*** | -0.051* |
|  | (0.070) | (0.059) | (0.024) |
| Total number of votes (in 000s) | 0.001 | -0.001 | 0.000 |
|  | (0.002) | (0.002) | (0.001) |
| Constant | 0.590*** | 0.337*** | 0.069*** |
|  | (0.033) | (0.027) | (0.011) |
|  |  |  |  |
| R^2^ | 0.638 | 0.731 | 0.035 |
| N | 186 | 186 | 186 |

Notes: Cell entries are regression coefficients predicting each candidate’s vote share using the predictors listed in the first column with standard errors in parentheses.

***p<.001 **p<.01 *p<.05 +p<.10

Table S52

*Name Order Effects in the 2016 Congressional District 2 Election in New Hampshire: Name Order and Name Order Squared*

|  | Dependent Variable | | |
| --- | --- | --- | --- |
| Predictor | Vote share of the Democratic candidate (Ann McLane Kuster)  (1) | Vote share of the Republican candidate (Jim Lawrence)  (2) | Vote share of the other candidate (John J. Babiarz, Independent)  (3) |
| Name order | -0.012* | -0.012** | -0.003 |
|  | (0.005) | (0.004) | (0.002) |
| Name order squared | -0.004 | 0.004 | -0.010** |
|  | (0.009) | (0.007) | (0.003) |
| % Democratic registered voters | 0.353*** | -0.335*** | -0.026 |
|  | (0.056) | (0.047) | (0.019) |
| % Republican registered voters | -0.659*** | 0.723*** | -0.062* |
|  | (0.071) | (0.059) | (0.024) |
| Total number of votes (in 000s) | 0.001 | -0.001 | 0.000 |
|  | (0.002) | (0.002) | (0.001) |
| Constant | 0.591*** | 0.336*** | 0.081*** |
|  | (0.033) | (0.027) | (0.012) |
|  |  |  |  |
| R^2^ | 0.638 | 0.731 | 0.087 |
| N | 186 | 186 | 186 |

Notes: Cell entries are regression coefficients predicting each candidate’s vote share using the predictors listed in the first column with standard errors in parentheses.

***p<.001 **p<.01 *p<.05 +p<.10

**Appendix A: New Hampshire Laws for Ballot Name Order**

**Statute**. Title 63, 656:5a –

1. Whenever there are 2 or more candidates for the same office whose names will appear together within the same column or list on a ballot, the position of such names shall be determined according to this section.
2. Immediately following the close of the period during which a person may accept the nomination of a party committee pursuant to RSA 655:32, the secretary of state or designee shall conduct a public random selection of a whole number from one to the total number of candidates for each possible list length where a group of candidates for the same office may appear in the same list in state or local elections during the next 2 years. For example, for a possible list of 3 candidates, the number one, 2, or 3 shall be randomly selected. The seed number for each possible list length shall remain in effect for the 2 years until the next random selection of seed numbers.
3. To determine the order of names on each ballot, the candidates for each office in the same list shall be temporarily listed alphabetically by surnames and the positions in such list shall be temporarily numbered in ascending order. The candidate whose position in the initial temporary list equals the seed number selected under paragraph II for the appropriate list length shall appear first on the ballot. The order of candidates after the candidate in the first position shall follow alphabetically by surname with "a'' following "z.''

**Statute**. Title 63, 656:5 Party Columns.

1. The names of all candidates nominated in accordance with the election laws shall be arranged upon the state general election ballot in successive party columns. Each separate column shall contain the names of the candidates of one party; except that, if only a part of a full list of candidates is nominated by a political party, 2 or more such lists may be arranged whenever practicable in the same column. The party columns that list the names of candidates for offices that elect more than one person shall stagger the names of the candidates so that they do not line up evenly in a horizontal direction. The left-most column shall begin one line below the column to its right. The secretary of state shall determine the vertical location of any additional columns that may appear on the ballot.
2. The position of party columns shall be rotated on the ballots used so that each party column shall appear thereon, to the extent practicable, an approximately equal number of times in the first, last, and each intermediate column position across the state, without requiring more than one unique column order or ballot format for each town, ward, or unincorporated place. Starting with the general election for 2012 and following each new apportionment of representative districts, but before the close of the period during which a person may accept the nomination of a party committee pursuant to RSA 655:32, the secretary of state shall develop as many generic column rotation plans for use in general elections as he or she might reasonably expect to be needed for different possible numbers of party columns on the general election ballot. If the number of party columns expected on the general election ballot changes such that one or more additional generic column rotation plans are needed, the secretary of state shall, from time to time, prepare such additional plans as are needed for any general election.
3. The generic column rotation plans shall be based on a reasonably balanced rotation of party columns within and across all non-floterial state representative districts, those being the smallest representative districts to which each voting place is apportioned pursuant to part I, article 11 of the New Hampshire constitution. Consideration shall also be given to reasonably minimize any obvious, substantial, and avoidable imbalances in column rotation within senate districts. The average deviation from equal rotation for the first party column position, measured across the state as a whole and based on population according to the last decennial federal census, shall be as close to 0 percent as is practicable but in no event greater than 1 percent. Once generic column rotation plans are established the secretary of state shall publish such plans to the department's website.
4. Immediately following the close of the period during which a person may accept the nomination of a party committee pursuant to RSA 655:32, the secretary of state or designee shall publicly select by lot the actual party columns to be positioned according to the generic column rotation plan established pursuant to paragraphs II and III. No party shall be assigned the same generic party column designation for 2 consecutive general elections.

**Chapter 669 TOWN ELECTIONS 669.23**. Whenever there are 2 or more candidates for the same office the names shall be printed upon the ballot in the alphabetical order of their surnames according to the alphabetization procedure established in RSA 656:5-a.

**Partisan races.** Republican and Democratic Party candidates are listed first, in descending order of the total number of votes cast state-wide for the candidates of their party in all races in the most recent State general election. All other candidates are listed next in the order in which they were qualified by the State to run for office. If two or more candidates affiliated with the same party are running for the same office, the candidate names are listed alphabetically.

**Nonpartisan races.** New Hampshire does not hold nonpartisan statewide elections. For local nonpartisan elections, however, candidates are listed alphabetically by last name.

**Appendix B: Equivalence Checks Tables**

Table SB1

*T-Tests Predicting Total Votes in the Race with Candidate Name Order for Two-Candidate Races in 2000, 2002, and 2004 Primaries in New Hampshire*

| Race | T-value | p-value | N |
| --- | --- | --- | --- |
|  |  |  |  |
| 1st Cong. District Democratic Primary 2002 | -.36 | .72 | 114 |
|  |  |  |  |
| 1st Cong. District Republican Primary 2004 | -.18 | .86 | 114 |
|  |  |  |  |
| 2nd Cong. District Democratic Primary 2000 | .20 | .86 | 186 |
|  |  |  |  |
| 2nd Cong. District Democratic Primary 2002 | -.70 | .49 | 186 |
|  |  |  |  |
| 2nd Cong. District Democratic Primary 2004 | .87 | .39 | 186 |
|  |  |  |  |
| 2nd Cong. District Republican Primary 2002 | -.35 | .73 | 188 |
|  |  |  |  |
| 2nd Cong. District Republican Primary 2004 | -.44 | .66 | 188 |
|  |  |  |  |
| Governor Democratic Primary 2000 | -1.26 | .21 | 301 |
|  |  |  |  |
| Governor Democratic Primary 2002 | .40 | .69 | 302 |
|  |  |  |  |
| Governor Democratic Primary 2004 | .26 | .79 | 300 |
|  |  |  |  |
| Governor Republican Primary 2004 | -.42 | .81 | 302 |

Notes: Cell entries are t-statistic and p-value of t-tests comparing the number of total votes who saw one specific candidate in first and second position in columns (1) and (2), respectively. Each row is a separate t-test.

Table SB2

*ANOVAs Predicting Total Votes in the Race with Candidate Name Order for 3+Candidate Races in 2000, 2002, and 2004 Primaries in New Hampshire*

| Race | F-statistic | p-value | N |
| --- | --- | --- | --- |
|  |  |  |  |
| Senate Republican Primary 2002 | .04 | .96 | 302 |
|  |  |  |  |
| Senate Republican Primary 2004 | .28 | .75 | 302 |
|  |  |  |  |
| 1st Cong. District Democratic Primary 2004 | .08 | .97 | 114 |
|  |  |  |  |
| 1st Cong. District Republican Primary 2002 | .28 | .96 | 114 |
|  |  |  |  |
| Governor Republican Primary 2000 | .07 | .99 | 300 |
|  |  |  |  |
| Governor Republican Primary 2002 | .08 | 1.00 | 302 |

Notes: Cell entries are F-statistic and p-value ANOVA predicting total votes using name order positions in columns (1) and (2), respectively. Each row is a separate ANOVA test.

Table SB3

*T-Tests Predicting Registered Votes in the Race with Candidate Name Order for Two-Candidate Races in 2000, 2002, and 2004 Primaries in New Hampshire*

| Race | T-value | p-value | N |
| --- | --- | --- | --- |
|  |  |  |  |
| 1st Cong. District Democratic Primary 2002 | -.29 | .77 | 114 |
|  |  |  |  |
| 1st Cong. District Republican Primary 2004 | -.56 | .58 | 114 |
|  |  |  |  |
| 2nd Cong. District Democratic Primary 2000 | -.77 | .44 | 186 |
|  |  |  |  |
| 2nd Cong. District Democratic Primary 2002 | -.26 | .79 | 186 |
|  |  |  |  |
| 2nd Cong. District Democratic Primary 2004 | .58 | .57 | 186 |
|  |  |  |  |
| 2nd Cong. District Republican Primary 2002 | -.50 | .62 | 188 |
|  |  |  |  |
| 2nd Cong. District Republican Primary 2004 | -.15 | .88 | 188 |
|  |  |  |  |
| Governor Democratic Primary 2000 | -.82 | .42 | 301 |
|  |  |  |  |
| Governor Democratic Primary 2002 | -.44 | .66 | 302 |
|  |  |  |  |
| Governor Democratic Primary 2004 | .42 | .68 | 300 |
|  |  |  |  |
| Governor Republican Primary 2004 | -.28 | .78 | 302 |

Notes: Cell entries are t-statistic and p-value of t-tests comparing the number of total votes who saw one specific candidate in first and second position in columns (1) and (2), respectively. Each row is a separate t-test.

Table SB4

*ANOVAs Predicting Registered Votes in the Race with Candidate Name Order for 3+Candidate Races in 2000, 2002, and 2004 Primaries in New Hampshire*

| Race | F-statistic | p-value | N |
| --- | --- | --- | --- |
|  |  |  |  |
| Senate Republican Primary 2002 | .01 | .99 | 302 |
|  |  |  |  |
| Senate Republican Primary 2004 | .16 | .85 | 302 |
|  |  |  |  |
| 1st Cong. District Democratic Primary 2004 | .13 | .94 | 114 |
|  |  |  |  |
| 1st Cong. District Republican Primary 2002 | .38 | .91 | 114 |
|  |  |  |  |
| Governor Republican Primary 2000 | .13 | .97 | 300 |
|  |  |  |  |
| Governor Republican Primary 2002 | .02 | 1.00 | 302 |

Notes: Cell entries are F-statistic and p-value ANOVA predicting registered votes with candidate name order in columns (1) and (2), respectively. Each row is a separate ANOVA test.

Table SB5

*T-Tests Predicting Registered Democrats or Republicans (Depending on Primary) in the Race with Candidate Name Order for Two-Candidate Races in 2000, 2002, and 2004 Primaries in New Hampshire*

| Race | T-value | p-value | N |
| --- | --- | --- | --- |
|  |  |  |  |
| 1st Cong. District Democratic Primary 2002 | -.42 | .67 | 114 |
|  |  |  |  |
| 1st Cong. District Republican Primary 2004 | -.61 | .54 | 114 |
|  |  |  |  |
| 2nd Cong. District Democratic Primary 2000 | -.41 | .69 | 186 |
|  |  |  |  |
| 2nd Cong. District Democratic Primary 2002 | -.61 | .54 | 186 |
|  |  |  |  |
| 2nd Cong. District Democratic Primary 2004 | .09 | .93 | 186 |
|  |  |  |  |
| 2nd Cong. District Republican Primary 2002 | -.36 | .72 | 188 |
|  |  |  |  |
| 2nd Cong. District Republican Primary 2004 | -.14 | .89 | 188 |
|  |  |  |  |
| Governor Democratic Primary 2000 | -.91 | .37 | 301 |
|  |  |  |  |
| Governor Democratic Primary 2002 | -.15 | .88 | 302 |
|  |  |  |  |
| Governor Democratic Primary 2004 | -.06 | .96 | 300 |
|  |  |  |  |
| Governor Republican Primary 2004 | -.31 | .76 | 302 |

Notes: Cell entries are t-statistic and p-value of t-tests comparing registered Democrats or Republicans (depending on primary) who saw one specific candidate in first and second position in columns (1) and (2), respectively. Each row is a separate t-test.

Table SB6

*ANOVAs Predicting Registered Democrats or Republicans (Depending on Primary) in the Race with Candidate Name Order for 3+Candidate Races in 2000, 2002, and 2004 Primaries in New Hampshire*

| Race | F-statistic | p-value | N |
| --- | --- | --- | --- |
|  |  |  |  |
| Senate Republican Primary 2002 | .03 | .98 | 302 |
|  |  |  |  |
| Senate Republican Primary 2004 | .07 | .93 | 302 |
|  |  |  |  |
| 1st Cong. District Democratic Primary 2004 | .09 | .97 | 114 |
|  |  |  |  |
| 1st Cong. District Republican Primary 2002 | .39 | .91 | 114 |
|  |  |  |  |
| Governor Republican Primary 2000 | .13 | .97 | 300 |
|  |  |  |  |
| Governor Republican Primary 2002 | .04 | 1.00 | 302 |

Notes: Cell entries are F-statistic and p-value ANOVA predicting registered Democrats or Republicans (depending on primary) with candidate name positions in columns (1) and (2), respectively. Each row is a separate ANOVA test.

Table SB7

*T-Tests Predicting Undeclared Registered Voters in the Race with Candidate Name Order for Two-Candidate Races in 2000, 2002, and 2004 Primaries in New Hampshire*

| Race | T-value | p-value | N |
| --- | --- | --- | --- |
|  |  |  |  |
| 1st Cong. District Democratic Primary 2002 | -.77 | .45 | 114 |
|  |  |  |  |
| 1st Cong. District Republican Primary 2004 | -.92 | .36 | 114 |
|  |  |  |  |
| 2nd Cong. District Democratic Primary 2000 | -1.22 | .23 | 186 |
|  |  |  |  |
| 2nd Cong. District Democratic Primary 2002 | -.14 | .89 | 186 |
|  |  |  |  |
| 2nd Cong. District Democratic Primary 2004 | .77 | .44 | 186 |
|  |  |  |  |
| 2nd Cong. District Republican Primary 2002 | -.49 | .63 | 188 |
|  |  |  |  |
| 2nd Cong. District Republican Primary 2004 | .12 | .91 | 188 |
|  |  |  |  |
| Governor Democratic Primary 2000 | -.99 | .32 | 301 |
|  |  |  |  |
| Governor Democratic Primary 2002 | -.52 | .60 | 302 |
|  |  |  |  |
| Governor Democratic Primary 2004 | .51 | .61 | 300 |
|  |  |  |  |
| Governor Republican Primary 2004 | -.37 | .71 | 302 |

Notes: Cell entries are t-statistic and p-value of t-tests comparing undeclared registered voters who saw one specific candidate in first and second position in columns (1) and (2), respectively. Each row is a separate t-test.

Table SB8

*ANOVAs Predicting Undeclared Registered Voters in the Race with Candidate Name Order for 3+Candidate Races in 2000, 2002, and 2004 Primaries in New Hampshire*

| Race | F-statistic | p-value | N |
| --- | --- | --- | --- |
|  |  |  |  |
| Senate Republican Primary 2002 | .11 | .90 | 302 |
|  |  |  |  |
| Senate Republican Primary 2004 | .41 | .67 | 302 |
|  |  |  |  |
| 1st Cong. District Democratic Primary 2004 | .07 | .98 | 114 |
|  |  |  |  |
| 1st Cong. District Republican Primary 2002 | .39 | .91 | 114 |
|  |  |  |  |
| Governor Republican Primary 2000 | .22 | .93 | 300 |
|  |  |  |  |
| Governor Republican Primary 2002 | .05 | 1.00 | 302 |

Notes: Cell entries are F-statistic and p-value ANOVA predicting undeclared registered voters with candidate name positions in columns (1) and (2), respectively. Each row is a separate ANOVA test.

Table SB9

*T-Tests Predicting Total Number of People who Registered on Election Day in the Race with Candidate Name Order for Two-Candidate Races in 2000, 2002, and 2004 Primaries in New Hampshire*

| Race | T-value | p-value | N |
| --- | --- | --- | --- |
|  |  |  |  |
| 1st Cong. District Democratic Primary 2002 | na | na | na |
|  |  |  |  |
| 1st Cong. District Republican Primary 2004 | .45 | .65 | 114 |
|  |  |  |  |
| 2nd Cong. District Democratic Primary 2000 | -.29 | .77 | 184 |
|  |  |  |  |
| 2nd Cong. District Democratic Primary 2002 | na | na | na |
|  |  |  |  |
| 2nd Cong. District Democratic Primary 2004 | -.27 | .78 | 186 |
|  |  |  |  |
| 2nd Cong. District Republican Primary 2002 | na | na | na |
|  |  |  |  |
| 2nd Cong. District Republican Primary 2004 | .24 | .81 | 188 |
|  |  |  |  |
| Governor Democratic Primary 2000 | -1.06 | .29 | 299 |
|  |  |  |  |
| Governor Democratic Primary 2002 | na | na | 302 |
|  |  |  |  |
| Governor Democratic Primary 2004 | -.24 | .81 | 300 |
|  |  |  |  |
| Governor Republican Primary 2004 | .66 | .51 | 302 |

Notes: Cell entries are t-statistic and p-value of t-tests comparing total number of people who registered on Election Day who saw one specific candidate in first and second position in columns (1) and (2), respectively. Each row is a separate t-test. Cells with “na” are the races for which data were not available.

Table SB10

*ANOVAs Predicting Total Number of People who Registered on Election Day with Candidate Name Order for 3+Candidate Races in 2000, 2002, and 2004 Primaries in New Hampshire*

| Race | F-statistic | p-value | N |
| --- | --- | --- | --- |
|  |  |  |  |
| Senate Republican Primary 2002 | na | na | na |
|  |  |  |  |
| Senate Republican Primary 2004 | .10 | .91 | 302 |
|  |  |  |  |
| 1st Cong. District Democratic Primary 2004 | .04 | .99 | 114 |
|  |  |  |  |
| 1st Cong. District Republican Primary 2002 | na | na | na |
|  |  |  |  |
| Governor Republican Primary 2000 | .22 | .93 | 298 |
|  |  |  |  |
| Governor Republican Primary 2002 | na | na | na |

Notes: Cell entries are F-statistic and p-value ANOVA predicting total number of people who registered on Election Day with candidate name positions in columns (1) and (2), respectively. Each row is a separate ANOVA test. Cells with “na” are the races for which data were not available.

Table SB11

*ANOVAs Predicting Total Votes with Candidate Name Order in 2012 and 2016 General Election in New Hampshire*

| Race | F-statistic | p-value | N |
| --- | --- | --- | --- |
| Presidential 2012 (Democratic candidate) | .56 | .57 | 300 |
| Presidential 2012 (Republican candidate) | .89 | .41 | 300 |
| Presidential 2012 (Other Party candidate) | .05 | .95 | 300 |
|  |  |  |  |
| Gubernatorial 2012 (Democratic candidate) | .58 | .56 | 300 |
| Gubernatorial 2012 (Republican candidate) | .89 | .41 | 300 |
| Gubernatorial 2012 (Other Party candidate) | .05 | .96 | 300 |
|  |  |  |  |
| Congressional District 1 2012 (Democratic candidate) | 1.09 | .15 | 114 |
| Congressional District 1 2012 (Republican candidate) | 1.29 | .28 | 114 |
| Congressional District 1 2012 (Other Party candidate) | .08 | .92 | 114 |
|  |  |  |  |
| Congressional District 2 2012 (Democratic candidate) | .21 | .81 | 186 |
| Congressional District 2 2012 (Republican candidate) | .08 | .92 | 186 |
| Congressional District 2 2012 (Other Party candidate) | .37 | .69 | 186 |
|  |  |  |  |
| Presidential 2016 (Democratic candidate) | .70 | .50 | 300 |
| Presidential 2016 (Republican candidate) | .03 | .97 | 300 |
| Presidential 2016 (Other Party candidate) | .39 | .66 | 300 |
|  |  |  |  |
| Senate 2016 (Democratic candidate) | .85 | .43 | 300 |
| Senate 2016 (Republican candidate) | .06 | .94 | 300 |
| Senate 2016 (Other Party candidate) | .46 | .63 | 300 |
|  |  |  |  |
| Gubernatorial 2016 (Democratic candidate) | .86 | .42 | 300 |
| Gubernatorial 2016 (Republican candidate) | .07 | .93 | 300 |
| Gubernatorial 2016 (Other Party candidate) | .44 | .65 | 300 |
|  |  |  |  |
| Congressional District 1 2016 (Democratic candidate) | 1.13 | .33 | 114 |
| Congressional District 1 2016 (Republican candidate) | .06 | .95 | 114 |
| Congressional District 1 2016 (Other Party candidate) | 1.63 | .20 | 114 |
|  |  |  |  |
| Congressional District 2 2016 (Democratic candidate) | .08 | .92 | 186 |
| Congressional District 2 2016 (Republican candidate) | .39 | .68 | 186 |
| Congressional District 2 2016 (Other Party candidate) | .18 | .84 | 186 |

Notes: Cell entries are F-statistic and p-value ANOVA predicting total votes for the candidate of each party in each race with candidate name positions in columns (1) and (2), respectively. Each row is a separate ANOVA test.

Table SB12

*ANOVAs Predicting Registered Voters with Candidate Name Order in 2012 and 2016 General Election in New Hampshire*

| Race | F-statistic | p-value | N |
| --- | --- | --- | --- |
| 2012 (Democratic candidate) | .44 | .64 | 300 |
| 2012 (Republican candidate) | .85 | .43 | 300 |
| 2012 (Other Party candidate) | .08 | .93 | 300 |
|  |  |  |  |
| 2016 (Democratic candidate) | .82 | .44 | 300 |
| 2016 (Republican candidate) | .08 | .92 | 300 |
| 2016 (Other Party candidate) | .37 | .69 | 300 |

Notes: Cell entries are F-statistic and p-value ANOVA predicting registered voters for the candidate of each party with candidate name positions in columns (1) and (2), respectively. Each row is a separate ANOVA test.

Table SB13

*ANOVAs Predicting Registered Democratic Voters with Candidate Name Order in 2012 and 2016 General Election in New Hampshire*

| Race | F-statistic | p-value | N |
| --- | --- | --- | --- |
| 2012 (Democratic candidate) | .44 | .64 | 300 |
| 2012 (Republican candidate) | .50 | .60 | 300 |
| 2012 (Other Party candidate) | .02 | .98 | 300 |
|  |  |  |  |
| 2016 (Democratic candidate) | .54 | .58 | 300 |
| 2016 (Republican candidate) | .03 | .97 | 300 |
| 2016 (Other Party candidate) | .40 | .67 | 300 |

Notes: Cell entries are F-statistic and p-value ANOVA predicting registered Democratic voters for the candidate of each party with candidate name positions in columns (1) and (2), respectively. Each row is a separate ANOVA test.

Table SB14

*ANOVAs Predicting Registered Republican Voters with Candidate Name Order in 2012 and 2016 General Election in New Hampshire*

| Race | F-statistic | p-value | N |
| --- | --- | --- | --- |
| 2012 (Democratic candidate) | 1.00 | .37 | 300 |
| 2012 (Republican candidate) | 1.35 | .26 | 300 |
| 2012 (Other Party candidate) | .01 | .99 | 300 |
|  |  |  |  |
| 2016 (Democratic candidate) | 1.29 | .28 | 300 |
| 2016 (Republican candidate) | .04 | .96 | 300 |
| 2016 (Other Party candidate) | .84 | .43 | 300 |

Notes: Cell entries are F-statistic and p-value ANOVA predicting registered Republican voters for the candidate of each party with candidate name positions in columns (1) and (2), respectively. Each row is a separate ANOVA test.

Table SB15

*ANOVAs Predicting Registered Undeclared Voters with Candidate Name Order in 2012 and 2016 General Election in New Hampshire*

| Race | F-statistic | p-value | N |
| --- | --- | --- | --- |
| 2012 (Democratic candidate) | .17 | .84 | 300 |
| 2012 (Republican candidate) | .65 | .52 | 300 |
| 2012 (Other Party candidate) | .25 | .78 | 300 |
|  |  |  |  |
| 2016 (Democratic candidate) | .60 | .55 | 300 |
| 2016 (Republican candidate) | .21 | .81 | 300 |
| 2016 (Other Party candidate) | .14 | .87 | 300 |

Notes: Cell entries are F-statistic and p-value ANOVA predicting registered undeclared voters for the candidate of each party with candidate name positions in columns (1) and (2), respectively. Each row is a separate ANOVA test.

Table SB16

*ANOVAs Predicting Major Party Presidential Candidates’ Vote Shares in 2008 (and 2012) General Election with Candidate Name Order in 2012 (and 2016) General Election in New Hampshire*

| Race | F-statistic | p-value | N |
| --- | --- | --- | --- |
|  | | | |
| *Predicting Democratic Candidate’s Vote Share in 2008 Presidential Election* | | | |
| 2012 (Democratic candidate) | 1.77 | .17 | 300 |
| 2012 (Republican candidate) | .68 | .51 | 300 |
| 2012 (Other Party candidate) | .88 | .42 | 300 |
|  |  |  |  |
| *Predicting Republican Candidate’s Vote Share in 2008 Presidential Election* | | | |
| 2012 (Democratic candidate) | 1.88 | .16 | 300 |
| 2012 (Republican candidate) | .83 | .44 | 300 |
| 2012 (Other Party candidate) | .76 | .41 | 300 |
|  |  |  |  |
| *Predicting Democratic Candidate’s Vote Share in 2012 Presidential Election* | | | |
| 2012 (Democratic candidate) | .84 | .43 | 300 |
| 2012 (Republican candidate) | .01 | .99 | 300 |
| 2012 (Other Party candidate) | .69 | .78 | 300 |
|  |  |  |  |
| *Predicting Republican Candidate’s Vote Share in 2012 Presidential Election* | | | |
| 2016 (Democratic candidate) | 1.32 | .27 | 300 |
| 2016 (Republican candidate) | .07 | .93 | 300 |
| 2016 (Other Party candidate) | .73 | .48 | 300 |

Notes: Cell entries are F-statistic and p-value ANOVA predicting major party presidential candidates’ vote shares in 2008 (and 2012) general election for the candidate of each party with candidate name positions in columns (1) and (2), respectively. Each row is a separate ANOVA test.

**Appendix C: Robustness Checks Tables**

Table SC1

*Name Order Effects in Three-Candidate 2002 Republican Primary for US Senate in New Hampshire: Second/Third Versus First*

|  | Dependent Variable | | |
| --- | --- | --- | --- |
| Predictor | Vote share of Smith | Vote share of Stremsky | Vote share of Sununu |
| Listed second (vs. first) | -.01 | .002 | .01 |
|  | (.01) | (.002) | (.01) |
| Listed third (vs. first) | -.02* | -.001 | -.01 |
|  | (.01) | (.002) | (.01) |
| Total number of votes (in 000s) | -.04*** | -.002 | .04*** |
|  | (.01) | (.001) | (.01) |
| Constant | .50*** | .02*** | .49*** |
|  | (.01) | (.001) | (.01) |
|  |  |  |  |
| R^2^ | .09 | .02 | .09 |
| N | 302 | 302 | 302 |

Notes: Cell entries are regression coefficients predicting each candidate’s vote share using the predictors listed in the first column with standard errors in parentheses.

***p<.001 **p<.01 *p<.05 +p<.10

Table SC2

*Name Order Effects in Three-Candidate 2004 Republican Primary for US Senate in New Hampshire: Second/Third Versus First*

|  | Dependent Variable | | |
| --- | --- | --- | --- |
| Predictor | Vote share of Aciere | Vote share of Gregg | Vote share of Tipa |
| Listed second (vs. first) | -.01* | -.02+ | -.004 |
|  | (.004) | (.01) | (.004) |
| Listed third (vs. first) | -.02*** | -.02 | -.02*** |
|  | (.004) | (.01) | (.004) |
| Total number of votes (in 000s) | .002 | .02 | -.002 |
|  | (.01) | (.02) | (.01) |
| Constant | .05*** | .92*** | .05*** |
|  | (.003) | (.01) | (.004) |
|  |  |  |  |
| R^2^ | .08 | .02 | .04 |
| N | 302 | 302 | 302 |

Notes: Cell entries are regression coefficients predicting each candidate’s vote share using the predictors listed in the first column with standard errors in parentheses.

***p<.001 **p<.01 *p<.05 +p<.10

Table SC3

*Name Order Effects in Five-Candidate 2000 Republican Primary for Governor in New Hampshire: Second/Third/Fourth/Fifth Versus First*

|  | Dependent Variable | | | | |
| --- | --- | --- | --- | --- | --- |
| Predictor | Vote share of Bramante | Vote share of Howard | Vote share of Humphrey | Vote share of Marron | Vote share of Squires |
| Listed second (vs. first) | -.01 | -.02 | -.02 | -.002 | .002 |
|  | (.003) | (.02) | (.02) | (.002) | (.02) |
| Listed third (vs. first) | -.01 | .01 | -.01 | -.004* | -.002 |
|  | (.003) | (.02) | (.02) | (.002) | (.02) |
| Listed fourth (vs. first) | -.01* | -.01 | -.04+ | -.003+ | -.004 |
|  | (.003) | (.02) | (.02) | (.002) | (.02) |
| Listed fifth (vs. first) | -.01* | -.02 | -.01 | -.001 | .00 |
|  | (.003) | (.02) | (.02) | (.002) | (.02) |
| Total number of votes (in 000s) | .01* | -.01 | -.05* | -.003+ | .06*** |
|  | (.003) | (.02) | (.02) | (.002) | (.02) |
| Constant | .02*** | .22*** | .56*** | .01*** | .19*** |
|  | (.002) | (.01) | (.01) | (.001) | (.02) |
|  |  |  |  |  |  |
| R^2^ | .03 | .02 | .03 | .03 | .04 |
| N | 300 | 300 | 300 | 300 | 300 |

Notes: Cell entries are regression coefficients predicting each candidate’s vote share using the predictors listed in the first column with standard errors in parentheses.

***p<.001 **p<.01 *p<.05 +p<.10

Table SC4

*Name Order Effects in Six-Candidate 2002 Republican Primary for Governor in New Hampshire: Second/Third/Fourth/Fifth/Sixth Versus First*

|  | Dependent Variable | | | | | |
| --- | --- | --- | --- | --- | --- | --- |
| Predictor | Vote share of Benson | Vote share of Haas | Vote share of Humphrey | Vote share of Keough | Vote share of Kingsbury | Vote share of Kroepel |
| Listed second (vs. first) | .01 | -.003+ | -.02 | -.03 | .00 | -.001 |
|  | (.02) | (.002) | (.02) | (.02) | (.001) | (.001) |
| Listed third (vs. first) | -.002 | -.004* | -.01 | .003 | -.001 | -.002+ |
|  | (.02) | (.002) | (.02) | (.02) | (.001) | (.001) |
| Listed fourth (vs. first) | .01 | -.004* | -.01 | -.01 | .00 | -.003* |
|  | (.02) | (.002) | (.02) | (.02) | (.001) | (.001) |
| Listed fifth (vs. first) | .01 | -.003+ | -.01 | -.01 | .00 | .00 |
|  | (.02) | (.002) | (.02) | (.02) | (.001) | (.001) |
| Listed sixth (vs. first) | -.01 | -.001 | -.02 | -.01 | .00 | .001 |
|  | (.02) | (.002) | (.02) | (.02) | (.001) | (.001) |
| Total number of votes (in 000s) | .01 | .00 | .00 | -.01 | -.001* | -.001 |
|  | (.01) | (.001) | (.01) | (.01) | (.001) | (.001) |
| Constant | .35*** | .01*** | .29*** | .35*** | .01*** | .01*** |
|  | (.01) | (.01) | (.01) | (.02) | (.001) | (.001) |
|  |  |  |  |  |  |  |
| R^2^ | .01 | .03 | .01 | .01 | .02 | .05 |
| N | 302 | 302 | 302 | 302 | 302 | 302 |

Notes: Cell entries are regression coefficients predicting each candidate’s vote share using the predictors listed in the first column with standard errors in parentheses.

***p<.001 **p<.01 *p<.05 +p<.10

Table SC5

*Name Order Effects in Eight-Candidate 2002 Republican Primary for 2nd Congressional District in New Hampshire: Second/Third/Fourth/Fifth/Sixth/Seven/Eighth Versus First*

|  | Dependent Variable | | | | | | | |
| --- | --- | --- | --- | --- | --- | --- | --- | --- |
| Predictor | Vote share of Barrow | Vote share of Bevill | Vote share of Bradley | Vote share of Clark | Vote share of Hoffman | Vote share of Mahoney | Vote share of Stephen | Vote share of Wendelboe |
| Listed second (vs. first) | -.004 | .001 | .03 | -.04+ | -.01 | -.01 | -.04 | -.02 |
|  | (.02) | (.002) | (.05) | (.02) | (.004) | (.03) | (.05) | (.02) |
| Listed third (vs. first) | -.03 | -.001 | -.04 | -.05* | -.01* | -.04 | -.05 | -.004 |
|  | (.02) | (.003) | (.05) | (.02) | (.003) | (.03) | (.05) | (.02) |
| Listed fourth (vs. first) | .02 | .002 | -.02 | -.03 | -.01* | -.07* | -.07 | -.01 |
|  | (.02) | (.002) | (.05) | (.02) | (.004) | (.03) | (.05) | (.02) |
| Listed fifth (vs. first) | .01 | .00 | -.02 | -.01 | -.01*** | -.07* | -.03 | -.02 |
|  | (.01) | (.003) | (.05) | (.02) | (.004) | (.03) | (.04) | (.02) |
| Listed sixth (vs. first) | .003 | -.003 | -.06 | -.04* | -.01* | -.04 | -.02 | .002 |
|  | (.02) | (.003) | (.05) | (.02) | (.004) | (.03) | (.05) | (.02) |
| Listed seventh (vs. first) | -.02 | .00 | -.06 | -.05* | -.01** | -.05 | -.02 | -.01 |
|  | (.02) | (.003) | (.05) | (.02) | (.004) | (.03) | (.05) | (.02) |
| Listed eighth (vs. first) | -.02 | .00 | -.03 | -.03 | -.01** | -.04 | -.06 | -.01 |
|  | (.02) | (.002) | (.03) | (.02) | (.004) | (.03) | (.05) | (.02) |
| Total number of votes (in 000s) | -.01 | .00 | -.04+ | -.002 | .00 | -.02* | .08*** | -.003 |
|  | (.01) | (.001) | (.02) | (.01) | (.002) | (.01) | (.02) | (.01) |
| Constant | .10*** | .01*** | .38*** | .13*** | .02*** | .26*** | .18*** | .08*** |
|  | (.02) | (.002) | (.03) | (.02) | (.003) | (.02) | (.03) | (.02) |
|  |  |  |  |  |  |  |  |  |
| R^2^ | .05 | .05 | .03 | .04 | .14 | .12 | .14 | .02 |
| N | 114 | 114 | 114 | 114 | 114 | 114 | 114 | 114 |

Notes: Cell entries are regression coefficients predicting each candidate’s vote share using the predictors listed in the first column with standard errors in parentheses.

***p<.001 **p<.01 *p<.05 +p<.10

Table SC6

*Name Order Effects in 4-Candidate 2004 Democratic Primary for 1st Congressional District in New Hampshire: Second/Third/Fourth Versus First*

|  | Dependent Variable | | | |
| --- | --- | --- | --- | --- |
| Predictor | Vote share of Bruce | Vote share of Duffey | Vote share of Liles | Vote share of Nadeau |
| Listed second (vs. first) | -.05+ | -.03 | -.01 | -.01 |
|  | (.03) | (.03) | (.01) | (.05) |
| Listed third (vs. first) | -.06* | -.08** | -.02*** | -.11* |
|  | (.03) | (.03) | (.01) | (.05) |
| Listed fourth (vs. first) | -.03 | -.07* | -.02*** | -.09* |
|  | (.03) | (.03) | (.01) | (.05) |
| Total number of votes (in 000s) | -.09 | .12+ | -.04** | .03 |
|  | (.06) | (.06) | (.01) | (.09) |
| Constant | .24*** | .26*** | .06*** | .57*** |
|  | (.02) | (.03) | (.01) | (.04) |
|  |  |  |  |  |
| R^2^ | .07 | .11 | .17 | .08 |
| N | 114 | 114 | 114 | 114 |

Notes: Cell entries are regression coefficients predicting each candidate’s vote share using the predictors listed in the first column with standard errors in parentheses.

***p<.001 **p<.01 *p<.05 +p<.10

Table SC7

*Name Order Effects in the 2012 Presidential Election in New Hampshire: Second/Third Versus First*

|  | Dependent Variable | | | |
| --- | --- | --- | --- | --- |
| Predictor | Vote share of the Democratic candidate (Barack Obama)  (1) | Vote share of the Republican candidate (Mitt Romney)  (2) | Vote share of the other candidate (Gary Johnson, Libertarian)  (3) | Vote share of the other candidate (Virgil Goode, Constitution)  (4) |
| Listed second (vs. first) | 0.002 | 0.005 | 0.001 | -0.000 |
|  | (0.007) | (0.007) | (0.001) | (0.000) |
| Listed third (vs. first) | -0.003 | -0.001 | -0.001 | 0.000 |
|  | (0.007) | (0.007) | (0.001) | (0.000) |
| % Democratic registered voters | 0.609*** | -0.576*** | -0.023*** | -0.010*** |
|  | (0.048) | (0.047) | (0.006) | (0.003) |
| % Republican registered voters | -0.575*** | 0.610*** | -0.030*** | -0.010*** |
|  | (0.051) | (0.049) | (0.007) | (0.003) |
| Total number of votes (in 000s) | -0.007*** | 0.007*** | -0.000 | -0.000 |
|  | (0.001) | (0.001) | (0.000) | (0.000) |
| Constant | 0.562*** | 0.404*** | 0.027*** | 0.007*** |
|  | (0.025) | (0.024) | (0.003) | (0.001) |
|  |  |  |  |  |
| R^2^ | 0.725 | 0.736 | 0.087 | 0.076 |
| N | 300 | 300 | 300 | 300 |

Notes: Cell entries are regression coefficients predicting each candidate’s vote share using the predictors listed in the first column with standard errors in parentheses.

***p<.001 **p<.01 *p<.05 +p<.10

Table SC8

*Name Order Effects in the 2012 Gubernatorial Election in New Hampshire: Second/Third Versus First*

|  | Dependent Variable | | |
| --- | --- | --- | --- |
| Predictor | Vote share of the Democratic candidate (Maggie Hassan)  (1) | Vote share of the Republican candidate (Ovide Lamontagne)  (2) | Vote share of the other candidate (John J. Babiarz, Libertarian)  (3) |
| Listed second (vs. first) | -0.000 | -0.004 | 0.003+ |
|  | (0.007) | (0.007) | (0.002) |
| Listed third (vs. first) | -0.004 | -0.007 | -0.003 |
|  | (0.007) | (0.007) | (0.002) |
| % Democratic registered voters | 0.701*** | -0.675*** | -0.026* |
|  | (0.046) | (0.047) | (0.013) |
| % Republican registered voters | -0.480*** | 0.528*** | -0.046*** |
|  | (0.049) | (0.049) | (0.014) |
| Total number of votes (in 000s) | -0.005*** | 0.005*** | -0.000 |
|  | (0.001) | (0.001) | (0.000) |
| Constant | 0.524*** | 0.431*** | 0.049*** |
|  | (0.024) | (0.024) | (0.007) |
|  |  |  |  |
| R^2^ | 0.738 | 0.740 | 0.086 |
| N | 300 | 300 | 300 |

Notes: Cell entries are regression coefficients predicting each candidate’s vote share using the predictors listed in the first column with standard errors in parentheses.

***p<.001 **p<.01 *p<.05 +p<.10

Table SC9

*Name Order Effects in the 2012 Congressional District 1Eelection in New Hampshire: Second/Third Versus First*

|  | Dependent Variable | | |
| --- | --- | --- | --- |
| Predictor | Vote share of the Democratic candidate (Carol Shea-Porter)  (1) | Vote share of the Republican candidate (Frank C. Guinta)  (2) | Vote share of the other candidate (Brendan Kelly, Libertarian)  (3) |
| Listed second (vs. first) | 0.010 | 0.013 | 0.009** |
|  | (0.010) | (0.008) | (0.003) |
| Listed third (vs. first) | -0.004 | 0.002 | -0.005 |
|  | (0.009) | (0.009) | (0.003) |
| % Democratic registered voters | 0.453*** | -0.409*** | -0.039 |
|  | (0.075) | (0.068) | (0.025) |
| % Republican registered voters | -0.650*** | 0.697*** | -0.048+ |
|  | (0.075) | (0.068) | (0.025) |
| Total number of votes (in 000s) | -0.003+ | 0.002 | 0.000 |
|  | (0.001) | (0.001) | (0.000) |
| Constant | 0.592*** | 0.334*** | 0.066*** |
|  | (0.040) | (0.036) | (0.013) |
|  |  |  |  |
| R^2^ | 0.784 | 0.819 | 0.180 |
| N | 114 | 114 | 114 |

Notes: Cell entries are regression coefficients predicting each candidate’s vote share using the predictors listed in the first column with standard errors in parentheses.

***p<.001 **p<.01 *p<.05 +p<.10

Table SC10

*Name Order Effects in the 2012 Congressional District 2 Election in New Hampshire: Second/Third Versus First*

|  | Dependent Variable | | |
| --- | --- | --- | --- |
| Predictor | Vote share of the Democratic candidate (Ann McLane Kuster)  (1) | Vote share of the Republican candidate (Charles Bass)  (2) | Vote share of the other candidate (Hardy Macia, Libertarian)  (3) |
| Listed second (vs. first) | -0.011 | -0.010 | 0.008** |
|  | (0.007) | (0.007) | (0.003) |
| Listed third (vs. first) | -0.011 | -0.005 | -0.010** |
|  | (0.007) | (0.007) | (0.003) |
| % Democratic registered voters | 0.803*** | -0.721*** | -0.081*** |
|  | (0.049) | (0.047) | (0.019) |
| % Republican registered voters | -0.517*** | 0.568*** | -0.043* |
|  | (0.053) | (0.050) | (0.021) |
| Total number of votes (in 000s) | -0.006*** | 0.006*** | 0.000 |
|  | (0.002) | (0.002) | (0.001) |
| Constant | 0.465*** | 0.468*** | 0.077*** |
|  | (0.025) | (0.023) | (0.010) |
|  |  |  |  |
| R^2^ | 0.826 | 0.831 | 0.247 |
| N | 186 | 186 | 186 |

Notes: Cell entries are regression coefficients predicting each candidate’s vote share using the predictors listed in the first column with standard errors in parentheses.

***p<.001 **p<.01 *p<.05 +p<.10

Table SC11

*Name Order Effects in the 2016 Presidential Election in New Hampshire: Second/Third Versus First*

|  | Dependent Variable | | | | |
| --- | --- | --- | --- | --- | --- |
| Predictor | Vote share of the Democratic candidate (Hillary Clinton)  (1) | Vote share of the Republican candidate (Donald J. Trump)  (2) | Vote share of the other candidate (Gary Johnson, Libertarian)  (3) | Vote share of the other candidate (Jill Stein, Green)  (4) | Vote share of the other candidate (Roque “Rocky” De La Fuente, American Delta)  (5) |
| Listed second (vs. first) | -0.011 | -0.015 | 0.001 | -0.001 | -0.000 |
|  | (0.009) | (0.010) | (0.002) | (0.001) | (0.000) |
| Listed third (vs. first) | -0.019+ | -0.019+ | 0.001 | -0.001+ | -0.000+ |
|  | (0.010) | (0.010) | (0.002) | (0.001) | (0.000) |
| % Democratic registered voters | 0.444*** | -0.469*** | 0.016 | 0.010* | -0.001 |
|  | (0.059) | (0.058) | (0.011) | (0.005) | (0.002) |
| % Republican registered voters | -0.652*** | 0.684*** | 0.001 | -0.017** | -0.003 |
|  | (0.069) | (0.068) | (0.013) | (0.005) | (0.002) |
| Total number of votes (in 000s) | 0.004** | -0.003* | 0.000 | -0.000*** | -0.000 |
|  | (0.002) | (0.002) | (0.000) | (0.000) | (0.000) |
| Constant | 0.544*** | 0.420*** | 0.036*** | 0.014*** | 0.003** |
|  | (0.034) | (0.033) | (0.006) | (0.003) | (0.001) |
|  |  |  |  |  |  |
| R^2^ | 0.582 | 0.607 | 0.013 | 0.156 | 0.022 |
| N | 300 | 300 | 300 | 300 | 300 |

Notes: Cell entries are regression coefficients predicting each candidate’s vote share using the predictors listed in the first column with standard errors in parentheses.

***p<.001 **p<.01 *p<.05 +p<.10

Table SC12

*Name Order Effects in the 2016 U.S. Senate Election in New Hampshire: Second/Third Versus First*

|  | Dependent Variable | | | |
| --- | --- | --- | --- | --- |
| Predictor | Vote share of the Democratic candidate (Maggie Hassan)  (1) | Vote share of the Republican candidate (Kelly Ayotte)  (2) | Vote share of the other candidate (Brian Chabot, Libertarian)  (3) | Vote share of the other candidate (Aaron Day, Independent)  (4) |
| Listed second (vs. first) | -0.012+ | -0.014* | 0.001 | 0.001 |
|  | (0.007) | (0.006) | (0.001) | (0.002) |
| Listed third (vs. first) | -0.020** | -0.010 | -0.001 | -0.002 |
|  | (0.007) | (0.006) | (0.001) | (0.002) |
| % Democratic registered voters | 0.289*** | -0.282*** | 0.001 | 0.001 |
|  | (0.042) | (0.037) | (0.006) | (0.010) |
| % Republican registered voters | -0.779*** | 0.793*** | -0.014* | 0.003 |
|  | (0.049) | (0.044) | (0.007) | (0.012) |
| Total number of votes (in 000s) | 0.001 | -0.001 | 0.000 | -0.000 |
|  | (0.001) | (0.001) | (0.000) | (0.000) |
| Constant | 0.644*** | 0.325*** | 0.021*** | 0.029*** |
|  | (0.024) | (0.021) | (0.004) | (0.006) |
|  |  |  |  |  |
| R^2^ | 0.718 | 0.765 | 0.036 | 0.031 |
| N | 300 | 300 | 300 | 300 |

Notes: Cell entries are regression coefficients predicting each candidate’s vote share using the predictors listed in the first column with standard errors in parentheses.

***p<.001 **p<.01 *p<.05 +p<.10

Table SC13

*Name Order Effects in the 2016 Gubernatorial Election in New Hampshire: Second/Third Versus First*

|  | Dependent Variable | | |
| --- | --- | --- | --- |
| Predictor | Vote share of the Democratic candidate (Colin Van Ostern)  (1) | Vote share of the Republican candidate (Chris Sununu)  (2) | Vote share of the other candidate (Max Abramson, Libertarian)  (3) |
| Listed second (vs. first) | -0.011 | -0.012 | 0.006** |
|  | (0.009) | (0.009) | (0.002) |
| Listed third (vs. first) | -0.018+ | -0.020* | -0.005* |
|  | (0.009) | (0.009) | (0.002) |
| % Democratic registered voters | 0.339*** | -0.312*** | -0.032* |
|  | (0.057) | (0.053) | (0.013) |
| % Republican registered voters | -0.647*** | 0.715*** | -0.062*** |
|  | (0.066) | (0.062) | (0.015) |
| Total number of votes (in 000s) | -0.001 | 0.001 | -0.000 |
|  | (0.002) | (0.001) | (0.000) |
| Constant | 0.587*** | 0.362*** | 0.072*** |
|  | (0.032) | (0.030) | (0.008) |
|  |  |  |  |
| R^2^ | 0.549 | 0.596 | 0.116 |
| N | 300 | 300 | 300 |

Notes: Cell entries are regression coefficients predicting each candidate’s vote share using the predictors listed in the first column with standard errors in parentheses.

***p<.001 **p<.01 *p<.05 +p<.10

Table SC14

*Name Order Effects in the 2016 Congressional District 1 Election in New Hampshire: Second/Third Versus First*

|  | Dependent Variable | | | | |
| --- | --- | --- | --- | --- | --- |
| Predictor | Vote share of the Democratic candidate (Carol Shea-Porter)  (1) | Vote share of the Republican candidate (Frank Guinta)  (2) | Vote share of the other candidate (Robert Lombardo, Libertarian)  (3) | Vote share of the other candidate (Brendan Kelly, Independent)  (4) | Vote share of the other candidate (Shawn P. O’Connor, Independent)  (5) |
| Listed second (vs. first) | -0.007 | -0.005 | -0.000 | 0.002 | 0.010* |
|  | (0.008) | (0.008) | (0.001) | (0.001) | (0.004) |
| Listed third (vs. first) | -0.008 | -0.005 | -0.002+ | -0.001 | -0.008+ |
|  | (0.009) | (0.008) | (0.001) | (0.001) | (0.004) |
| % Democratic registered voters | 0.557*** | -0.495*** | -0.002 | -0.020+ | -0.046 |
|  | (0.072) | (0.068) | (0.010) | (0.011) | (0.037) |
| % Republican registered voters | -0.558*** | 0.630*** | -0.015 | -0.014 | -0.047 |
|  | (0.069) | (0.066) | (0.010) | (0.011) | (0.036) |
| Total number of votes (in 000s) | 0.000 | 0.000 | 0.000 | 0.000 | -0.001 |
|  | (0.001) | (0.001) | (0.000) | (0.000) | (0.001) |
| Constant | 0.473*** | 0.367*** | 0.020*** | 0.026*** | 0.127*** |
|  | (0.039) | (0.036) | (0.006) | (0.006) | (0.020) |
|  |  |  |  |  |  |
| R^2^ | 0.816 | 0.833 | 0.065 | 0.094 | 0.154 |
| N | 114 | 114 | 114 | 114 | 114 |

Notes: Cell entries are regression coefficients predicting each candidate’s vote share using the predictors listed in the first column with standard errors in parentheses.

***p<.001 **p<.01 *p<.05 +p<.10

Table SC15

*Name Order Effects in the 2016 Congressional District 2 Election in New Hampshire: Second/Third Versus First*

|  | Dependent Variable | | |
| --- | --- | --- | --- |
| Predictor | Vote share of the Democratic candidate (Ann McLane Kuster)  (1) | Vote share of the Republican candidate (Jim Lawrence)  (2) | Vote share of the other candidate (John J. Babiarz, Independent)  (3) |
| Listed second (vs. first) | -0.008 | -0.017+ | 0.007* |
|  | (0.010) | (0.009) | (0.004) |
| Listed third (vs. first) | -0.023* | -0.025** | -0.005 |
|  | (0.011) | (0.009) | (0.003) |
| % Democratic registered voters | 0.353*** | -0.335*** | -0.026 |
|  | (0.056) | (0.047) | (0.019) |
| % Republican registered voters | -0.659*** | 0.723*** | -0.062* |
|  | (0.071) | (0.059) | (0.024) |
| Total number of votes (in 000s) | 0.001 | -0.001 | 0.000 |
|  | (0.002) | (0.002) | (0.001) |
| Constant | 0.599*** | 0.352*** | 0.074*** |
|  | (0.033) | (0.028) | (0.011) |
|  |  |  |  |
| R^2^ | 0.638 | 0.731 | 0.087 |
| N | 186 | 186 | 186 |

Notes: Cell entries are regression coefficients predicting each candidate’s vote share using the predictors listed in the first column with standard errors in parentheses.

***p<.001 **p<.01 *p<.05 +p<.10

Table SC16

*Name Order Effects in the 2012 Presidential Election in New Hampshire: First Versus Later (With Weights)*

|  | Dependent Variable | | | |
| --- | --- | --- | --- | --- |
| Predictor | Vote share of the Democratic candidate (Barack Obama)  (1) | Vote share of the Republican candidate (Mitt Romney)  (2) | Vote share of the other candidate (Gary Johnson, Libertarian)  (3) | Vote share of the other candidate (Virgil Goode, Constitution)  (4) |
| Listed first (vs. later) | 0.002 | -0.001 | 0.000 | 0.000 |
|  | (0.005) | (0.005) | (0.001) | (0.000) |
| % Democratic registered voters | 0.600*** | -0.571*** | -0.021** | -0.008** |
|  | (0.053) | (0.052) | (0.007) | (0.003) |
| % Republican registered voters | -0.631*** | 0.664*** | -0.029*** | -0.006+ |
|  | (0.060) | (0.060) | (0.008) | (0.003) |
| Total number of votes (in 000s) | -0.006*** | 0.006*** | -0.000 | -0.000** |
|  | (0.001) | (0.001) | (0.000) | (0.000) |
| Constant | 0.577*** | 0.391*** | 0.027*** | 0.006** |
|  | (0.029) | (0.029) | (0.004) | (0.002) |
|  |  |  |  |  |
| R^2^ | 0.789 | 0.794 | 0.085 | 0.064 |
| N | 300 | 300 | 300 | 300 |

Notes: Cell entries are regression coefficients predicting each candidate’s vote share using the predictors listed in the first column with standard errors in parentheses.

***p<.001 **p<.01 *p<.05 +p<.10

Table SC17

*Name Order Effects in the 2012 Presidential Election in New Hampshire: Second/Third Versus First (With Weights)*

|  | Dependent Variable | | | |
| --- | --- | --- | --- | --- |
| Predictor | Vote share of the Democratic candidate (Barack Obama)  (1) | Vote share of the Republican candidate (Mitt Romney)  (2) | Vote share of the other candidate (Gary Johnson, Libertarian)  (3) | Vote share of the other candidate (Virgil Goode, Constitution)  (4) |
| Listed second (vs. first) | -0.000 | 0.003 | 0.000 | -0.000 |
|  | (0.006) | (0.005) | (0.001) | (0.000) |
| Listed third (vs. first) | -0.003 | -0.003 | -0.001 | 0.000 |
|  | (0.006) | (0.006) | (0.001) | (0.000) |
| % Democratic registered voters | 0.600*** | -0.572*** | -0.021** | -0.008** |
|  | (0.053) | (0.052) | (0.007) | (0.003) |
| % Republican registered voters | -0.632*** | 0.664*** | -0.029*** | -0.007+ |
|  | (0.060) | (0.060) | (0.008) | (0.004) |
| Total number of votes (in 000s) | -0.006*** | 0.006*** | -0.000 | -0.000** |
|  | (0.001) | (0.001) | (0.000) | (0.000) |
| Constant | 0.579*** | 0.390*** | 0.027*** | 0.006** |
|  | (0.029) | (0.029) | (0.004) | (0.002) |
|  |  |  |  |  |
| R^2^ | 0.789 | 0.795 | 0.088 | 0.067 |
| N | 300 | 300 | 300 | 300 |

Notes: Cell entries are regression coefficients predicting each candidate’s vote share using the predictors listed in the first column with standard errors in parentheses.

***p<.001 **p<.01 *p<.05 +p<.10

Table SC18

*Name Order Effects in the 2012 Presidential Election in New Hampshire: Name Order (With Weights)*

|  | Dependent Variable | | | |
| --- | --- | --- | --- | --- |
| Predictor | Vote share of the Democratic candidate (Barack Obama)  (1) | Vote share of the Republican candidate (Mitt Romney)  (2) | Vote share of the other candidate (Gary Johnson, Libertarian)  (3) | Vote share of the other candidate (Virgil Goode, Constitution)  (4) |
| Name order | -0.002 | -0.001 | -0.000 | 0.000 |
|  | (0.003) | (0.003) | (0.000) | (0.000) |
| % Democratic registered voters | 0.600*** | -0.572*** | -0.021** | -0.008** |
|  | (0.053) | (0.052) | (0.007) | (0.003) |
| % Republican registered voters | -0.632*** | 0.665*** | -0.029*** | -0.006+ |
|  | (0.060) | (0.060) | (0.008) | (0.004) |
| Total number of votes (in 000s) | -0.006*** | 0.006*** | -0.000 | -0.000** |
|  | (0.001) | (0.001) | (0.000) | (0.000) |
| Constant | 0.578*** | 0.391*** | 0.027*** | 0.006** |
|  | (0.029) | (0.029) | (0.004) | (0.002) |
|  |  |  |  |  |
| R^2^ | 0.789 | 0.794 | 0.086 | 0.064 |
| N | 300 | 300 | 300 | 300 |

Notes: Cell entries are regression coefficients predicting each candidate’s vote share using the predictors listed in the first column with standard errors in parentheses.

***p<.001 **p<.01 *p<.05 +p<.10

Table SC19

*Name Order Effects in the 2012 Presidential Election in New Hampshire: Name Order and Name Order Squared (With Weights)*

|  | Dependent Variable | | | |
| --- | --- | --- | --- | --- |
| Predictor | Vote share of the Democratic candidate (Barack Obama)  (1) | Vote share of the Republican candidate (Mitt Romney)  (2) | Vote share of the other candidate (Gary Johnson, Libertarian)  (3) | Vote share of the other candidate (Virgil Goode, Constitution)  (4) |
| Name order | -0.002 | -0.002 | -0.000 | -0.000 |
|  | (0.003) | (0.003) | (0.000) | (0.000) |
| Name order squared | -0.001 | -0.005 | -0.001 | -0.001 |
|  | (0.005) | (0.005) | (0.001) | (0.001) |
| % Democratic registered voters | 0.600*** | -0.572*** | -0.021** | -0.021** |
|  | (0.053) | (0.052) | (0.007) | (0.007) |
| % Republican registered voters | -0.632*** | 0.664*** | -0.029*** | -0.029*** |
|  | (0.060) | (0.060) | (0.008) | (0.008) |
| Total number of votes (in 000s) | -0.006*** | 0.006*** | -0.000 | -0.000 |
|  | (0.001) | (0.001) | (0.000) | (0.000) |
| Constant | 0.579*** | 0.394*** | 0.027*** | 0.027*** |
|  | (0.029) | (0.029) | (0.004) | (0.004) |
|  |  |  |  |  |
| R^2^ | 0.789 | 0.795 | 0.088 | 0.088 |
| N | 300 | 300 | 300 | 300 |

Notes: Cell entries are regression coefficients predicting each candidate’s vote share using the predictors listed in the first column with standard errors in parentheses.

***p<.001 **p<.01 *p<.05 +p<.10

Table SC20

*Name Order Effects in the 2012 Gubernatorial Election in New Hampshire: First Versus Later (With Weights)*

|  | Dependent Variable | | |
| --- | --- | --- | --- |
| Predictor | Vote share of the Democratic candidate (Maggie Hassan)  (1) | Vote share of the Republican candidate (Ovide Lamontagne)  (2) | Vote share of the other candidate (John J. Babiarz, Libertarian)  (3) |
| Listed first (vs. later) | 0.001 | 0.001 | -0.000 |
|  | (0.005) | (0.005) | (0.002) |
| % Democratic registered voters | 0.628*** | -0.590*** | -0.037* |
|  | (0.064) | (0.068) | (0.014) |
| % Republican registered voters | -0.535*** | 0.593*** | -0.059*** |
|  | (0.068) | (0.073) | (0.014) |
| Total number of votes (in 000s) | -0.004*** | 0.005*** | -0.000 |
|  | (0.001) | (0.001) | (0.000) |
| Constant | 0.559*** | 0.385*** | 0.056*** |
|  | (0.035) | (0.037) | (0.008) |
|  |  |  |  |
| R^2^ | 0.768 | 0.776 | 0.068 |
| N | 300 | 300 | 300 |

Notes: Cell entries are regression coefficients predicting each candidate’s vote share using the predictors listed in the first column with standard errors in parentheses.

***p<.001 **p<.01 *p<.05 +p<.10

Table SC21

*Name Order Effects in the 2012 Gubernatorial Election in New Hampshire: Second/Third Versus First (With Weights)*

|  | Dependent Variable | | |
| --- | --- | --- | --- |
| Predictor | Vote share of the Democratic candidate (Maggie Hassan)  (1) | Vote share of the Republican candidate (Ovide Lamontagne)  (2) | Vote share of the other candidate (John J. Babiarz, Libertarian)  (3) |
| Listed second (vs. first) | 0.001 | 0.000 | 0.004* |
|  | (0.005) | (0.005) | (0.002) |
| Listed third (vs. first) | -0.002 | -0.003 | -0.003+ |
|  | (0.006) | (0.006) | (0.002) |
| % Democratic registered voters | 0.627*** | -0.591*** | -0.036* |
|  | (0.064) | (0.068) | (0.014) |
| % Republican registered voters | -0.536*** | 0.593*** | -0.055*** |
|  | (0.068) | (0.073) | (0.014) |
| Total number of votes (in 000s) | -0.005*** | 0.005*** | -0.000 |
|  | (0.001) | (0.001) | (0.000) |
| Constant | 0.560*** | 0.386*** | 0.055*** |
|  | (0.035) | (0.038) | (0.008) |
|  |  |  |  |
| R^2^ | 0.768 | 0.776 | 0.113 |
| N | 300 | 300 | 300 |

Notes: Cell entries are regression coefficients predicting each candidate’s vote share using the predictors listed in the first column with standard errors in parentheses.

***p<.001 **p<.01 *p<.05 +p<.10

Table SC22

*Name Order Effects in the 2012 Gubernatorial Election in New Hampshire: Name Order (With Weights)*

|  | Dependent Variable | | |
| --- | --- | --- | --- |
| Predictor | Vote share of the Democratic candidate (Maggie Hassan)  (1) | Vote share of the Republican candidate (Ovide Lamontagne)  (2) | Vote share of the other candidate (John J. Babiarz, Libertarian)  (3) |
| Name order | -0.001 | -0.001 | -0.002+ |
|  | (0.003) | (0.003) | (0.001) |
| % Democratic registered voters | 0.627*** | -0.591*** | -0.037* |
|  | (0.064) | (0.068) | (0.014) |
| % Republican registered voters | -0.536*** | 0.594*** | -0.056*** |
|  | (0.068) | (0.073) | (0.014) |
| Total number of votes (in 000s) | -0.004*** | 0.005*** | -0.000 |
|  | (0.001) | (0.001) | (0.000) |
| Constant | 0.559*** | 0.385*** | 0.055*** |
|  | (0.035) | (0.037) | (0.008) |
|  |  |  |  |
| R^2^ | 0.768 | 0.776 | 0.077 |
| N | 300 | 300 | 300 |

Notes: Cell entries are regression coefficients predicting each candidate’s vote share using the predictors listed in the first column with standard errors in parentheses.

***p<.001 **p<.01 *p<.05 +p<.10

Table SC23

*Name Order Effects in the 2012 Gubernatorial Election in New Hampshire: Name Order and Name Order Squared (With Weights)*

|  | Dependent Variable | | |
| --- | --- | --- | --- |
| Predictor | Vote share of the Democratic candidate (Maggie Hassan)  (1) | Vote share of the Republican candidate (Ovide Lamontagne)  (2) | Vote share of the other candidate (John J. Babiarz, Libertarian)  (3) |
| Name order | -0.001 | -0.002 | -0.001+ |
|  | (0.003) | (0.003) | (0.001) |
| Name order squared | -0.002 | -0.002 | -0.005*** |
|  | (0.005) | (0.005) | (0.001) |
| % Democratic registered voters | 0.627*** | -0.591*** | -0.036* |
|  | (0.064) | (0.068) | (0.014) |
| % Republican registered voters | -0.536*** | 0.593*** | -0.055*** |
|  | (0.068) | (0.073) | (0.014) |
| Total number of votes (in 000s) | -0.005*** | 0.005*** | -0.000 |
|  | (0.001) | (0.001) | (0.000) |
| Constant | 0.561*** | 0.386*** | 0.058*** |
|  | (0.035) | (0.037) | (0.008) |
|  |  |  |  |
| R^2^ | 0.768 | 0.776 | 0.113 |
| N | 300 | 300 | 300 |

Notes: Cell entries are regression coefficients predicting each candidate’s vote share using the predictors listed in the first column with standard errors in parentheses.

***p<.001 **p<.01 *p<.05 +p<.10

Table SC24

*Name Order Effects in the 2012 Congressional District 1 Election in New Hampshire: First Versus Later (With Weights)*

|  | Dependent Variable | | |
| --- | --- | --- | --- |
| Predictor | Vote share of the Democratic candidate (Carol Shea-Porter)  (1) | Vote share of the Republican candidate (Frank C. Guinta)  (2) | Vote share of the other candidate (Brendan Kelly, Libertarian)  (3) |
| Listed first (vs. later) | -0.002 | -0.007 | -0.001 |
|  | (0.009) | (0.007) | (0.003) |
| % Democratic registered voters | 0.474*** | -0.423*** | -0.045+ |
|  | (0.095) | (0.088) | (0.023) |
| % Republican registered voters | -0.617*** | 0.674*** | -0.058* |
|  | (0.104) | (0.103) | (0.025) |
| Total number of votes (in 000s) | -0.002** | 0.002* | 0.000 |
|  | (0.001) | (0.001) | (0.000) |
| Constant | 0.575*** | 0.355*** | 0.072*** |
|  | (0.055) | (0.052) | (0.013) |
|  |  |  |  |
| R^2^ | 0.781 | 0.817 | 0.040 |
| N | 114 | 114 | 114 |

Notes: Cell entries are regression coefficients predicting each candidate’s vote share using the predictors listed in the first column with standard errors in parentheses.

***p<.001 **p<.01 *p<.05 +p<.10

Table SC25

*Name Order Effects in the 2012 Congressional District 1 Election in New Hampshire: Second/Third Versus First (With Weights)*

|  | Dependent Variable | | |
| --- | --- | --- | --- |
| Predictor | Vote share of the Democratic candidate (Carol Shea-Porter)  (1) | Vote share of the Republican candidate (Frank C. Guinta)  (2) | Vote share of the other candidate (Brendan Kelly, Libertarian)  (3) |
| Listed second (vs. first) | 0.009 | 0.012+ | 0.008* |
|  | (0.009) | (0.007) | (0.003) |
| Listed third (vs. first) | -0.005 | 0.001 | -0.005+ |
|  | (0.010) | (0.009) | (0.003) |
| % Democratic registered voters | 0.468*** | -0.423*** | -0.040* |
|  | (0.095) | (0.087) | (0.023) |
| % Republican registered voters | -0.626*** | 0.676*** | -0.051* |
|  | (0.104) | (0.099) | (0.025) |
| Total number of votes (in 000s) | -0.002* | 0.002+ | 0.000 |
|  | (0.001) | (0.001) | (0.000) |
| Constant | 0.579*** | 0.347*** | 0.067*** |
|  | (0.054) | (0.050) | (0.014) |
|  |  |  |  |
| R^2^ | 0.786 | 0.820 | 0.186 |
| N | 114 | 114 | 114 |

Notes: Cell entries are regression coefficients predicting each candidate’s vote share using the predictors listed in the first column with standard errors in parentheses.

***p<.001 **p<.01 *p<.05 +p<.10

Table SC26

*Name Order Effects in the 2012 Congressional District 1 Election in New Hampshire: Name Order (With Weights)*

|  | Dependent Variable | | |
| --- | --- | --- | --- |
| Predictor | Vote share of the Democratic candidate (Carol Shea-Porter)  (1) | Vote share of the Republican candidate (Frank C. Guinta)  (2) | Vote share of the other candidate (Brendan Kelly, Libertarian)  (3) |
| Name order | -0.003 | 0.001 | -0.003+ |
|  | (0.005) | (0.005) | (0.001) |
| % Democratic registered voters | 0.476*** | -0.429*** | -0.042+ |
|  | (0.093) | (0.089) | (0.023) |
| % Republican registered voters | -0.623*** | 0.675*** | -0.053* |
|  | (0.104) | (0.103) | (0.025) |
| Total number of votes (in 000s) | -0.002* | 0.002*\+ | 0.000 |
|  | (0.001) | (0.001) | (0.000) |
| Constant | 0.576*** | 0.354*** | 0.069*** |
|  | (0.054) | (0.052) | (0.013) |
|  |  |  |  |
| R^2^ | 0.782 | 0.815 | 0.062 |
| N | 114 | 114 | 114 |

Notes: Cell entries are regression coefficients predicting each candidate’s vote share using the predictors listed in the first column with standard errors in parentheses.

***p<.001 **p<.01 *p<.05 +p<.10

Table SC27

*Name Order Effects in the 2012 Congressional District 1 Election in New Hampshire: Name Order and Name Order Squared (With Weights)*

|  | Dependent Variable | | |
| --- | --- | --- | --- |
| Predictor | Vote share of the Democratic candidate (Carol Shea-Porter)  (1) | Vote share of the Republican candidate (Frank C. Guinta)  (2) | Vote share of the other candidate (Brendan Kelly, Libertarian)  (3) |
| Name order | -0.003 | 0.001 | -0.003+ |
|  | (0.005) | (0.005) | (0.001) |
| Name order squared | -0.012 | -0.012+ | -0.011*** |
|  | (0.007) | (0.007) | (0.003) |
| % Democratic registered voters | 0.468*** | -0.423*** | -0.040+ |
|  | (0.095) | (0.087) | (0.023) |
| % Republican registered voters | -0.626*** | 0.676*** | -0.051* |
|  | (0.104) | (0.099) | (0.025) |
| Total number of votes (in 000s) | -0.002** | 0.002* | 0.000 |
|  | (0.001) | (0.001) | (0.000) |
| Constant | 0.588*** | 0.359*** | 0.076*** |
|  | (0.056) | (0.051) | (0.013) |
|  |  |  |  |
| R^2^ | 0.786 | 0.820 | 0.186 |
| N | 114 | 114 | 114 |

Notes: Cell entries are regression coefficients predicting each candidate’s vote share using the predictors listed in the first column with standard errors in parentheses.

***p<.001 **p<.01 *p<.05 +p<.10

Table SC28

*Name Order Effects in the 2012 Congressional District 2 Election in New Hampshire: First Versus Later (With Weights)*

|  | Dependent Variable | | |
| --- | --- | --- | --- |
| Predictor | Vote share of the Democratic candidate (Ann McLane Kuster)  (1) | Vote share of the Republican candidate (Charles Bass)  (2) | Vote share of the other candidate (Hardy Macia, Libertarian)  (3) |
| Listed first (vs. later) | 0.007 | 0.001 | 0.001 |
|  | (0.005) | (0.005) | (0.002) |
| % Democratic registered voters | 0.734*** | -0.652*** | -0.084*** |
|  | (0.049) | (0.048) | (0.024) |
| % Republican registered voters | -0.513*** | 0.558*** | -0.049+ |
|  | (0.052) | (0.050) | (0.027) |
| Total number of votes (in 000s) | -0.007*** | 0.006*** | 0.001 |
|  | (0.001) | (0.001) | (0.000) |
| Constant | 0.476*** | 0.444*** | 0.079*** |
|  | (0.024) | (0.024) | (0.013) |
|  |  |  |  |
| R^2^ | 0.843 | 0.858 | 0.086 |
| N | 186 | 186 | 186 |

Notes: Cell entries are regression coefficients predicting each candidate’s vote share using the predictors listed in the first column with standard errors in parentheses.

***p<.001 **p<.01 *p<.05 +p<.10

Table SC29

*Name Order Effects in the 2012 Congressional District 2 Election in New Hampshire: Second/Third Versus First (With Weights)*

|  | Dependent Variable | | |
| --- | --- | --- | --- |
| Predictor | Vote share of the Democratic candidate (Ann McLane Kuster)  (1) | Vote share of the Republican candidate (Charles Bass)  (2) | Vote share of the other candidate (Hardy Macia, Libertarian)  (3) |
| Listed second (vs. first) | -0.006 | -0.003 | 0.007** |
|  | (0.006) | (0.005) | (0.003) |
| Listed third (vs. first) | -0.007 | 0.001 | -0.009*** |
|  | (0.006) | (0.005) | (0.002) |
| % Democratic registered voters | 0.734*** | -0.652*** | -0.080*** |
|  | (0.050) | (0.049) | (0.022) |
| % Republican registered voters | -0.514*** | 0.558*** | -0.037 |
|  | (0.054) | (0.050) | (0.024) |
| Total number of votes (in 000s) | -0.007*** | 0.006*** | 0.000 |
|  | (0.001) | (0.001) | (0.000) |
| Constant | 0.475*** | 0.446*** | 0.067*** |
|  | (0.024) | (0.024) | (0.012) |
|  |  |  |  |
| R^2^ | 0.843 | 0.858 | 0.253 |
| N | 186 | 186 | 186 |

Notes: Cell entries are regression coefficients predicting each candidate’s vote share using the predictors listed in the first column with standard errors in parentheses.

***p<.001 **p<.01 *p<.05 +p<.10

Table SC30

*Name Order Effects in the 2012 Congressional District 2 Election in New Hampshire: Name Order (With Weights)*

|  | Dependent Variable | | |
| --- | --- | --- | --- |
| Predictor | Vote share of the Democratic candidate (Ann McLane Kuster)  (1) | Vote share of the Republican candidate (Charles Bass)  (2) | Vote share of the other candidate (Hardy Macia, Libertarian)  (3) |
| Name order | -0.003 | 0.000 | -0.005*** |
|  | (0.003) | (0.003) | (0.001) |
| % Democratic registered voters | 0.734*** | -0.653*** | -0.083*** |
|  | (0.049) | (0.049) | (0.024) |
| % Republican registered voters | -0.514*** | 0.558*** | -0.041 |
|  | (0.053) | (0.050) | (0.026) |
| Total number of votes (in 000s) | -0.007*** | 0.006*** | 0.000 |
|  | (0.001) | (0.001) | (0.000) |
| Constant | 0.478*** | 0.445*** | 0.077*** |
|  | (0.025) | (0.024) | (0.013) |
|  |  |  |  |
| R^2^ | 0.843 | 0.858 | 0.140 |
| N | 186 | 186 | 186 |

Notes: Cell entries are regression coefficients predicting each candidate’s vote share using the predictors listed in the first column with standard errors in parentheses.

***p<.001 **p<.01 *p<.05 +p<.10

Table SC31

*Name Order Effects in the 2012 Congressional District 2 Election in New Hampshire: Name Order and Name Order Squared (With Weights)*

|  | Dependent Variable | | |
| --- | --- | --- | --- |
| Predictor | Vote share of the Democratic candidate (Ann McLane Kuster)  (1) | Vote share of the Republican candidate (Charles Bass)  (2) | Vote share of the other candidate (Hardy Macia, Libertarian)  (3) |
| Name order | -0.004 | 0.000 | -0.005*** |
|  | (0.003) | (0.003) | (0.001) |
| Name order squared | 0.003 | 0.004 | -0.012*** |
|  | (0.006) | (0.004) | (0.002) |
| % Democratic registered voters | 0.734*** | -0.652*** | -0.080*** |
|  | (0.050) | (0.049) | (0.022) |
| % Republican registered voters | -0.514*** | 0.558*** | -0.037 |
|  | (0.054) | (0.050) | (0.024) |
| Total number of votes (in 000s) | -0.007*** | 0.006*** | 0.000 |
|  | (0.001) | (0.001) | (0.000) |
| Constant | 0.476*** | 0.442*** | 0.083*** |
|  | (0.026) | (0.024) | (0.012) |
|  |  |  |  |
| R^2^ | 0.843 | 0.858 | 0.253 |
| N | 186 | 186 | 186 |

Notes: Cell entries are regression coefficients predicting each candidate’s vote share using the predictors listed in the first column with standard errors in parentheses.

***p<.001 **p<.01 *p<.05 +p<.10

Table SC32

*Name Order Effects in the 2016 Presidential Election in New Hampshire: First Versus Later (With Weights)*

|  | Dependent Variable | | | | |
| --- | --- | --- | --- | --- | --- |
| Predictor | Vote share of the Democratic candidate (Hillary Clinton)  (1) | Vote share of the Republican candidate (Donald J. Trump)  (2) | Vote share of the other candidate (Gary Johnson, Libertarian)  (3) | Vote share of the other candidate (Jill Stein, Green)  (4) | Vote share of the other candidate (Roque “Rocky” De La Fuente, American Delta)  (5) |
| Listed first (vs. later) | 0.011 | 0.014+ | -0.001 | 0.001 | 0.000 |
|  | (0.007) | (0.008) | (0.001) | (0.001) | (0.000) |
| % Democratic registered voters | 0.510*** | -0.524*** | 0.007 | 0.005 | -0.002 |
|  | (0.118) | (0.109) | (0.013) | (0.006) | (0.001) |
| % Republican registered voters | -0.693*** | 0.718*** | 0.006 | -0.018*** | -0.003* |
|  | (0.087) | (0.086) | (0.015) | (0.005) | (0.002) |
| Total number of votes (in 000s) | 0.003** | -0.003+ | 0.000 | -0.000*** | -0.000+ |
|  | (0.002) | (0.002) | (0.000) | (0.000) | (0.000) |
| Constant | 0.525*** | 0.409*** | 0.038*** | 0.015*** | 0.003*** |
|  | (0.053) | (0.052) | (0.007) | (0.003) | (0.001) |
|  |  |  |  |  |  |
| R^2^ | 0.642 | 0.663 | 0.003 | 0.154 | 0.023 |
| N | 300 | 300 | 300 | 300 | 300 |

Notes: Cell entries are regression coefficients predicting each candidate’s vote share using the predictors listed in the first column with standard errors in parentheses.

***p<.001 **p<.01 *p<.05 +p<.10

Table SC33

*Name Order Effects in the 2016 Presidential Election in New Hampshire: New Hampshire: Second/Third Versus First (With Weights)*

|  | Dependent Variable | | | | |
| --- | --- | --- | --- | --- | --- |
| Predictor | Vote share of the Democratic candidate (Hillary Clinton)  (1) | Vote share of the Republican candidate (Donald J. Trump)  (2) | Vote share of the other candidate (Gary Johnson, Libertarian)  (3) | Vote share of the other candidate (Jill Stein, Green)  (4) | Vote share of the other candidate (Roque “Rocky” De La Fuente, American Delta)  (5) |
| Listed second (vs. first) | -0.008 | -0.011 | 0.001 | -0.001 | -0.000 |
|  | (0.008) | (0.009) | (0.001) | (0.001) | (0.000) |
| Listed third (vs. first) | -0.014 | -0.017* | 0.000 | -0.001 | -0.000 |
|  | (0.009) | (0.009) | (0.001) | (0.001) | (0.000) |
| % Democratic registered voters | 0.512*** | -0.522*** | 0.006 | 0.005 | -0.002 |
|  | (0.117) | (0.109) | (0.013) | (0.006) | (0.001) |
| % Republican registered voters | -0.691*** | 0.722*** | 0.005 | -0.018*** | -0.003* |
|  | (0.086) | (0.086) | (0.015) | (0.005) | (0.002) |
| Total number of votes (in 000s) | 0.003* | -0.003+ | 0.000 | -0.000*** | -0.000+ |
|  | (0.002) | (0.002) | (0.000) | (0.000) | (0.000) |
| Constant | 0.520*** | 0.404*** | 0.038*** | 0.015*** | 0.003*** |
|  | (0.051) | (0.053) | (0.007) | (0.003) | (0.001) |
|  |  |  |  |  |  |
| R^2^ | 0.643 | 0.664 | 0.005 | 0.154 | 0.023 |
| N | 300 | 300 | 300 | 300 | 300 |

Notes: Cell entries are regression coefficients predicting each candidate’s vote share using the predictors listed in the first column with standard errors in parentheses.

***p<.001 **p<.01 *p<.05 +p<.10

Table SC34

*Name Order Effects in the 2016 Presidential Election in New Hampshire: Name Order (With Weights)*

|  | Dependent Variable | | | | |
| --- | --- | --- | --- | --- | --- |
| Predictor | Vote share of the Democratic candidate (Hillary Clinton)  (1) | Vote share of the Republican candidate (Donald J. Trump)  (2) | Vote share of the other candidate (Gary Johnson, Libertarian)  (3) | Vote share of the other candidate (Jill Stein, Green)  (4) | Vote share of the other candidate (Roque “Rocky” De La Fuente, American Delta)  (5) |
| Name order | -0.007 | -0.009* | 0.000 | -0.000 | -0.000 |
|  | (0.005) | (0.004) | (0.001) | (0.000) | (0.000) |
| % Democratic registered voters | 0.512*** | -0.521*** | 0.007 | 0.005 | -0.002 |
|  | (0.117) | (0.109) | (0.013) | (0.006) | (0.001) |
| % Republican registered voters | -0.691*** | 0.722*** | 0.005 | -0.019*** | -0.003* |
|  | (0.087) | (0.086) | (0.015) | (0.005) | (0.002) |
| Total number of votes (in 000s) | 0.003** | -0.003+ | 0.000 | -0.000*** | -0.000+ |
|  | (0.002) | (0.002) | (0.000) | (0.000) | (0.000) |
| Constant | 0.527*** | 0.412*** | 0.038*** | 0.015*** | 0.003*** |
|  | (0.053) | (0.052) | (0.007) | (0.003) | (0.001) |
|  |  |  |  |  |  |
| R^2^ | 0.643 | 0.664 | 0.002 | 0.153 | 0.023 |
| N | 300 | 300 | 300 | 300 | 300 |

Notes: Cell entries are regression coefficients predicting each candidate’s vote share using the predictors listed in the first column with standard errors in parentheses.

***p<.001 **p<.01 *p<.05 +p<.10

Table SC35

*Name Order Effects in the 2016 Presidential Election in New Hampshire: Name Order and Name Order Squared (With Weights)*

|  | Dependent Variable | | | | |
| --- | --- | --- | --- | --- | --- |
| Predictor | Vote share of the Democratic candidate (Hillary Clinton)  (1) | Vote share of the Republican candidate (Donald J. Trump)  (2) | Vote share of the other candidate (Gary Johnson, Libertarian)  (3) | Vote share of the other candidate (Jill Stein, Green)  (4) | Vote share of the other candidate (Roque “Rocky” De La Fuente, American Delta)  (5) |
| Name order | -0.007 | -0.009* | 0.000 | -0.000 | -0.000 |
|  | (0.005) | (0.004) | (0.001) | (0.000) | (0.000) |
| Name order squared | 0.001 | 0.003 | -0.001 | 0.000 | 0.000 |
|  | (0.007) | (0.007) | (0.001) | (0.000) | (0.000) |
| % Democratic registered voters | 0.512*** | -0.522*** | 0.006 | 0.005 | -0.002 |
|  | (0.117) | (0.109) | (0.013) | (0.006) | (0.001) |
| % Republican registered voters | -0.691*** | 0.722*** | 0.005 | -0.018*** | -0.003* |
|  | (0.086) | (0.086) | (0.015) | (0.005) | (0.002) |
| Total number of votes (in 000s) | 0.003** | -0.003+ | 0.000 | -0.000*** | -0.000+ |
|  | (0.002) | (0.002) | (0.000) | (0.000) | (0.000) |
| Constant | 0.527*** | 0.410*** | 0.039*** | 0.015*** | 0.003** |
|  | (0.053) | (0.052) | (0.007) | (0.003) | (0.001) |
|  |  |  |  |  |  |
| R^2^ | 0.643 | 0.664 | 0.005 | 0.154 | 0.023 |
| N | 300 | 300 | 300 | 300 | 300 |

Notes: Cell entries are regression coefficients predicting each candidate’s vote share using the predictors listed in the first column with standard errors in parentheses.

***p<.001 **p<.01 *p<.05 +p<.10

Table SC36

*Name Order Effects in the 2016 U.S. Senate Election in New Hampshire: First Versus Later (With Weights)*

|  | Dependent Variable | | | |
| --- | --- | --- | --- | --- |
| Predictor | Vote share of the Democratic candidate (Maggie Hassan)  (1) | Vote share of the Republican candidate (Kelly Ayotte)  (2) | Vote share of the other candidate (Brian Chabot, Libertarian)  (3) | Vote share of the other candidate (Aaron Day, Independent)  (4) |
| Listed first (vs. later) | 0.010* | 0.010* | -0.000 | 0.000 |
|  | (0.005) | (0.005) | (0.001) | (0.001) |
| % Democratic registered voters | 0.387*** | -0.360*** | -0.004 | -0.025* |
|  | (0.090) | (0.083) | (0.008) | (0.010) |
| % Republican registered voters | -0.753*** | 0.785*** | -0.014+ | -0.010 |
|  | (0.069) | (0.066) | (0.008) | (0.015) |
| Total number of votes (in 000s) | 0.001 | -0.001 | -0.000 | -0.000 |
|  | (0.001) | (0.001) | (0.000) | (0.000) |
| Constant | 0.596*** | 0.338*** | 0.023*** | 0.035*** |
|  | (0.042) | (0.040) | (0.004) | (0.007) |
|  |  |  |  |  |
| R^2^ | 0.769 | 0.811 | 0.019 | 0.028 |
| N | 300 | 300 | 300 | 300 |

Notes: Cell entries are regression coefficients predicting each candidate’s vote share using the predictors listed in the first column with standard errors in parentheses.

***p<.001 **p<.01 *p<.05 +p<.10

Table SC37

*Name Order Effects in the 2016 U.S. Senate Election in New Hampshire: New Hampshire: Second/Third Versus First (With Weights)*

|  | Dependent Variable | | | |
| --- | --- | --- | --- | --- |
| Predictor | Vote share of the Democratic candidate (Maggie Hassan)  (1) | Vote share of the Republican candidate (Kelly Ayotte)  (2) | Vote share of the other candidate (Brian Chabot, Libertarian)  (3) | Vote share of the other candidate (Aaron Day, Independent)  (4) |
| Listed second (vs. first) | -0.007 | -0.011* | 0.001 | 0.002 |
|  | (0.005) | (0.005) | (0.001) | (0.001) |
| Listed third (vs. first) | -0.014* | -0.009+ | -0.000 | -0.002 |
|  | (0.006) | (0.005) | (0.001) | (0.001) |
| % Democratic registered voters | 0.388*** | -0.361*** | -0.005 | -0.026* |
|  | (0.089) | (0.084) | (0.008) | (0.010) |
| % Republican registered voters | -0.751*** | 0.783*** | -0.015+ | -0.013 |
|  | (0.068) | (0.066) | (0.008) | (0.015) |
| Total number of votes (in 000s) | 0.001 | -0.001 | -0.000 | -0.000 |
|  | (0.001) | (0.001) | (0.000) | (0.000) |
| Constant | 0.605*** | 0.348*** | 0.023*** | 0.037*** |
|  | (0.043) | (0.039) | (0.004) | (0.007) |
|  |  |  |  |  |
| R^2^ | 0.770 | 0.812 | 0.030 | 0.044 |
| N | 300 | 300 | 300 | 300 |

Notes: Cell entries are regression coefficients predicting each candidate’s vote share using the predictors listed in the first column with standard errors in parentheses.

***p<.001 **p<.01 *p<.05 +p<.10

Table SC38

*Name Order Effects in the 2016 U.S. Senate Election in New Hampshire: Name Order (With Weights)*

|  | Dependent Variable | | | |
| --- | --- | --- | --- | --- |
| Predictor | Vote share of the Democratic candidate (Maggie Hassan)  (1) | Vote share of the Republican candidate (Kelly Ayotte)  (2) | Vote share of the other candidate (Brian Chabot, Libertarian)  (3) | Vote share of the other candidate (Aaron Day, Independent)  (4) |
| Name order | -0.007* | -0.004+ | -0.000 | -0.001 |
|  | (0.003) | (0.003) | (0.000) | (0.001) |
| % Democratic registered voters | 0.388*** | -0.358*** | -0.004 | -0.025* |
|  | (0.089) | (0.084) | (0.008) | (0.010) |
| % Republican registered voters | -0.751*** | 0.785*** | -0.015+ | -0.012 |
|  | (0.068) | (0.066) | (0.008) | (0.015) |
| Total number of votes (in 000s) | 0.001 | -0.001 | -0.000 | -0.000 |
|  | (0.001) | (0.001) | (0.000) | (0.000) |
| Constant | 0.598*** | 0.340*** | 0.023*** | 0.036*** |
|  | (0.042) | (0.040) | (0.004) | (0.007) |
|  |  |  |  |  |
| R^2^ | 0.770 | 0.810 | 0.018 | 0.033 |
| N | 300 | 300 | 300 | 300 |

Notes: Cell entries are regression coefficients predicting each candidate’s vote share using the predictors listed in the first column with standard errors in parentheses.

***p<.001 **p<.01 *p<.05 +p<.10

Table SC39

*Name Order Effects in the 2016 U.S. Senate Election in New Hampshire: Name Order and Name Order Squared (With Weights)*

|  | Dependent Variable | | | |
| --- | --- | --- | --- | --- |
| Predictor | Vote share of the Democratic candidate (Maggie Hassan)  (1) | Vote share of the Republican candidate (Kelly Ayotte)  (2) | Vote share of the other candidate (Brian Chabot, Libertarian)  (3) | Vote share of the other candidate (Aaron Day, Independent)  (4) |
| Name order | -0.007* | -0.004+ | -0.000 | -0.001 |
|  | (0.003) | (0.003) | (0.000) | (0.001) |
| Name order squared | 0.000 | 0.007+ | -0.002* | -0.002+ |
|  | (0.005) | (0.004) | (0.001) | (0.001) |
| % Democratic registered voters | 0.388*** | -0.361*** | -0.005 | -0.026* |
|  | (0.089) | (0.084) | (0.008) | (0.010) |
| % Republican registered voters | -0.751*** | 0.783*** | -0.015+ | -0.013 |
|  | (0.068) | (0.066) | (0.008) | (0.015) |
| Total number of votes (in 000s) | 0.001 | -0.001 | -0.000 | -0.000 |
|  | (0.001) | (0.001) | (0.000) | (0.000) |
| Constant | 0.598*** | 0.337*** | 0.025*** | 0.038*** |
|  | (0.042) | (0.040) | (0.004) | (0.007) |
|  |  |  |  |  |
| R^2^ | 0.770 | 0.812 | 0.030 | 0.044 |
| N | 300 | 300 | 300 | 300 |

Notes: Cell entries are regression coefficients predicting each candidate’s vote share using the predictors listed in the first column with standard errors in parentheses.

***p<.001 **p<.01 *p<.05 +p<.10

Table SC40

*Name Order Effects in the 2016 Gubernatorial Election in New Hampshire: First Versus Later (With Weights)*

|  | Dependent Variable | | |
| --- | --- | --- | --- |
| Predictor | Vote share of the Democratic candidate (Colin Van Ostern)  (1) | Vote share of the Republican candidate (Chris Sununu)  (2) | Vote share of the other candidate (Max Abramson, Libertarian)  (3) |
| Listed first (vs. later) | 0.009+ | 0.014** | -0.001 |
|  | (0.005) | (0.005) | (0.002) |
| % Democratic registered voters | 0.416*** | -0.386*** | -0.033* |
|  | (0.092) | (0.085) | (0.016) |
| % Republican registered voters | -0.701*** | 0.754*** | -0.042* |
|  | (0.068) | (0.063) | (0.017) |
| Total number of votes (in 000s) | -0.001 | 0.001 | -0.000 |
|  | (0.001) | (0.001) | (0.000) |
| Constant | 0.568*** | 0.355*** | 0.067*** |
|  | (0.041) | (0.040) | (0.009) |
|  |  |  |  |
| R^2^ | 0.730 | 0.771 | 0.032 |
| N | 300 | 300 | 300 |

Notes: Cell entries are regression coefficients predicting each candidate’s vote share using the predictors listed in the first column with standard errors in parentheses.

***p<.001 **p<.01 *p<.05 +p<.10

Table SC41

*Name Order Effects in the 2016 Gubernatorial Election in New Hampshire: Second/Third Versus First (With Weights)*

|  | Dependent Variable | | |
| --- | --- | --- | --- |
| Predictor | Vote share of the Democratic candidate (Colin Van Ostern)  (1) | Vote share of the Republican candidate (Chris Sununu)  (2) | Vote share of the other candidate (Max Abramson, Libertarian)  (3) |
| Listed second (vs. first) | -0.007 | -0.013* | 0.007** |
|  | (0.006) | (0.006) | (0.002) |
| Listed third (vs. first) | -0.013+ | -0.016*** | -0.005* |
|  | (0.007) | (0.006) | (0.002) |
| % Democratic registered voters | 0.418*** | -0.385*** | -0.039** |
|  | (0.090) | (0.085) | (0.015) |
| % Republican registered voters | -0.700*** | 0.756*** | -0.052** |
|  | (0.067) | (0.064) | (0.017) |
| Total number of votes (in 000s) | -0.001 | 0.001 | -0.000 |
|  | (0.001) | (0.001) | (0.000) |
| Constant | 0.576*** | 0.369*** | 0.071*** |
|  | (0.042) | (0.038) | (0.009) |
|  |  |  |  |
| R^2^ | 0.731 | 0.772 | 0.122 |
| N | 300 | 300 | 300 |

Notes: Cell entries are regression coefficients predicting each candidate’s vote share using the predictors listed in the first column with standard errors in parentheses.

***p<.001 **p<.01 *p<.05 +p<.10

Table SC42

*Name Order Effects in the 2016 Gubernatorial Election in New Hampshire: Name Order (With Weights)*

|  | Dependent Variable | | |
| --- | --- | --- | --- |
| Predictor | Vote share of the Democratic candidate (Colin Van Ostern)  (1) | Vote share of the Republican candidate (Chris Sununu)  (2) | Vote share of the other candidate (Max Abramson, Libertarian)  (3) |
| Name order | -0.006+ | -0.008** | -0.002* |
|  | (0.003) | (0.003) | (0.001) |
| % Democratic registered voters | 0.418*** | -0.383*** | -0.033* |
|  | (0.091) | (0.086) | (0.016) |
| % Republican registered voters | -0.700*** | 0.757*** | -0.047** |
|  | (0.067) | (0.063) | (0.017) |
| Total number of votes (in 000s) | -0.001 | 0.001 | -0.000 |
|  | (0.001) | (0.001) | (0.000) |
| Constant | 0.569*** | 0.359*** | 0.068*** |
|  | (0.041) | (0.039) | (0.009) |
|  |  |  |  |
| R^2^ | 0.731 | 0.771 | 0.048 |
| N | 300 | 300 | 300 |

Notes: Cell entries are regression coefficients predicting each candidate’s vote share using the predictors listed in the first column with standard errors in parentheses.

***p<.001 **p<.01 *p<.05 +p<.10

Table SC43

*Name Order Effects in the 2016 Gubernatorial Election in New Hampshire: Name Order and Name Order Squared (With Weights)*

|  | Dependent Variable | | |
| --- | --- | --- | --- |
| Predictor | Vote share of the Democratic candidate (Colin Van Ostern)  (1) | Vote share of the Republican candidate (Chris Sununu)  (2) | Vote share of the other candidate (Max Abramson, Libertarian)  (3) |
| Name order | -0.006+ | -0.008** | -0.002* |
|  | (0.003) | (0.003) | (0.001) |
| Name order squared | 0.000 | 0.005 | -0.009*** |
|  | (0.005) | (0.005) | (0.002) |
| % Democratic registered voters | 0.418*** | -0.385*** | -0.039** |
|  | (0.090) | (0.085) | (0.015) |
| % Republican registered voters | -0.700*** | 0.756*** | -0.052** |
|  | (0.067) | (0.064) | (0.017) |
| Total number of votes (in 000s) | -0.001 | 0.001 | -0.000 |
|  | (0.001) | (0.001) | (0.000) |
| Constant | 0.569*** | 0.356*** | 0.078*** |
|  | (0.042) | (0.040) | (0.009) |
|  |  |  |  |
| R^2^ | 0.731 | 0.772 | 0.122 |
| N | 300 | 300 | 300 |

Notes: Cell entries are regression coefficients predicting each candidate’s vote share using the predictors listed in the first column with standard errors in parentheses.

***p<.001 **p<.01 *p<.05 +p<.10

Table SC44

*Name Order Effects in the 2016 Congressional District 1 Election in New Hampshire: First Versus Later (With Weights)*

|  | Dependent Variable | | | | |
| --- | --- | --- | --- | --- | --- |
| Predictor | Vote share of the Democratic candidate (Carol Shea-Porter)  (1) | Vote share of the Republican candidate (Frank Guinta)  (2) | Vote share of the other candidate (Robert Lombardo, Libertarian)  (3) | Vote share of the other candidate (Brendan Kelly, Independent)  (4) | Vote share of the other candidate (Shawn P. O’Connor, Independent)  (5) |
| Listed first (vs. later) | 0.007 | 0.003 | 0.001 | -0.001 | -0.001 |
|  | (0.007) | (0.006) | (0.001) | (0.001) | (0.003) |
| % Democratic registered voters | 0.559*** | -0.490*** | -0.004 | -0.021* | -0.048 |
|  | (0.127) | (0.129) | (0.010) | (0.009) | (0.043) |
| % Republican registered voters | -0.560*** | 0.639*** | -0.017 | -0.013 | -0.042 |
|  | (0.121) | (0.125) | (0.011) | (0.010) | (0.044) |
| Total number of votes (in 000s) | 0.000 | -0.000 | 0.000 | 0.000 | -0.000 |
|  | (0.001) | (0.001) | (0.000) | (0.000) | (0.001) |
| Constant | 0.464*** | 0.361*** | 0.020*** | 0.026*** | 0.124*** |
|  | (0.069) | (0.073) | (0.006) | (0.005) | (0.025) |
|  |  |  |  |  |  |
| R^2^ | 0.815 | 0.838 | 0.052 | 0.041 | 0.023 |
| N | 114 | 114 | 114 | 114 | 114 |

Notes: Cell entries are regression coefficients predicting each candidate’s vote share using the predictors listed in the first column with standard errors in parentheses.

***p<.001 **p<.01 *p<.05 +p<.10

Table SC45

*Name Order Effects in the 2016 Congressional District 1 Election in New Hampshire: Second/Third Versus First (With Weights)*

|  | Dependent Variable | | | | |
| --- | --- | --- | --- | --- | --- |
| Predictor | Vote share of the Democratic candidate (Carol Shea-Porter)  (1) | Vote share of the Republican candidate (Frank Guinta)  (2) | Vote share of the other candidate (Robert Lombardo, Libertarian)  (3) | Vote share of the other candidate (Brendan Kelly, Independent)  (4) | Vote share of the other candidate (Shawn P. O’Connor, Independent)  (5) |
| Listed second (vs. first) | -0.007 | -0.004 | 0.000 | 0.002* | 0.009* |
|  | (0.008) | (0.008) | (0.001) | (0.001) | (0.004) |
| Listed third (vs. first) | -0.007 | -0.003 | -0.002+ | -0.001 | -0.007+ |
|  | (0.009) | (0.007) | (0.001) | (0.001) | (0.004) |
| % Democratic registered voters | 0.559*** | -0.490*** | -0.004 | -0.022** | -0.050 |
|  | (0.128) | (0.131) | (0.010) | (0.008) | (0.040) |
| % Republican registered voters | -0.560*** | 0.638*** | -0.018+ | -0.015 | -0.051 |
|  | (0.121) | (0.128) | (0.011) | (0.010) | (0.041) |
| Total number of votes (in 000s) | 0.000 | -0.000 | 0.000 | 0.000 | -0.001 |
|  | (0.001) | (0.001) | (0.000) | (0.000) | (0.001) |
| Constant | 0.471*** | 0.364*** | 0.022*** | 0.027*** | 0.128*** |
|  | (0.071) | (0.075) | (0.005) | (0.005) | (0.023) |
|  |  |  |  |  |  |
| R^2^ | 0.815 | 0.838 | 0.077 | 0.108 | 0.154 |
| N | 114 | 114 | 114 | 114 | 114 |

Notes: Cell entries are regression coefficients predicting each candidate’s vote share using the predictors listed in the first column with standard errors in parentheses.

***p<.001 **p<.01 *p<.05 +p<.10

Table SC46

*Name Order Effects in the 2016 Congressional District 1 Election in New Hampshire: Name Order (With Weights)*

|  | Dependent Variable | | | | |
| --- | --- | --- | --- | --- | --- |
| Predictor | Vote share of the Democratic candidate (Carol Shea-Porter)  (1) | Vote share of the Republican candidate (Frank Guinta)  (2) | Vote share of the other candidate (Robert Lombardo, Libertarian)  (3) | Vote share of the other candidate (Brendan Kelly, Independent)  (4) | Vote share of the other candidate (Shawn P. O’Connor, Independent)  (5) |
| Name order | -0.004 | -0.001 | -0.001+ | -0.001 | -0.003+ |
|  | (0.004) | (0.003) | (0.001) | (0.001) | (0.002) |
| % Democratic registered voters | 0.560*** | -0.491*** | -0.004 | -0.021* | -0.048 |
|  | (0.127) | (0.130) | (0.010) | (0.009) | (0.042) |
| % Republican registered voters | -0.560*** | 0.638*** | -0.018+ | -0.015 | -0.053 |
|  | (0.120) | (0.127) | (0.011) | (0.010) | (0.042) |
| Total number of votes (in 000s) | 0.000 | -0.000 | 0.000 | 0.000 | -0.000 |
|  | (0.001) | (0.001) | (0.000) | (0.000) | (0.001) |
| Constant | 0.466*** | 0.362*** | 0.021*** | 0.027*** | 0.127*** |
|  | (0.069) | (0.074) | (0.006) | (0.005) | (0.024) |
|  |  |  |  |  |  |
| R^2^ | 0.815 | 0.837 | 0.067 | 0.045 | 0.046 |
| N | 114 | 114 | 114 | 114 | 114 |

Notes: Cell entries are regression coefficients predicting each candidate’s vote share using the predictors listed in the first column with standard errors in parentheses.

***p<.001 **p<.01 *p<.05 +p<.10

Table SC47

*Name Order Effects in the 2016 Congressional District 1 Election in New Hampshire: Name Order and Name Order Squared (With Weights)*

|  | Dependent Variable | | | | |
| --- | --- | --- | --- | --- | --- |
| Predictor | Vote share of the Democratic candidate (Carol Shea-Porter)  (1) | Vote share of the Republican candidate (Frank Guinta)  (2) | Vote share of the other candidate (Robert Lombardo, Libertarian)  (3) | Vote share of the other candidate (Brendan Kelly, Independent)  (4) | Vote share of the other candidate (Shawn P. O’Connor, Independent)  (5) |
| Name order | -0.004 | -0.001 | -0.001+ | -0.001 | -0.004+ |
|  | (0.004) | (0.003) | (0.001) | (0.001) | (0.002) |
| Name order squared | 0.003 | 0.002 | -0.001 | -0.003** | -0.013*** |
|  | (0.007) | (0.007) | (0.001) | (0.001) | (0.003) |
| % Democratic registered voters | 0.559*** | -0.490*** | -0.004 | -0.022** | -0.050 |
|  | (0.128) | (0.131) | (0.010) | (0.008) | (0.040) |
| % Republican registered voters | -0.560*** | 0.638*** | -0.018+ | -0.015 | -0.051 |
|  | (0.121) | (0.128) | (0.011) | (0.010) | (0.041) |
| Total number of votes (in 000s) | 0.000 | -0.000 | 0.000 | 0.000 | -0.001 |
|  | (0.001) | (0.001) | (0.000) | (0.000) | (0.001) |
| Constant | 0.464*** | 0.361*** | 0.022*** | 0.029*** | 0.138*** |
|  | (0.071) | (0.072) | (0.006) | (0.005) | (0.023) |
|  |  |  |  |  |  |
| R^2^ | 0.815 | 0.838 | 0.077 | 0.108 | 0.154 |
| N | 114 | 114 | 114 | 114 | 114 |

Notes: Cell entries are regression coefficients predicting each candidate’s vote share using the predictors listed in the first column with standard errors in parentheses.

***p<.001 **p<.01 *p<.05 +p<.10

Table SC48

*Name Order Effects in the 2016 Congressional District 2 Election in New Hampshire: First Versus Later (With Weights)*

|  | Dependent Variable | | |
| --- | --- | --- | --- |
| Predictor | Vote share of the Democratic candidate (Ann McLane Kuster)  (1) | Vote share of the Republican candidate (Jim Lawrence)  (2) | Vote share of the other candidate (John J. Babiarz, Independent)  (3) |
| Listed first (vs. later) | 0.012+ | 0.020** | -0.000 |
|  | (0.007) | (0.006) | (0.003) |
| % Democratic registered voters | 0.401** | -0.382*** | -0.025 |
|  | (0.126) | (0.105) | (0.025) |
| % Republican registered voters | -0.734*** | 0.773*** | -0.031 |
|  | (0.089) | (0.078) | (0.023) |
| Total number of votes (in 000s) | 0.000 | -0.000 | -0.000 |
|  | (0.002) | (0.002) | (0.001) |
| Constant | 0.595*** | 0.328*** | 0.066*** |
|  | (0.054) | (0.047) | (0.013) |
|  |  |  |  |
| R^2^ | 0.717 | 0.787 | 0.012 |
| N | 186 | 186 | 186 |

Notes: Cell entries are regression coefficients predicting each candidate’s vote share using the predictors listed in the first column with standard errors in parentheses.

***p<.001 **p<.01 *p<.05 +p<.10

Table SC49

*Name Order Effects in the 2016 Congressional District 2 Election in New Hampshire: Second/Third Versus First (With Weights)*

|  | Dependent Variable | | |
| --- | --- | --- | --- |
| Predictor | Vote share of the Democratic candidate (Ann McLane Kuster)  (1) | Vote share of the Republican candidate (Jim Lawrence)  (2) | Vote share of the other candidate (John J. Babiarz, Independent)  (3) |
| Listed second (vs. first) | -0.006 | -0.016* | 0.007* |
|  | (0.007) | (0.007) | (0.003) |
| Listed third (vs. first) | -0.020* | -0.023** | -0.005* |
|  | (0.009) | (0.007) | (0.003) |
| % Democratic registered voters | 0.406** | -0.380*** | -0.034 |
|  | (0.123) | (0.105) | (0.024) |
| % Republican registered voters | -0.729*** | 0.778*** | -0.043+ |
|  | (0.088) | (0.077) | (0.024) |
| Total number of votes (in 000s) | 0.000 | -0.000 | 0.000 |
|  | (0.002) | (0.002) | (0.001) |
| Constant | 0.604*** | 0.346*** | 0.071*** |
|  | (0.054) | (0.045) | (0.013) |
|  |  |  |  |
| R^2^ | 0.721 | 0.788 | 0.093 |
| N | 186 | 186 | 186 |

Notes: Cell entries are regression coefficients predicting each candidate’s vote share using the predictors listed in the first column with standard errors in parentheses.

***p<.001 **p<.01 *p<.05 +p<.10

Table SC50

*Name Order Effects in the 2016 Congressional District 2 Election in New Hampshire: Name Order (With Weights)*

|  | Dependent Variable | | |
| --- | --- | --- | --- |
| Predictor | Vote share of the Democratic candidate (Ann McLane Kuster)  (1) | Vote share of the Republican candidate (Jim Lawrence)  (2) | Vote share of the other candidate (John J. Babiarz, Independent)  (3) |
| Name order | -0.010* | -0.012** | -0.003* |
|  | (0.004) | (0.004) | (0.001) |
| % Democratic registered voters | 0.405** | -0.378*** | -0.026 |
|  | (0.124) | (0.105) | (0.025) |
| % Republican registered voters | -0.731*** | 0.779*** | -0.035 |
|  | (0.088) | (0.078) | (0.023) |
| Total number of votes (in 000s) | 0.000 | -0.000 | -0.000 |
|  | (0.002) | (0.002) | (0.000) |
| Constant | 0.597*** | 0.332*** | 0.067*** |
|  | (0.054) | (0.047) | (0.013) |
|  |  |  |  |
| R^2^ | 0.720 | 0.787 | 0.029 |
| N | 186 | 186 | 186 |

Notes: Cell entries are regression coefficients predicting each candidate’s vote share using the predictors listed in the first column with standard errors in parentheses.

***p<.001 **p<.01 *p<.05 +p<.10

Table SC51

*Name Order Effects in the 2016 Congressional District 2 Election in New Hampshire: Name Order and Name Order Squared (With Weights)*

|  | Dependent Variable | | |
| --- | --- | --- | --- |
| Predictor | Vote share of the Democratic candidate (Ann McLane Kuster)  (1) | Vote share of the Republican candidate (Jim Lawrence)  (2) | Vote share of the other candidate (John J. Babiarz, Independent)  (3) |
| Name order | -0.010* | -0.012** | -0.003* |
|  | (0.004) | (0.004) | (0.001) |
| Name order squared | -0.004 | 0.004 | -0.010** |
|  | (0.007) | (0.005) | (0.003) |
| % Democratic registered voters | 0.406** | -0.380*** | -0.034 |
|  | (0.123) | (0.105) | (0.024) |
| % Republican registered voters | -0.729*** | 0.778*** | -0.043+ |
|  | (0.088) | (0.077) | (0.024) |
| Total number of votes (in 000s) | 0.000 | -0.000 | 0.000 |
|  | (0.002) | (0.002) | (0.001) |
| Constant | 0.598*** | 0.330*** | 0.078*** |
|  | (0.054) | (0.047) | (0.013) |
|  |  |  |  |
| R^2^ | 0.721 | 0.788 | 0.093 |
| N | 186 | 186 | 186 |

Notes: Cell entries are regression coefficients predicting each candidate’s vote share using the predictors listed in the first column with standard errors in parentheses.

***p<.001 **p<.01 *p<.05 +p<.10

Table SC52

*Name Order Effects in the 2012 Presidential Election in New Hampshire: Name Order with Alternative Name Order Coding*

|  | Dependent Variable | | | |
| --- | --- | --- | --- | --- |
| Predictor | Vote share of the Democratic candidate (Barack Obama)  (1) | Vote share of the Republican candidate (Mitt Romney)  (2) | Vote share of the other candidate (Gary Johnson, Libertarian)  (3) | Vote share of the other candidate (Virgil Goode, Constitution)  (4) |
| Name order | -0.001 | -0.001 | -0.000 | 0.000 |
|  | (0.002) | (0.002) | (0.000) | (0.000) |
| % Democratic registered voters | 0.608*** | -0.575*** | -0.023*** | -0.010*** |
|  | (0.048) | (0.047) | (0.006) | (0.003) |
| % Republican registered voters | -0.575*** | 0.612*** | -0.030*** | -0.009*** |
|  | (0.051) | (0.049) | (0.007) | (0.003) |
| Total number of votes (in 000s) | -0.007*** | 0.007*** | -0.000 | -0.000 |
|  | (0.001) | (0.001) | (0.000) | (0.000) |
| Constant | 0.562*** | 0.404*** | 0.027*** | 0.007*** |
|  | (0.024) | (0.023) | (0.003) | (0.001) |
|  |  |  |  |  |
| R^2^ | 0.725 | 0.736 | 0.084 | 0.072 |
| N | 300 | 300 | 300 | 300 |

Notes: Cell entries are regression coefficients predicting each candidate’s vote share using the predictors listed in the first column with standard errors in parentheses. The linear name order was coded such as the multiple other party candidates to have sequential name order, in ascending order of the vertical display position.

***p<.001 **p<.01 *p<.05 +p<.10

Table SC53

*Name Order Effects in the 2012 Presidential Election in New Hampshire: Name Order and Name Order Squared with Alternative Name Order Coding*

|  | Dependent Variable | | | |
| --- | --- | --- | --- | --- |
| Predictor | Vote share of the Democratic candidate (Barack Obama)  (1) | Vote share of the Republican candidate (Mitt Romney)  (2) | Vote share of the other candidate (Gary Johnson, Libertarian)  (3) | Vote share of the other candidate (Virgil Goode, Constitution)  (4) |
| Name order | -0.001 | -0.000 | -0.000 | -0.000 |
|  | (0.003) | (0.003) | (0.000) | (0.000) |
| Name order squared | -0.004 | -0.005 | -0.001 | -0.001 |
|  | (0.006) | (0.006) | (0.001) | (0.001) |
| % Democratic registered voters | 0.609*** | -0.576*** | -0.023*** | -0.023*** |
|  | (0.048) | (0.047) | (0.006) | (0.006) |
| % Republican registered voters | -0.575*** | 0.610*** | -0.030*** | -0.030*** |
|  | (0.051) | (0.049) | (0.007) | (0.007) |
| Total number of votes (in 000s) | -0.007*** | 0.007*** | -0.000 | -0.000 |
|  | (0.001) | (0.001) | (0.000) | (0.000) |
| Constant | 0.564*** | 0.408*** | 0.028*** | 0.028*** |
|  | (0.024) | (0.024) | (0.003) | (0.003) |
|  |  |  |  |  |
| R^2^ | 0.725 | 0.736 | 0.087 | 0.087 |
| N | 300 | 300 | 300 | 300 |

Notes: Cell entries are regression coefficients predicting each candidate’s vote share using the predictors listed in the first column with standard errors in parentheses. The linear and quadratic name order were coded such as the multiple other party candidates to have sequential name order, in ascending order of the vertical display position.

***p<.001 **p<.01 *p<.05 +p<.10

Table SC54

*Name Order Effects in the 2016 Presidential Election in New Hampshire: Name Order with Alternative Name Order Coding*

|  | Dependent Variable | | | | |
| --- | --- | --- | --- | --- | --- |
| Predictor | Vote share of the Democratic candidate (Hillary Clinton)  (1) | Vote share of the Republican candidate (Donald J. Trump)  (2) | Vote share of the other candidate (Gary Johnson, Libertarian)  (3) | Vote share of the other candidate (Jill Stein, Green)  (4) | Vote share of the other candidate (Roque “Rocky” De La Fuente, American Delta)  (5) |
| Name order | -0.004+ | -0.004+ | 0.000 | -0.001+ | -0.000+ |
|  | (0.002) | (0.002) | (0.001) | (0.000) | (0.000) |
| % Democratic registered voters | 0.445*** | -0.464*** | 0.017 | 0.009* | -0.001 |
|  | (0.059) | (0.058) | (0.011) | (0.004) | (0.002) |
| % Republican registered voters | -0.651*** | 0.686*** | 0.002 | -0.017** | -0.003 |
|  | (0.069) | (0.068) | (0.013) | (0.005) | (0.002) |
| Total number of votes (in 000s) | 0.004** | -0.003** | 0.000 | -0.000*** | -0.000 |
|  | (0.002) | (0.002) | (0.000) | (0.000) | (0.000) |
| Constant | 0.531*** | 0.406*** | 0.036*** | 0.013*** | 0.003* |
|  | (0.034) | (0.033) | (0.006) | (0.003) | (0.001) |
|  |  |  |  |  |  |
| R^2^ | 0.581 | 0.606 | 0.013 | 0.154 | 0.021 |
| N | 300 | 300 | 300 | 300 | 300 |

Notes: Cell entries are regression coefficients predicting each candidate’s vote share using the predictors listed in the first column with standard errors in parentheses. The linear name order was coded such as the multiple other party candidates to have sequential name order, in ascending order of the vertical display position.

***p<.001 **p<.01 *p<.05 +p<.10

Table SC55

*Name Order Effects in the 2016 Presidential Election in New Hampshire: Name Order and Name Order Squared with Alternative Name Order Coding*

|  | Dependent Variable | | | | |
| --- | --- | --- | --- | --- | --- |
| Predictor | Vote share of the Democratic candidate (Hillary Clinton)  (1) | Vote share of the Republican candidate (Donald J. Trump)  (2) | Vote share of the other candidate (Gary Johnson, Libertarian)  (3) | Vote share of the other candidate (Jill Stein, Green)  (4) | Vote share of the other candidate (Roque “Rocky” De La Fuente, American Delta)  (5) |
| Name order | -0.005+ | -0.005+ | 0.002 | 0.000 | -0.000+ |
|  | (0.002) | (0.002) | (0.003) | (0.001) | (0.000) |
| Name order squared | 0.002 | 0.003 | -0.001 | 0.000 | 0.000 |
|  | (0.003) | (0.003) | (0.002) | (0.001) | (0.000) |
| % Democratic registered voters | 0.444*** | -0.469*** | 0.016 | 0.010* | -0.001 |
|  | (0.059) | (0.058) | (0.011) | (0.005) | (0.002) |
| % Republican registered voters | -0.652*** | 0.684*** | 0.001 | -0.017** | -0.003 |
|  | (0.069) | (0.068) | (0.013) | (0.005) | (0.002) |
| Total number of votes (in 000s) | 0.004** | -0.003* | 0.000 | -0.000*** | -0.000 |
|  | (0.002) | (0.002) | (0.000) | (0.000) | (0.000) |
| Constant | 0.526*** | 0.398*** | 0.036*** | 0.013*** | 0.002* |
|  | (0.035) | (0.034) | (0.006) | (0.003) | (0.001) |
|  |  |  |  |  |  |
| R^2^ | 0.582 | 0.607 | 0.013 | 0.156 | 0.022 |
| N | 300 | 300 | 300 | 300 | 300 |

Notes: Cell entries are regression coefficients predicting each candidate’s vote share using the predictors listed in the first column with standard errors in parentheses. The linear and quadratic name order were coded such as the multiple other party candidates to have sequential name order, in ascending order of the vertical display position.

***p<.001 **p<.01 *p<.05 +p<.10

Table SC56

*Name Order Effects in the 2016 U.S. Senate Election in New Hampshire: Name Order with Alternative Name Order Coding*

|  | Dependent Variable | | | |
| --- | --- | --- | --- | --- |
| Predictor | Vote share of the Democratic candidate (Maggie Hassan)  (1) | Vote share of the Republican candidate (Kelly Ayotte)  (2) | Vote share of the other candidate (Brian Chabot, Libertarian)  (3) | Vote share of the other candidate (Aaron Day, Independent)  (4) |
| Name order | -0.006** | -0.002 | -0.000 | -0.001 |
|  | (0.002) | (0.002) | (0.001) | (0.001) |
| % Democratic registered voters | 0.290*** | -0.277*** | 0.003 | -0.012 |
|  | (0.042) | (0.037) | (0.006) | (0.010) |
| % Republican registered voters | -0.778*** | 0.795*** | -0.013+ | 0.004 |
|  | (0.049) | (0.044) | (0.007) | (0.012) |
| Total number of votes (in 000s) | 0.001 | -0.001 | 0.000 | -0.000 |
|  | (0.001) | (0.001) | (0.000) | (0.000) |
| Constant | 0.632*** | 0.315*** | 0.020*** | 0.028*** |
|  | (0.024) | (0.021) | (0.004) | (0.006) |
|  |  |  |  |  |
| R^2^ | 0.718 | 0.762 | 0.028 | 0.022 |
| N | 300 | 300 | 300 | 300 |

Notes: Cell entries are regression coefficients predicting each candidate’s vote share using the predictors listed in the first column with standard errors in parentheses. The linear name order was coded such as the multiple other party candidates to have sequential name order, in ascending order of the vertical display position.

***p<.001 **p<.01 *p<.05 +p<.10

Table SC57

*Name Order Effects in the 2016 U.S. Senate Election in New Hampshire: Name Order and Name Order Squared with Alternative Name Order Coding*

|  | Dependent Variable | | | |
| --- | --- | --- | --- | --- |
| Predictor | Vote share of the Democratic candidate (Maggie Hassan)  (1) | Vote share of the Republican candidate (Kelly Ayotte)  (2) | Vote share of the other candidate (Brian Chabot, Libertarian)  (3) | Vote share of the other candidate (Aaron Day, Independent)  (4) |
| Name order | -0.007** | -0.003 | -0.002 | 0.001 |
|  | (0.002) | (0.002) | (0.001) | (0.002) |
| Name order squared | 0.003 | 0.005* | -0.001 | -0.002+ |
|  | (0.003) | (0.003) | (0.001) | (0.001) |
| % Democratic registered voters | 0.289*** | -0.282*** | 0.002 | -0.014 |
|  | (0.042) | (0.037) | (0.006) | (0.010) |
| % Republican registered voters | -0.779*** | 0.793*** | -0.014* | 0.003 |
|  | (0.049) | (0.044) | (0.007) | (0.012) |
| Total number of votes (in 000s) | 0.001 | -0.001 | 0.000 | -0.000 |
|  | (0.001) | (0.001) | (0.000) | (0.000) |
| Constant | 0.629*** | 0.308*** | 0.021*** | 0.030*** |
|  | (0.024) | (0.021) | (0.004) | (0.006) |
|  |  |  |  |  |
| R^2^ | 0.718 | 0.765 | 0.036 | 0.031 |
| N | 300 | 300 | 300 | 300 |

Notes: Cell entries are regression coefficients predicting each candidate’s vote share using the predictors listed in the first column with standard errors in parentheses. The linear and quadratic name order were coded such as the multiple other party candidates to have sequential name order, in ascending order of the vertical display position

***p<.001 **p<.01 *p<.05 +p<.10

Table SC58

*Name Order Effects in the 2016 Congressional District 1 Election in New Hampshire: Name Order with Alternative Name Order Coding*

|  | Dependent Variable | | | | |
| --- | --- | --- | --- | --- | --- |
| Predictor | Vote share of the Democratic candidate (Carol Shea-Porter)  (1) | Vote share of the Republican candidate (Frank Guinta)  (2) | Vote share of the other candidate (Robert Lombardo, Libertarian)  (3) | Vote share of the other candidate (Brendan Kelly, Independent)  (4) | Vote share of the other candidate (Shawn P. O’Connor, Independent)  (5) |
| Name order | -0.002 | -0.001 | -0.001+ | -0.001 | -0.004+ |
|  | (0.002) | (0.002) | (0.001) | (0.001) | (0.002) |
| % Democratic registered voters | 0.559*** | -0.496*** | -0.002 | -0.020+ | -0.043 |
|  | (0.072) | (0.068) | (0.010) | (0.011) | (0.039) |
| % Republican registered voters | -0.559*** | 0.629*** | -0.016 | -0.015 | -0.050 |
|  | (0.069) | (0.066) | (0.010) | (0.011) | (0.038) |
| Total number of votes (in 000s) | 0.000 | 0.000 | 0.000 | 0.000 | -0.001 |
|  | (0.001) | (0.001) | (0.000) | (0.000) | (0.001) |
| Constant | 0.467*** | 0.365*** | 0.020*** | 0.026*** | 0.122*** |
|  | (0.038) | (0.037) | (0.006) | (0.006) | (0.021) |
|  |  |  |  |  |  |
| R^2^ | 0.816 | 0.833 | 0.061 | 0.043 | 0.049 |
| N | 114 | 114 | 114 | 114 | 114 |

Notes: Cell entries are regression coefficients predicting each candidate’s vote share using the predictors listed in the first column with standard errors in parentheses. The linear name order was coded such as the multiple other party candidates to have sequential name order, in ascending order of the vertical display position.

***p<.001 **p<.01 *p<.05 +p<.10

Table SC59

*Name Order Effects in the 2016 Congressional District 1 Election in New Hampshire: Name Order and Name Order Squared with Alternative Name Order Coding*

|  | Dependent Variable | | | | |
| --- | --- | --- | --- | --- | --- |
| Predictor | Vote share of the Democratic candidate (Carol Shea-Porter)  (1) | Vote share of the Republican candidate (Frank Guinta)  (2) | Vote share of the other candidate (Robert Lombardo, Libertarian)  (3) | Vote share of the other candidate (Brendan Kelly, Independent)  (4) | Vote share of the other candidate (Shawn P. O’Connor, Independent)  (5) |
| Name order | -0.002 | -0.001 | 0.000 | -0.001 | -0.032*** |
|  | (0.002) | (0.002) | (0.002) | (0.001) | (0.008) |
| Name order squared | 0.002 | 0.001 | -0.001 | -0.003* | -0.014*** |
|  | (0.002) | (0.002) | (0.001) | (0.001) | (0.004) |
| % Democratic registered voters | 0.557*** | -0.495*** | -0.002 | -0.020* | -0.046 |
|  | (0.072) | (0.068) | (0.010) | (0.011) | (0.037) |
| % Republican registered voters | -0.558*** | 0.630*** | -0.015 | -0.014 | -0.047 |
|  | (0.069) | (0.066) | (0.010) | (0.011) | (0.036) |
| Total number of votes (in 000s) | 0.000 | 0.000 | 0.000 | 0.000 | -0.001 |
|  | (0.001) | (0.001) | (0.000) | (0.000) | (0.001) |
| Constant | 0.463*** | 0.360*** | 0.020*** | 0.028*** | 0.119*** |
|  | (0.039) | (0.038) | (0.006) | (0.006) | (0.020) |
|  |  |  |  |  |  |
| R^2^ | 0.816 | 0.833 | 0.065 | 0.094 | 0.154 |
| N | 114 | 114 | 114 | 114 | 114 |

Notes: Cell entries are regression coefficients predicting each candidate’s vote share using the predictors listed in the first column with standard errors in parentheses. The linear and quadratic name order were coded such as the multiple other party candidates to have sequential name order, in ascending order of the vertical display position.

***p<.001 **p<.01 *p<.05 +p<.10
